# *Oceanospirillales* containing the DMSP lyase DddD are key utilisers of carbon from DMSP in coastal seawater

**DOI:** 10.1186/s40168-022-01304-0

**Published:** 2022-07-27

**Authors:** Jingli Liu, Chun-Xu Xue, Jinyan Wang, Andrew T. Crombie, Ornella Carrión, Andrew W. B. Johnston, J. Colin Murrell, Ji Liu, Yanfen Zheng, Xiao-Hua Zhang, Jonathan D. Todd

**Affiliations:** 1 Frontiers Science Center for Deep Ocean Multispheres and Earth System, and College of Marine Life Sciences , Ocean University of China, Qingdao, China; 2 School of Biological Sciences, University of East Anglia, Norwich Research Park, Norwich, UK; 3 School of Environmental Sciences, University of East Anglia, Norwich Research Park, Norwich, UK; 4 Laboratory for Marine Ecology and Environmental Science, Qingdao National Laboratory for Marine Science and Technology, Qingdao, China

**Keywords:** Dimethylsulfoniopropionate (DMSP), Dimethylsulfide (DMS), DNA-stable isotope probing (DNA-SIP), *Oceanospirillales*, DddD DMSP lyase, Seawater, Biogeochemical sulfur cycling

## Abstract

**Background:**

Ubiquitous and diverse marine microorganisms utilise the abundant organosulfur molecule dimethylsulfoniopropionate (DMSP), the main precursor of the climate-active gas dimethylsulfide (DMS), as a source of carbon, sulfur and/or signalling molecules. However, it is currently difficult to discern which microbes actively catabolise DMSP in the environment, why they do so and the pathways used.

**Results:**

Here, a novel DNA-stable isotope probing (SIP) approach, where only the propionate and not the DMS moiety of DMSP was ^13^C-labelled, was strategically applied to identify key microorganisms actively using DMSP and also likely DMS as a carbon source, and their catabolic enzymes, in North Sea water. Metagenomic analysis of natural seawater suggested that *Rhodobacterales* (*Roseobacter* group) and SAR11 bacteria were the major microorganisms degrading DMSP via demethylation and, to a lesser extent, DddP-driven DMSP lysis pathways. However, neither *Rhodobacterales* and SAR11 bacteria nor their DMSP catabolic genes were prominently labelled in DNA-SIP experiments, suggesting they use DMSP as a sulfur source and/or in signalling pathways, and not primarily for carbon requirements. Instead, DNA-SIP identified gammaproteobacterial *Oceanospirillales*, e.g. *Amphritea*, and their DMSP lyase DddD as the dominant microorganisms/enzymes using DMSP as a carbon source. Supporting this, most gammaproteobacterial (with DddD) but few alphaproteobacterial seawater isolates grew on DMSP as sole carbon source and produced DMS. Furthermore, our DNA-SIP strategy also identified *Methylophaga* and other *Piscirickettsiaceae* as key bacteria likely using the DMS, generated from DMSP lysis, as a carbon source.

**Conclusions:**

This is the first study to use DNA-SIP with ^13^C-labelled DMSP and, in a novel way, it identifies the dominant microbes utilising DMSP and DMS as carbon sources. It highlights that whilst metagenomic analyses of marine environments can predict microorganisms/genes that degrade DMSP and DMS based on their abundance, it cannot disentangle those using these important organosulfur compounds for their carbon requirements. Note, the most abundant DMSP degraders, e.g. *Rhodobacterales* with DmdA, are not always the key microorganisms using DMSP for carbon and releasing DMS, which in this coastal system were *Oceanospirillales* containing DddD.

Video abstract.

**Supplementary Information:**

The online version contains supplementary material available at 10.1186/s40168-022-01304-0.

## Background

Petagrams of the sulfonium compound dimethylsulfoniopropionate (DMSP) are produced in the Earth’s oceans and marine sediment annually [1–4]. Organisms produce DMSP for its anti-stress functions, e.g. as an osmoprotectant [5], grazing deterrent [6, 7], antioxidant [8] and protectant against hydrostatic pressure [9]. In the environment, DMSP is imported by diverse bacteria and algae [10, 11] and used for its anti-stress properties, in signalling [12] or as a major source of carbon, sulfur and/or energy via DMSP catabolic pathways [13, 14]. Microbial DMSP catabolism is an important source of climate-active gases, e.g. methanethiol (MeSH) via DMSP demethylation [15] and dimethylsulfide (DMS) via DMSP lysis [16].

**Fig. 1:**
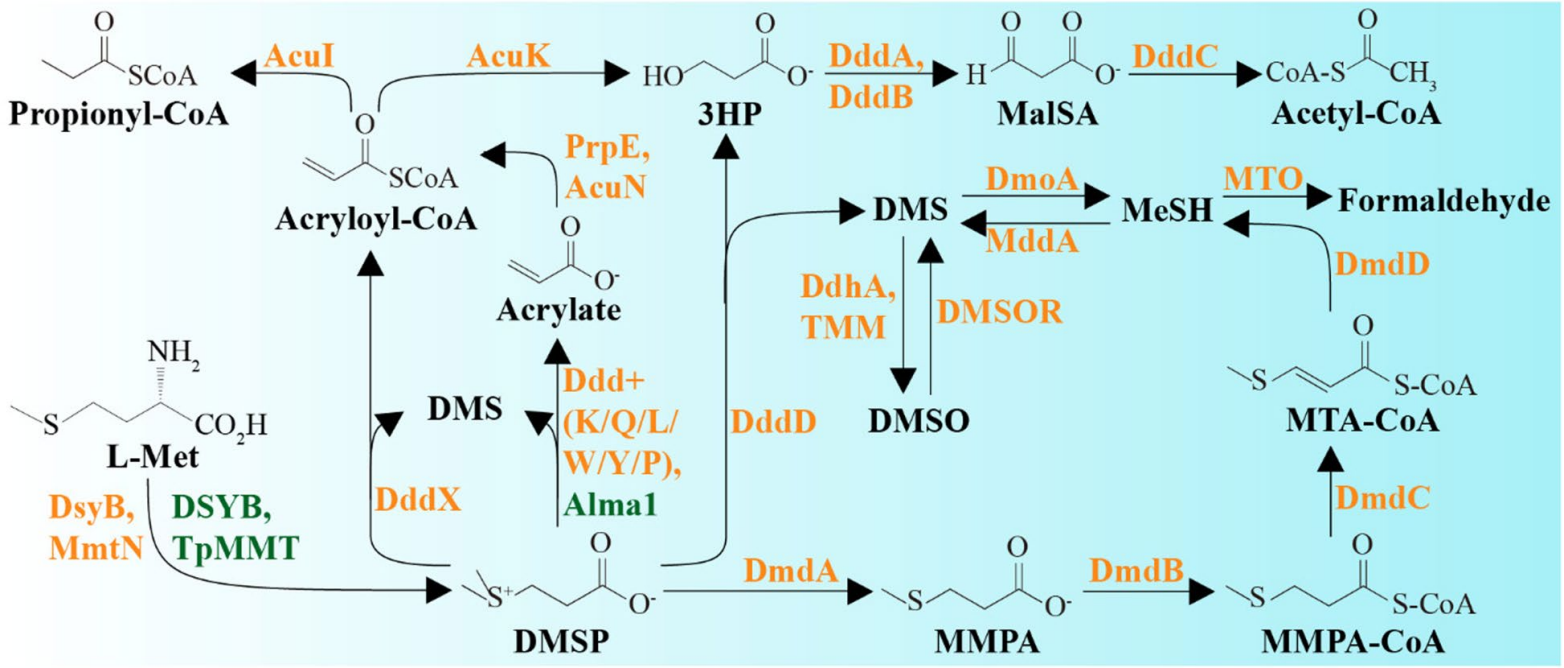
Pathways of DMSP synthesis and degradation. DMSP can be synthesised by both phytoplankton and bacterioplankton from methionine (L-Met). The SAM-dependent *S*-methyltransferase of the transamination pathway has been identified from phytoplankton (DSYB and TpMMT), and bacterioplankton (DsyB). Bacteria also can synthesise DMSP through a methylation pathway mediated by a L-Met-*S*-methylating enzyme MmtN. DMSP can be degraded through two competing pathways. The demethylation pathway involves DmdABCD and leads to the formation of methylmercaptopropionate (MMPA), methylthioacryloyl-CoA (MTA-CoA) and methanethiol (MeSH). MeSH can be oxidised to formaldehyde by MeSH oxidase (MTO). The cleavage pathway catalysed by Ddd enzymes in some bacteria, fungi and viruses, or Alma1 in algae, liberates DMS and acrylate, acryloyl-CoA or 3-hydroxypropionate (3HP). DMS can be further oxidised by marine microbes through trimethylamine monooxygenase (TMM) or dimethylsulfide dehydrogenase (DdhA) to generate dimethyl sulfoxide or by dimethylsulfide monooxgenase (DmoA) to generate MeSH. Methanethiol *S*-methyltransferase (MddA) and dimethyl sulfoxide reductase (DMSOR) mediate the production of DMS from MeSH and DMSO, respectively. Potentially toxic acrylate is detoxified by bacteria through several enzymes (PrpE, AcuI, AcuN and AcuK). Bacterial catabolism of 3HP involves DddABC proteins and generates malonate semi-aldehyde (MalSA) and acetyl-CoA. Enzymes from phytoplankton and bacteria are shown in green and orange, respectively

DMSP demethylation is initiated by the bacterial DmdA enzyme (EC 2.1.1.269, Fig. 1) that generates methylmercaptopropionate (MMPA) [17–19]. *dmdA* is widespread in marine *Alphaproteobacteria*, notably *Rhodobacterales* (also known as the *Roseobacter* group) and SAR11, and some *Gammaproteobacteria* [19]. MMPA can be further catabolised to MeSH and used as a source of carbon and/or sulfur via *dmdBCD* gene products that are common in marine and terrestrial bacteria [20]. DMSP demethylation is thought to dominate in marine systems accounting for ~75% of DMSP catabolism [21].

DMSP lyase enzymes cleave DMSP to generate DMS and either acrylate (EC 4.4.1.3), 3-hydroxypropionate (3HP; EC 3.1.2), or acryloyl-CoA as co-products [20, 22]. Eight DMSP lyases have been discovered in bacteria, fungi and viruses: DddD [22], DddL [23], DddQ [24], DddW [25], DddY [26], DddK [27], DddP [28] and DddX [29], and currently only Alma1 [30] in algae. Organisms using DMSP as a carbon source require ancillary (*ddd*, *acu* and *prp*) genes to incorporate acrylate, 3HP, or acryloyl-CoA into central metabolism (Fig. 1) [31–33]. Many bacteria, particularly the *Rhodobacterales*, possess both DMSP demethylation and cleavage pathways [34, 35].

Many metagenomics and metatranscriptomics studies examine the abundance and taxonomy of DMSP catabolic genes and their transcripts to infer the activity of DMSP-degrading microorganisms in marine environments [3, 19, 36]. However, such studies cannot elucidate with certainty the microbes and pathways used to catabolise DMSP for carbon requirements because many microbes, particularly *Rhodobacterales* with *dmdA* and/or *ddd* genes, cannot grow on DMSP as a carbon source [37] and may degrade DMSP for signalling and/or reduced sulfur requirements [12, 38]. Thus, there is a clear need to establish which microbes in marine samples use DMSP as a carbon source and what DMSP catabolic pathways they contain.

**Fig. 2:**
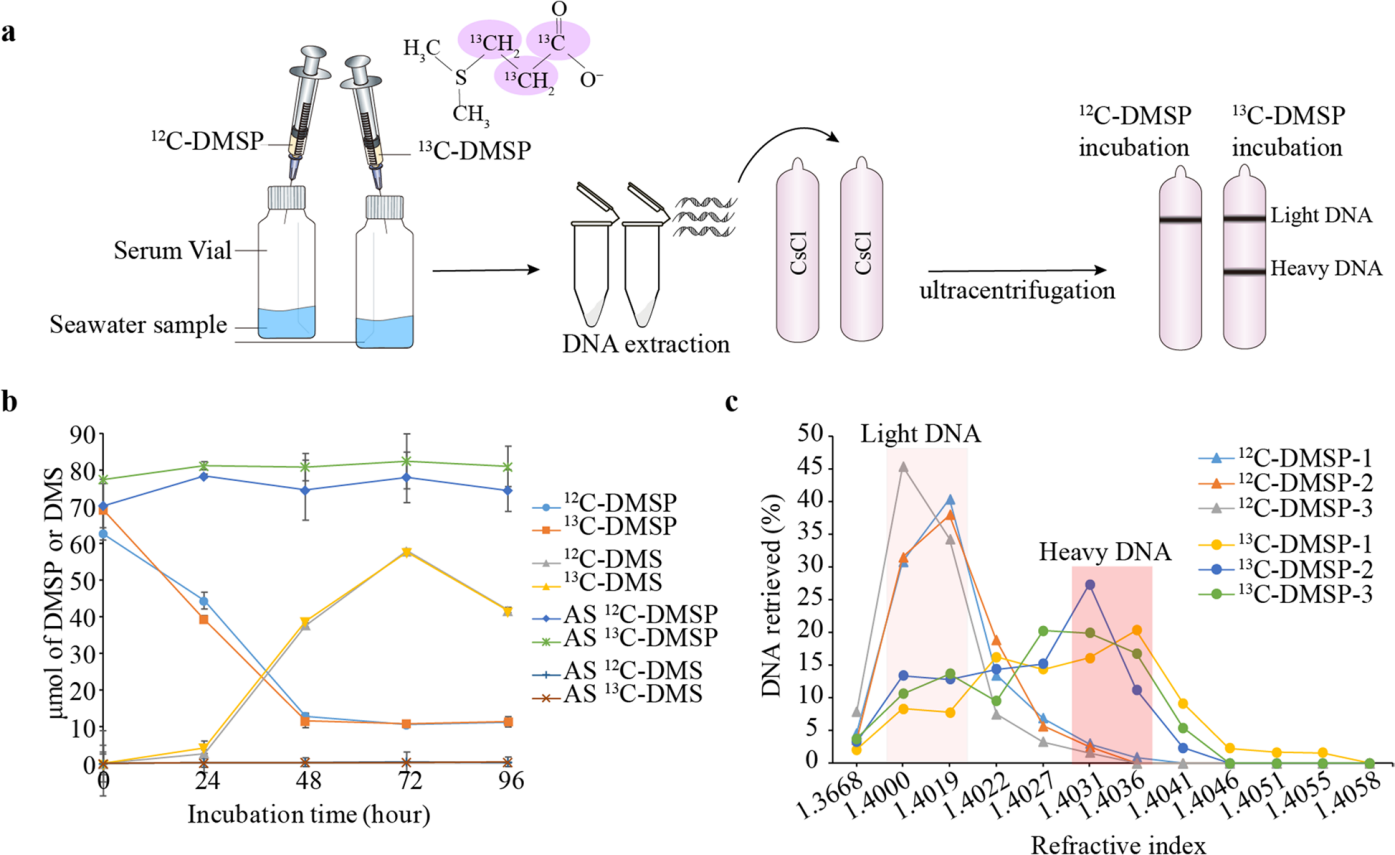
DNA-SIP experiments with ^13^C-labelled DMSP and subsequent separation of heavy and light DNA. **a** Schematic diagram of SIP experiments with ^13^C-DMSP and ^12^C-DMSP (control). **b** DMSP and DMS levels in seawater samples incubated with ^13^C- and ^12^C-DMSP. Autoclaved seawater (AS) was used as abiotic control. Values show the average of three biological replicates. **c** DNA retrieved as function of refractive index of each fraction recovered after isopycnic ultracentrifugation. Samples in shaded backgrounds were used for downstream analysis. Triangles: seawater samples incubated with ^12^C-DMSP (control). Circles: seawater samples incubated with ^13^C-labelled DMSP

Here we use ^13^C-labelled DMSP (with only the propionate moiety ^13^C-labelled; Fig. 2a) and DNA-stable isotope probing (DNA-SIP) [39, 40] combined with metagenomics and 16S rRNA gene amplicon sequencing to identify organisms in coastal seawater that utilise DMSP (enriched in ^13^C-heavy fractions) and DMS (enriched in ^13^C-light fractions) as a carbon source (Fig. 2a). Culture-dependent methods were also used to examine bacteria using DMSP as a carbon source. Thus, this study uses DNA-SIP in a novel way to provide key insights into microorganisms utilising DMSP and DMS as carbon sources in natural seawater samples and to distinguish them from microbes that might catabolise these compounds for other uses.

## Results and discussion

### Characterisation of DMSP cycling in coastal seawater

North Sea coastal water, sampled in Great Yarmouth, UK, in January 2018, contained 3.7 ± 0.4 nM DMSP and 0.9 ± 0.4 nM DMS. After addition of exogenous 100 μM DMSP, significant initial rates of DMSP removal (1.1 ± 0.1 μmol h^−1^) and DMS production (0.8 ± 0.02 μmol h^−1^) were detected over the first 48 h of incubation (Fig. 2b). Interestingly, no MeSH was detected in the incubation experiments, suggesting either that DMSP lysis dominated over the demethylation pathway in these samples or that the MeSH consumption rates were equal to the synthesis rates.

### Identification of candidate microbes cycling DMSP in natural (T0) coastal seawater

16S rRNA gene amplicon (16S) and metagenomic (MG) sequencing was used to identify microorganisms in the natural (T0) seawater samples with the potential to cycle DMSP. Analysis of both 16S and MG data revealed that the T0 microbial community was dominated by bacteria, especially *Proteobacteria* (relative abundance, RA 82.6 ± 7.3% of the total 16S reads), with archaea and eukaryotes present at very low RA (<1%; Fig. S1). Although algae, thought to be the major DMSP producers in photic seawater, were not abundant, potential DMSP-producing cryptophytes *Teleaulax* [41] and *Guillardia*, and the chlorophyte *Bathycoccus* [42] were identified in these T0 seawater samples (Fig. S2a). Furthermore, algal DMSP synthesis genes encoding proteins 100% and 86.6% identical to *Prymnesium parvum* DSYB and *Thalassiosira pseudonana* TpMMT [43, 44] were not abundant in the T0 seawater metagenomes (Fig. S2b) and were present at similar levels to bacterial *dsyB* (Table S1). This is not surprising given the DMSP synthesis genes in chlorophyte or cryptophyte algae are not known. Furthermore, few algal DMSP lyase (*Alma1*) sequences were detected compared to bacterial DMSP lysis genes suggesting that bacteria are likely the major drivers of DMSP cycling in these seawater samples; see below (Table S1; Fig. S2b−c).

**Fig. 3:**
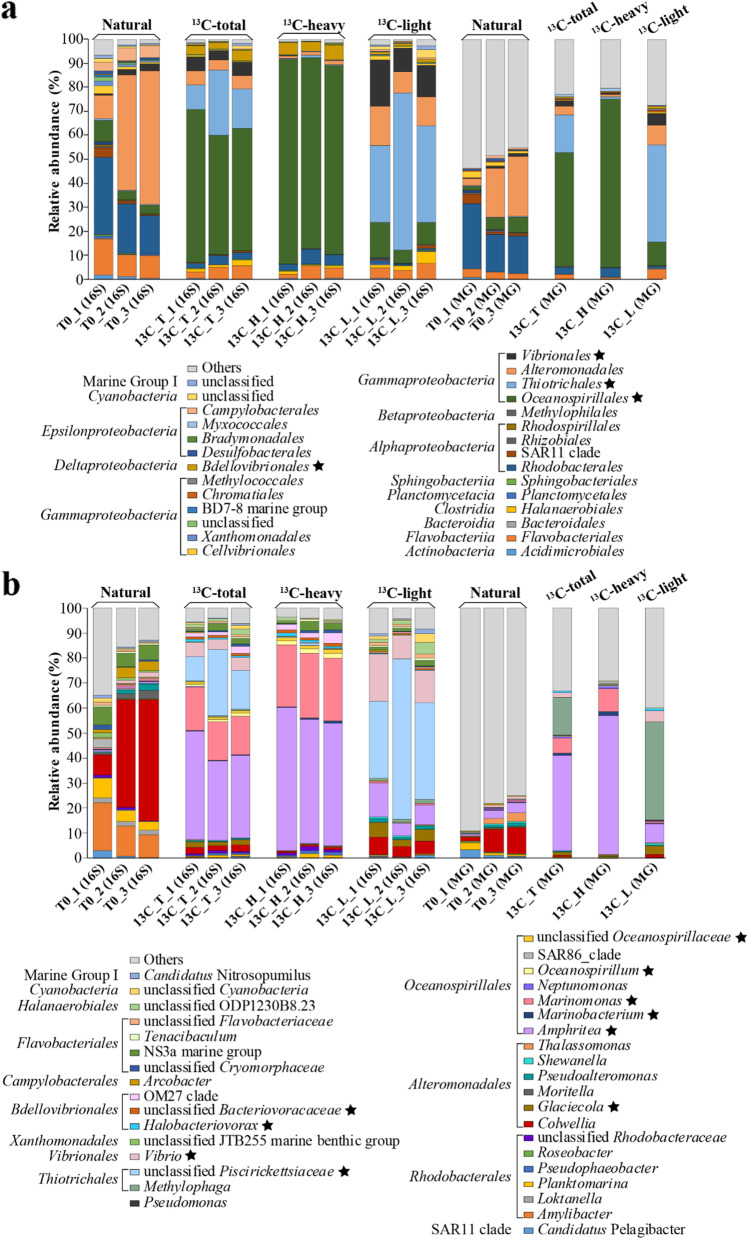
Microbial community profile of coastal seawater samples at order (**a**) and genus (**b**) levels. Bacterial diversity of the natural (T0) and labelled (heavy; H) and unlabelled (light; L) fractions of ^13^C-DMSP seawater incubations was analysed by 16S rRNA gene amplicon (16S) and metagenomics (MG) sequencing. “_1”, “_2” and “_3” after the sample name represent biological replicates. Biological replicates from ^13^C-heavy and ^13^C-light fractions were respectively combined before MG sequencing due to their highly similar 16S rRNA gene community profile shown by DGGE (Fig. S7). Only classes and genera with RA >0.5% in at least one of the conditions are represented. Statistically enriched genera in the incubations with ^13^C-labelled DMSP (13C_T) compared the natural (T0) samples based on 16S data are labelled with an asterisk. Classes and genera with RA <0.5% are grouped into “others”. For 16S and MG data of samples incubated with ^12^C-DMSP (controls), see Fig. S8

The most abundant bacteria in T0 seawater were from gammaproteobacterial (mostly *Alteromonadales*) and alphaproteobacterial (mostly *Rhodobacterales*) classes, which comprised respectively 50.2 ± 19.1% and 25.5 ± 10.9% of the 16S and 30.6 ± 13.6% and 25.1 ± 9.5% of MG data (Fig. S1 and Fig. 3). Less than 1% of the T0 microbial community was of genera predicted to produce DMSP [45, 46] and/or have DMSP synthesis genes (*dsyB* or *mmtN*), according to the 16S (Fig. S3a) and MG analysis (Fig. S3b), which was consistent with the relatively low DMSP concentrations observed in these T0 coastal waters.

Regarding DMSP catabolism, *Colwellia* strains (RA of 33.2 ± 21.9%) from the *Alteromonadales* order together with the *Rhodobacterales Amylibacter* (13.4 ± 5.1%) and *Planktomarina* (5.3 ± 2.4%) were predicted to be the major DMSP degraders in these T0 seawater samples (Fig. 3, Table S2). Indeed, marine *Colwellia* spp. isolates have previously been suggested to be important DMSP catabolisers in polar samples [47], and like several *Amylibacter* spp., they contain *dmdA* [48]. Furthermore, many *Amylibacter* spp. and *Planktomarina* spp., abundant in other coastal samples [48], contain proteins homologous to ratified DddP DMSP lyases (Table S3). However, it was surprising that *Oceanospirillales* and SAR11 clade bacteria, often abundant in surface seawater [49] and known DMSP degraders via lysis and/or demethylation pathways [17, 27], only comprised 5.3 ± 3.0% and 1.9 ± 1.8% of the T0 microbial community, respectively, according to the 16S data (Fig. 3; Table S4).

**Fig. 4:**
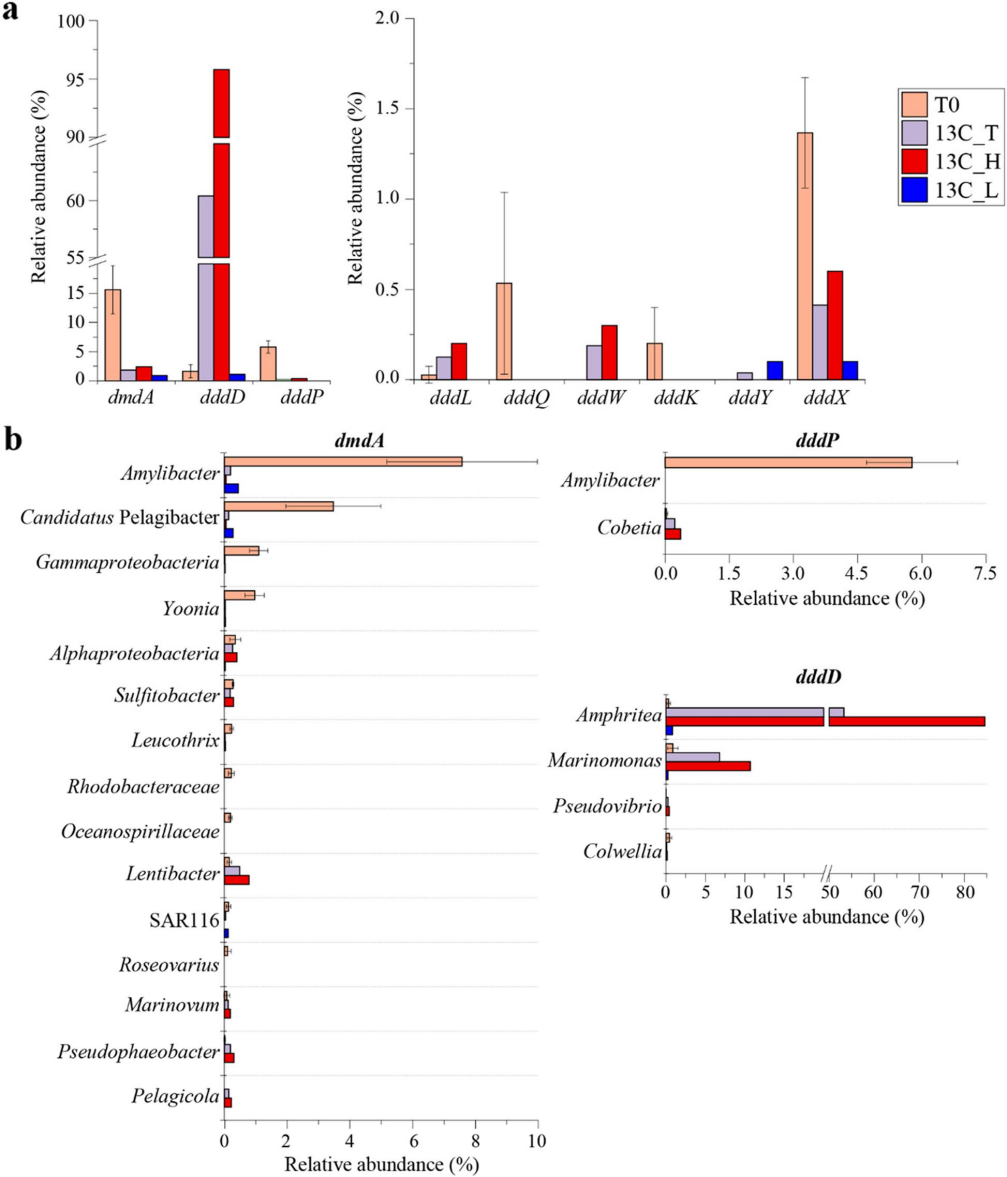
Relative abundance and taxonomic affiliation of DMSP cycling genes retrieved from coastal seawater metagenomes. **a** Relative abundance (RA) of DMSP demethylation (*dmdA*) and lysis (*ddd*) genes in seawater metagenomes. **b** Taxonomic affiliation of key genes involved in DMSP catabolism in seawater samples retrieved from metagenomic data. T0: metagenomes from natural samples; 13C_T: total microbial community from samples incubated with ^13^C-DMSP; 13C_H: metagenomes from ^13^C-heavy fractions; 13C_L: metagenomes from ^13^C-light fractions. T0 values represent the average of three biological replicates. Biological replicates from ^13^C-heavy and ^13^C-light fractions were respectively combined prior to metagenomic sequencing (see the “Methods” section)

MG analysis revealed that *dmdA* and *dddP* were the most abundant DMSP catabolic genes in the T0 samples, with 15.6 ± 4.1% and 5.8 ± 1% of bacteria predicted to contain them, respectively (Fig. 4a; Table S1). The majority of *dmdA* sequences (48.3%) were from *Rhodobacterales*, mainly homologous to *Amylibacter dmdA*, and 21.7% were from *Candidatus* Pelagibacter of the SAR11 clade (Fig. 4b and Fig. S4). Nearly all detected *dddP* sequences in T0 metagenomes (>98.8%) were also affiliated to *Amylibacter* (Fig. 4b and Fig. S5). Bacteria containing *dddD* (1.6 ± 1.1%; mainly related to gammaproteobacterial *Amphritea*, *Marinomonas* and *Colwellia* strains), *dddX* (1.4 ± 0.3%) and *dddQ* (0.5 ± 0.5%) were also relatively abundant in the T0 seawater microbial community (Fig. 4a, Fig. S6 and Table S1). Other known DMSP lyases (*dddL, dddW*, *dddK* and *dddY*) were predicted to be in only <0.5% of T0 bacteria (Fig. 4a, Fig. S6 and Table S1). These data suggest a typical coastal system where *Rhodobacterales* and SAR11 bacterial DMSP demethylation dominates over DMSP lysis through DddP and, to a lesser extent, DddD, DddX and DddQ [17, 50, 51]. However, the abundance and taxonomic profile of DMSP catabolic genes cannot predict their corresponding levels of transcription, enzyme abundance and/or activity. Furthermore, it is impossible for such an ‘omics study alone to elucidate why these microbes catabolise DMSP, e.g. to provide carbon, sulfur or signalling needs. Therefore, we conducted a DNA-SIP experiment with ^13^C-labelled DMSP to determine which microbes use DMSP as carbon source in these seawater samples.

### Identification of microorganisms degrading DMSP for use as a carbon source by DNA-SIP

Known DMSP demethylation and lysis pathways in model organisms that use DMSP as a carbon source release MeSH and DMS, respectively, as gaseous products and utilise the propionate component for carbon (Fig. 1). Thus, to identify microbes using DMSP as a carbon source, we synthesised DMSP from ^13^C_3_-acrylic acid and ^12^C-DMS (see the “Methods” section), the product hereafter being referred to as ^13^C-DMSP, which was used in our DNA-SIP experiments with ^12^C-DMSP as a control (Fig. 2a). This strategy was chosen to also enable identification of microorganisms utilising the DMS component of DMSP, i.e. those microbes enriched in incubations with DMSP but not necessarily being represented in the heavy fraction of ^13^C-DMSP incubations.

The T0 seawater samples collected above were incubated with ^13^C-DMSP or ^12^C-DMSP (control) as substrate (100 μM) under 12-h light/dark cycling conditions to consider both phototrophic and heterotrophic DMSP catabolism. No MeSH production was detected in any of the incubations. DMSP degradation, DMS production and subsequent DMS removal processes were similar between the samples incubated with ^13^C-DMSP and ^12^C-DMSP (Fig. 2b). DNA was extracted after 96 h when 77 μmol DMSP L^−1^ (231 μmol C L^−1^) was assimilated and DMS levels were decreasing (by 0.7 ± 0.006 μmol h^−1^ during the last 24 h of incubation), suggesting that DMS degraders were also active at this timepoint. DNA was then separated into heavy (^13^C-labelled) and light (^12^C-labelled) fractions by isopycnic centrifugation (see the “Methods” section; Fig. 2c), which were analysed by denaturing gradient gel electrophoresis (DGGE). This technique showed clear differences in the 16S rRNA gene profiles from ^13^C-heavy and ^13^C-light fractions, whereas fractions from ^12^C-DMSP (control) incubations had similar profiles (Fig. S7), indicating that DNA from microorganisms using ^13^C-DMSP for carbon had been successfully labelled.

Subsequently, the heavy and light DNA fractions from ^13^C-DMSP and ^12^C-DMSP (control) incubations were subjected to MG and 16S sequencing to identify those microorganisms that used DMSP as carbon source. Sequence analysis revealed that *Gammaproteobacteria* dominated in both the heavy (13C_H) and light (13C_L) fractions from ^13^C-DMSP incubations (Fig. 3a), but there were major differences at lower taxonomic levels (i.e. order and genus; Fig. 3b). *Oceanospirillales*, with a RA of 70.2% in MG and 81.2 ± 3.5% in 16S data, was the most abundant order in the ^13^C-heavy fractions (13C_H), but only represented <10% of the bacterial community from ^13^C-light fractions (13C_L; Fig. 3b, Table S4). Importantly, there was no such increase in *Oceanospirillales* RA between heavy (12C_H) and light fractions (12C_L) when the T0 samples were incubated with control ^12^C-DMSP (Fig. S8a), indicating that these *Gammaproteobacteria* were the major microorganisms assimilating carbon from the propionate moiety of DMSP in the DNA-SIP experiments.

At the genus level, *Amphritea* dominated the heavy fractions from the ^13^C-labelled microbial community (13C_H), comprising 55.9% and 52 ± 4.3% of the MG and 16S reads, respectively, followed by *Marinomonas* (9.1% RA in MG and 25.2 ± 0.5% in 16S data; Fig. 3b, Table S2). Other less abundant *Oceanospirillales* genera significantly enriched (*P* <0.05) in the ^13^C-heavy fractions compared to the T0 samples, were *Marinobacterium* (to 1.3% RA in MG and 0.6 ± 0.1% in 16S data) and *Oceanospirillum* (to 0.5% RA in MG and 1.8 ± 0.1% in 16S; Table S2). These four *Oceanospirillales* genera were 6- to 60-fold more abundant (*P* <0.05) in ^13^C-heavy (13C_H) than in the ^13^C-light (13C_L) fractions and constituted 0.4−36% of the total microbial community of the samples incubated with ^13^C-DMSP (13C_T, 16S data; Fig. 3b, Table S2). As expected, these genera were also highly abundant in both the ^12^C-light and ^12^C-heavy fractions (12C_L and 12C_H; Fig. S8b), further supporting them being key bacteria that used DMSP as a carbon source in the DMSP incubations.

In addition, two genera of the *Bdellovibrionales* order, *Halobacteriovorax* and an unclassified *Bacteriovoracaceae*, were also enriched during the ^13^C-DMSP incubations, with their RA increasing to 0.7 ± 0.2% and 1 ± 0.1% of 16S reads in the 13C_T samples from <0.1% at T0 (Fig. 3b, Table S2). These *Bdellovibrionales* bacteria were ~10-fold more abundant in the heavy (13C_H) than in the light (13C_L) fractions from incubations with ^13^C-DMSP (Table S2). No sequenced *Halobacteriovorax* strains contain known DMSP catabolic genes, so if they are true DMSP degraders they may utilise novel pathways and/or enzymes.

As only one DNA-SIP timepoint was analysed here, it is possible that some microorganisms enriched in ^13^C-heavy fractions were labelled due to cross-feeding, i.e. they assimilated ^13^C-DMSP catabolites released from primary degraders. This is unlikely for the *Oceanospirillales* given their dominance in the 13C_H fractions and that several previously studied *Oceanospirillales* strains, e.g. *Marinomonas* [22], *Oceanimonas* [52] and *Halomonas* [31], and bacterial isolates from this work, used DMSP as a sole carbon source and contained DddD (Table 1 and see below). However, it is plausible that the predatory *Bdellovibrionales* may have consumed primary DMSP degraders and become labelled due to cross-feeding [53].

**Table 1:** Characteristics of DMSP-degrading bacterial strains isolated from seawater samples incubated with DMSP

Strain	Top-hit taxon identified by 16S rRNA gene sequences	Reference strain^a^	Accession number of genomes from sequenced isolates	Homologues in reference strain or genome from sequenced isolate^b^	DMS production rate^c^	MeSH production^d^	Growth on DMSP^e^	Class
GY12	*Litoreibacter albidus*	DSM 26922		*dddA*, *dddC*, *prpE*, *acuI*, *acuK*, *dmdA*, *dmdB*, *dmdC*	ND	Y	N	*Alphaproteobacteria*
MB12-2	*Neptunicoccus sediminis*	CY02		*dddP*, *prpE*, *acuI*, *acuK*, *dmdA*, *dmdB*, *dmdC*	6 ± 2	ND	N	*Alphaproteobacteria*
GY7	*Pseudophaeobacter arcticus*	DSM 23566		*dddW*, *dddA*, *dddC*, *prpE*, *acuI*, *acuN*, *acuK*, *dmdA*, *dmdB*, *dmdC*, *dmdD*	558 ± 260	ND	N	*Alphaproteobacteria*
MB12-4	*Sulfitobacter pontiacus*	DSM 10014		*dddL*, *dmdB*, *dmdC*, *dmdD*	648 ± 153	ND	N	*Alphaproteobacteria*
GY16	*Sulfitobacter pseudonitzschiae*	H3		*dddL*, *dddA*, *dddC*, *prpE*, *acuI*, *acuK*, *dmdB*, *dmdC, dmdD*	201 ± 40	ND	N	*Alphaproteobacteria*
D12-10	*Alteromonas stellipolaris*	LMG 21861		*dmdC*	60 ± 6	ND	Y	*Gammaproteobacteria*
GY8	*Marinobacter sediminum*		JAGTWY000000000	*dddL*, *dddA*, *dddC*, *prpE*, *dmdB*, *dmdC*	4560 ± 785	ND	Y	*Gammaproteobacteria*
MC12-9	*Marinobacter similis*	A3d10		*dddL*, *dddA*, *prpE*, *acuN*, *acuK*, *dmdC*	86 ± 3	ND	Y	*Gammaproteobacteria*
GY20	*Pseudoalteromonas hodoensis*	H7			ND	Y	N	*Gammaproteobacteria*
D13-2	*Cobetia amphilecti*	KMM 296		*dddD*, *dddA*, *dddC*, *dddT*, *prpE*, *acuI*, *acuK*, *dmdC*, *tmm*	1342 ± 101	ND	N	*Gammaproteobacteria*
MC13-5	*Cobetia litoralis*		CP073342	*dddD*, *dddA*, *dddC*, *dddT*, *prpE*, *acuI*, *dmdC*, *tmm*	1316 ± 94	ND	Y	*Gammaproteobacteria*
GY6	*Amphritea atlantica*		CP073344, CP073345	*dddD*, *prpE* (2), *acuI*, *acuK*, *dddB*, *dddC*, *dddT*, *dmdB* (4), *dmdC*, *tmm*	1173 ± 208	ND	Y	*Gammaproteobacteria*
D13-1	*Marinobacterium rhizophilum*		CP073347	*dddD*, *dddP*, *prpE* (2), *acuI*, *acuN*, *acuK*, *dddA*, *dddB*, *dddC*, *dddT*, *dmdB* (2), *dmdC*	1039 ± 21	ND	Y	*Gammaproteobacteria*
MC13-7	*Marinobacterium profundum*	PAMC 27536		*dddD*, *dddP*, *dddA*, *dddB*, *dddC*, *dddT*, *prpE* (2), *acuI*, *dmdB*, *dmdC*	1132 ± 31	ND	Y	*Gammaproteobacteria*
GY1	*Marinomonas atlantica*	Cmf 18.22		*dddD*, *dddB*, *dddC*, *prpE*, *acuI*, *dmdC*	59 ± 30	ND	Y	*Gammaproteobacteria*
MB12-3	*Marinomonas foliarum*	CECT 7731		*dddC*, *acuI*, *dmdC*	11 ± 0.4	ND	N	*Gammaproteobacteria*
MB12-11	*Marinomonas rhizomae*		CP073343	*dddD*, *dddB*, *dddC*, *dddT*, *acuI*, *dmdC*	500 ± 36	ND	Y	*Gammaproteobacteria*
D13-4	*Pseudomonas benzenivorans*		CP073346	*dddD*, *dddP*, *acuK*, *dddB*, *dddC*, *dddT*, *dmdB*, *dmdC*	2462 ± 123	ND	Y	*Gammaproteobacteria*
GY22	*Pseudomonas leptonychotis*	CCM 8849		*dddD*, *dddB*, *dddC*, *dddT*, *dmdB*, *dmdC*	2350 ± 343	ND	Y	*Gammaproteobacteria*
GY17	*Pseudomonas taeanensis*	MS-3		*dddD*, *dddP*, *dddB*, *dddC*, *dddT*, *acuI*, *dmdB*, *dmdC*, *tmm*	829 ± 186	ND	Y	*Gammaproteobacteria*
GY15	*Vibrio splendidus*	10N.286.45		*prpE*, *acuI*, *DMSOR*	ND	Y	Y	*Gammaproteobacteria*

^a^Reference strain: most closely related strain with publicly available genome

^b^Number of *prpE* and *dmdB* genes in genomes with multiple copies are indicated in brackets

^c^Rate of DMSP-dependent DMS production expressed in nmol DMS mg protein^−1^ h^−1^

^d^Y, detectable MeSH production from DMSP. ND, not detected

^e^Y, growth on DMSP as sole carbon source (Student’s *t*-test, *P* < 0.05); N, no growth on DMSP as sole carbon source (*P* > 0.05)

Interestingly, *Gammaproteobacteria*, including members of the orders *Alteromonadales* (*Glaciecola*), *Vibrionales* (*Vibrio*) and, most notably, *Thiotrichales* (unclassified *Piscirickettsiaceae*), were also enriched during the incubations with ^13^C-labelled DMSP (RA 0.003−0.2% in T0 to 1.8−17.3% in 13C_T samples according to 16S data; Table S2). These *Gammaproteobacteria* were more abundant in the ^13^C-light than in the ^13^C-heavy fractions (RA >15-fold higher in 13C_L than in 13C_H fractions; Fig. 3b, Table S2), suggesting that they likely assimilated the ^12^C-DMS generated from the lysis of ^13^C-DMSP during the incubations with ^13^C-DMSP (see below).

Strikingly, *Rhodobacterales* and SAR11 bacteria were >6-fold less abundant after the ^13^C-DMSP incubations (13C_T) compared to the T0 samples (16S data, Fig. 3a, Table S4). Although some *Rhodobacterales* 16S rRNA genes, notably from *Pseudophaeobacter* and *Planktomarina*, were mainly present in the ^13^C-heavy fractions consistent with them using DMSP as a carbon source, these bacteria comprised only 1% of the ^13^C-labelled bacterial community (13C_H; Fig. 3b, Table S2). Furthermore, SAR11 bacteria represented 0.3% of the total microbial community from ^13^C-DMSP incubations (13C_T) and were >10-fold less abundant in the heavy (13C_H) than light (13C_L) fractions (Fig. 3b, Table S4). These data imply that *Rhodobacterales* and SAR11 bacteria, which mostly possess *dmdA* [15, 17] and at least one *ddd* gene [37, 54], were not the major users of DMSP as a carbon source under these incubation conditions. It is possible that these bacteria, predicted to be key DMSP degraders in coastal waters [50, 55], were using DMSP at the low T0 nM concentration for sulfur demands [56], and/or to generate DMS and/or acrylate as signalling molecules [12] and not primarily as a carbon source. Indeed, most *Rhodobacterales* with *dmdA*/*ddd* genes do not typically grow well on DMSP as sole carbon source under lab conditions [37], in comparison to *Oceanospirillales* bacteria with *dddD* (see below) [37] or *Alcaligenes faecalis* with *dddY* [26].

### Abundance and taxonomy of DMSP catabolic genes in seawater samples incubated with ^13^C-DMSP

After incubation with ^13^C-DMSP, DMSP demethylation was no longer predicted to be the dominant DMSP catabolic pathway it was in the T0 seawater samples. Metagenomic analysis showed that the RA of *dmdA* in T0 seawater metagenomes (15.6 ± 4.1% of bacteria) decreased 10-fold to 1.8% after incubation with ^13^C-DMSP (13C_T; Fig. 4a and Table S1). Of the *dmdA* sequences in these samples, 73% originated from the ^13^C-heavy fractions and were mainly closely related to DmdA from *Rhodobacterales*, e.g. *Lentibacter* (RA 0.8%; Fig. 4b). These data indicate that some bacteria may have demethylated DMSP for carbon assimilation during the incubations with ^13^C-DMSP, but that this process was far less significant than in the natural (T0) seawater samples. In support of this, the RA of the ancillary *dmdCD* genes (Fig. 1) also decreased during the incubations with ^13^C-DMSP (13C_T) compared to the T0 samples (Fig. S9). Conversely, *dmdB* sequences showed a higher RA in the 13C_T than in the natural (T0) metagenomes and were mainly affiliated to *Amphritea* bacteria (Fig. S9), which were highly enriched in the ^13^C-heavy fractions (13C_H; Fig 3b), have multiple copies of *dmdB* but not *dmdA* and are unlikely to demethylate DMSP (Table 1).

**Fig. 5:**
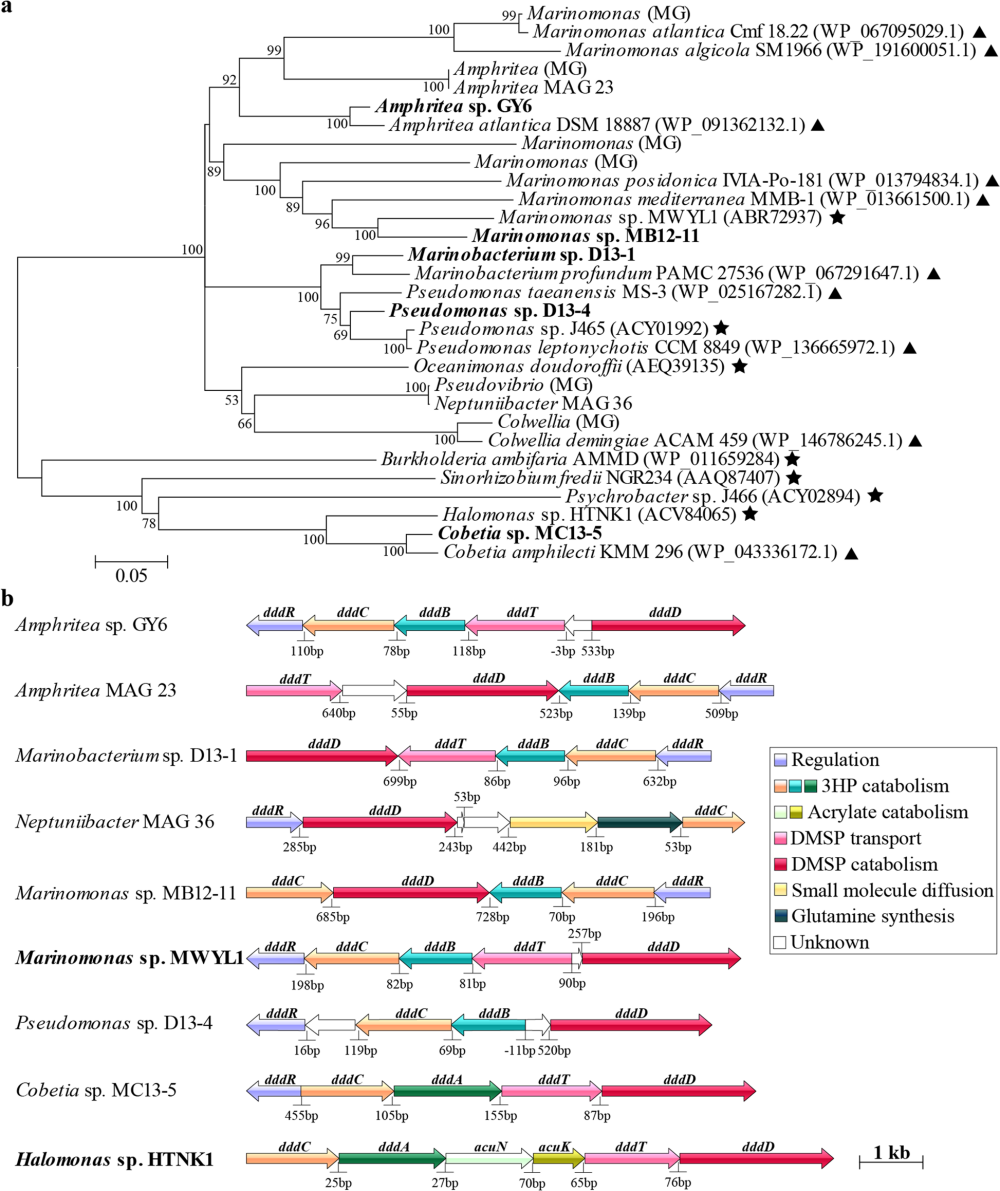
Maximum likelihood phylogenetic tree of DddD proteins (**a**) and DMSP-catabolising gene clusters containing *dddD* from bacterial isolates and metagenome-assembled genomes (MAGs) retrieved from this study (**b**). **a** The tree shows DddD proteins from strains isolated in this study (in bold), previously ratified proteins (★), together with sequences retrieved from seawater metagenomes (MG), metagenome-assembled genomes (MAG), and reference strains (▲). Reference strains were selected as the most closely related strains to isolates from this study having publicly available genome sequences. Bootstrap values ≥50% (based on 100 replicates) are shown. Scale bar indicates 5% estimated phylogenetic divergence. **b** Gene function is indicated by a colour code detailed in legend. Bacteria in bold are those in which the gene clusters have been experimentally ratified

In contrast to *dmdA*, the RA of *ddd* genes increased to ~60% after the incubation with ^13^C-DMSP (13C_T samples), most of which (97%) were retrieved from the ^13^C-heavy fraction metagenomes (13C_H; Fig. 4a). Among the *ddd* genes, *dddD* was particularly enriched in the ^13^C-heavy fraction, with 95.8% of bacteria predicted to contain it (Fig. 4a and Table S1). Most of the *dddD* sequences were closely related to the *Oceanispirillales* genera *Amphritea* (84.5%) and *Marinomonas* (10.7%; Figs. 4b and 5a), supporting the 16S and MG data, which showed that these were the dominant genera in the ^13^C-heavy fraction (13C_H; Fig. 3, Table S2). *dddD* genes likely from *Colwellia* and *Pseudovibrio* were also retrieved from the ^13^C-heavy metagenomes, although with lower RA (<0.5%; Figs. 4b and 5a). Therefore, the DNA-SIP experiment with ^13^C-labelled DMSP clearly showed that *Oceanospirillales* were the key bacteria degrading DMSP for carbon via a DddD-mediated lysis pathway in these coastal seawater samples. This is not surprising since it has been previously reported that many *Oceanospirillales* bacteria with DddD, an enzyme with a high affinity for DMSP [57], can use DMSP as sole carbon source for growth [22, 31, 50].

Four other DMSP lyase genes (*dddP*, *dddL*, *dddW*, *dddX*) were also detected in the metagenomes from the ^13^C-heavy fraction, but were much less abundant than *dddD* (RA of 0.2−0.6%; Fig. 4 and Table S1). These DMSP lyase genes were taxonomically affiliated to the *Oceanospirillales* genus *Marinobacterium* (*dddX*), and *Rhodobacteraceae* genera *Sulfitobacter* (*dddL*), *Phaeobacter* and *Pseudophaeobacter* (*dddW*; Fig. S6). Interestingly, the *dddP* sequences retrieved from the ^13^C-heavy fraction (13C_H) were closely related to *Cobetia* (*Oceanospirillales*) *dddP*, but no *dddP* genes from *Rhodobacteraceae* bacteria, including *Amylibacter*, whose *dddP* genes represented >98.8% of the *dddP* sequences in the T0 metagenomes, were detected (Fig. 4b and Fig. S5). These data imply that bacteria containing *dddL*, *dddW*, *dddX* and, notably, *dddP*, the most abundant DMSP lyase gene in marine environments [43], play a far less significant role in the assimilation of carbon from DMSP compared to the *Oceanospirillales* harbouring *dddD*, but may have important other roles in, e.g. generating DMS or acrylate as info-chemicals [12].

A complete suite of ancillary genes involved in the downstream catabolism of DMSP (*prpE*, *acuI*, *dddB*, *dddC* and *dddT*) [31–33] was enriched in the samples incubated with ^13^C-DMSP (13C_T) compared to the T0 samples (Fig. S10 and Table S1). These genes mostly originated from the ^13^C-heavy fractions (13C_H) and were affiliated to the *Oceanospirillaceae* genera *Amphritea* and *Marinomonas* (Fig. S10). Interestingly, *dddA*, whose product catalyses the same reaction as DddB in *Halomonas* [31], was present at a much lower RA in the ^13^C-DMSP incubations (13C_T) compared to the natural (T0) seawater samples, and it was much less abundant in the ^13^C-heavy fractions (13C_H) than *dddB* (4.6% vs 90.9%; Table S1). Thus, DddB likely represents the major route of malonate semialdehyde formation from DMSP in the seawater samples amended with ^13^C-DMSP (Fig. 1). Similarly, MG analysis revealed that *Oceanospirillaceae* bacteria assimilating carbon from ^13^C-DMSP in our incubations most likely metabolise acrylate via the PrpE-AcuI enzymes, rather than through AcuN-AcuK (Fig. 1). Although these four genes were mostly present in the ^13^C-heavy fractions (13C_H), the RA of *acuN* and *acuK* in the total microbial community (13C_T) from the ^13^C-DMSP incubations (1.8% and 21.9%, respectively) was lower than in the T0 samples (8.3 ± 1.7% and 34 ± 5.8%; Fig. S10 and Table S1). The opposite trend was observed with *prpE* and *acuI*, which had a 4- and 2-fold higher RA in the incubations with ^13^C-DMSP (13C_T) compared to the natural (T0) samples (Fig. S10 and Table S1).

### Insights into DMSP cycling from metagenome-assembled genomes (MAGs)

To gain further insights into the microbial sulfur cycling pathways in this coastal environment, metagenome-assembled genomes (MAGs) were reconstructed and analysed. MAGs were screened for the presence of DMSP degradation genes and their RA in each sample calculated as described in Methods. *dmdA*, the dominant DMSP catabolic gene in T0 samples, was found in the most abundant MAG (5.1 ± 2.1% RA) from the T0 metagenomes (MAG 55, *Alphaproteobacteria*; Table S5) and also in several less abundant (RA <0.5% each) gammaproteobacterial (MAGs 11, 58 and 60) and *Rhodobacterales* MAGs (MAGs 3, 7, 18, 44, 68, 81; Fig. S4 and Table S5). MAG 44, taxonomically classified as *Loktanella*, also harboured a sequence encoding a DddW-like protein with 54% amino acid identity to *R. pomeroyi* DddW, which did not cluster with ratified DddW sequences (Fig. S6). Most of the *dmdA*-containing MAGs (except for MAGs 55, 58 and 68) also had *dddP* and were mainly classified as *Rhodobacterales* (Table S5). MAGs 55, 58 and 68 were only predicted to be 55.2 to 75.1% complete (Table S5), which might account for why no *dddP* homologues were detected. The *Rhodobacterales* MAGs whose taxonomy could be resolved at lower levels, belonged to the genera *Loktanella*, *Lentibacter* and *Amylibacter* (Table S5), which is consistent with these bacteria often containing both DMSP demethylation and lyase genes (Table S3). *dddP* was also found in a *Planktomarina temperata* MAG (MAG 73) that comprised 2.8 ± 1.9% of the T0 microbial community, and two gammaproteobacterial MAGs (<0.3% RA; Table S5 and Fig. S5). Most of the MAGs containing *dmdA* and/or *dddP* genes were not enriched after the ^13^C-DMSP incubations (13C_T) compared to the T0 samples except for the *Rhodobacterales* genera *Lentibacter* (MAG 81) and *Pseudophaeobacter* (MAG 68) and gammaproteobacterial *Neptuniibacter* MAGs (MAG 36, also containing *dddD*), which were mainly present in the ^13^C-heavy fraction (>17-fold higher RA than in ^13^C-light fraction; Table S5). These data further support the hypothesis that most *Rhodobacterales* and *Gammaproteobacteria* with DmdA and/or DddP are important DMSP degraders in coastal waters but not primarily for carbon assimilation.

Consistent with the 16S data, the RA of an *Amphritea* MAG harbouring *dddD* (MAG 23) dramatically increased in the samples incubated with ^13^C-DMSP (from 0.7 ± 0.5% in T0 to 35.3% in 13C_T samples) and was 12-fold more enriched in the ^13^C-heavy (13C_H) than ^13^C-light fractions (13C_L; Table S5). Prior to this study, *Amphritea* had not been shown to catabolise DMSP, perhaps because many *Amphritea* spp. lack *dddD* or other known DMSP-lyase genes (Table S3).

Another two *Oceanospirillales* MAGs (MAGs 30 and 85) enriched in the ^13^C-heavy fraction (13C_H) after incubation with ^13^C-DMSP (26-fold and 3-fold more abundant than in the ^13^C-light fraction 13C_L, respectively) were detected (Table S5). However, neither of these MAGs were assigned to *Marinomonas*, shown to be abundant in the 16S analysis described above, nor did they contain a known DMSP catabolic gene, again potentially due to their low completeness (61.4% for MAG 30 and 51.7% for MAG 85; Table S5).

As described above, *Halobacteriovorax* bacteria were enriched during incubation with^13^C-DMSP and one *Halobacteriovorax* MAG (MAG 43) was resolved from the metagenomic data. However, MAG 43 was more abundant in the ^13^C-light (13C_L) than in the ^13^C-heavy (13C_H) fractions after incubation and it lacked known DMSP catabolic genes (84.5% completeness; Table S5). Further studies are needed to evaluate if and how marine *Halobacteriovorax* catabolise DMSP.

### Characterisation of bacterial strains isolated from seawater samples after incubation with DMSP

Sixty-six bacterial strains with distinct morphologies were isolated after incubations of T0 seawater with DMSP, 67% of which were able to catabolise DMSP yielding DMS or MeSH. All the DMSP-degrading isolates were either *Alpha-* (27%) or *Gammaproteobacteria* (73%; Table S6). Twenty-one representative strains of the different colony types were selected (Table 1), and publicly available genomes of closely related strains (herein described as reference strains) were analysed for the presence of *dmd*/*ddd* genes. In addition, we sequenced the genome of six isolates with high DMSP-dependent DMS production rates, but where no closely related reference strains were available, or where these did not contain *dmd/ddd* homologues (Table S6).

All DMSP-degrading alphaproteobacterial isolates were *Rhodobacteraceae*, well known for their ability to catabolise DMSP [23, 58]. The *Sulfitobacter*, *Neptunicoccus* and *Pseudophaeobacter* isolates, whose corresponding reference strains contained *dddP*, *dddL* and/or *dddW* DMSP lyase genes, released DMS from DMSP, with production rates ranging from 6 to 1411 nmol DMS·mg protein^−1^·h^−1^ (Table 1 and Table S6). Most *Rhodobacterales*, including all reference strains analysed here except for *Sulfitobacter*, contained *dmdA* and were predicted to demethylate DMSP [17]. However, only one isolate, *Litoreibacter* sp. GY12, with no DMSP lyase activity, showed MeSH production from DMSP. In addition, of the twelve *Rhodobacterales* isolates, only two strains MB13-6 and MC12-6 grew on DMSP as sole carbon source (Table S6). Thus, these data further support the hypothesis that most *Rhodobacterales* do not catabolise DMSP for carbon assimilation but likely use it as a sulfur source, in oxidative stress protection [8] or for signalling processes [12].

Of the DMSP-degrading gammaproteobacterial isolates, 43% were *Oceanospirillales* from the genera *Cobetia*, *Amphritea*, *Marinobacterium* and *Marinomonas*, all of which had relatively high DMSP lyase activity (Table S6). Indeed, *Amphritea* and *Marinomonas* were identified by the DNA-SIP experiments above, as being the two most dominant degraders of DMSP for their carbon demands (Fig. 3b and Table S2). Furthermore, 63% of the *Oceanospirillales* isolates grew on DMSP as sole carbon source (Table S6), with their reference strains mostly containing *dddD* genes (Table 1, Table S6 and Fig. 5). These data support the major finding from the DNA-SIP work that *Oceanospirillales* bacteria were the key DMSP degraders for carbon assimilation in these coastal waters.

Other gammaproteobacterial strains from the *Alteromonadales* (*Alteromonas*, *Marinobacter* and *Pseudoalteromonas*), *Pseudomonadales* (*Pseudomonas*) and *Vibrionales* (*Vibrio*) orders were also isolated from the seawater samples incubated with DMSP (Table S6). *Alteromonas* and *Marinobacter* strains had DMSP lyase activity and could use DMSP as sole carbon source, whereas *Pseudoalteromonas* sp. GY20 generated MeSH from DMSP but could not use DMSP as a carbon source. Although the genomes of the *Marinobacter* isolate and its reference strain harboured *dddL*, no DMSP catabolic genes were found in any genomes of the *Alteromonas* or *Pseudoalteromonas* reference strains (Table 1). All six *Pseudomonas* spp. isolates had DMSP lyase activity and grew on DMSP as sole carbon source (consistent with previous work [59]), except for isolate MC12-18 (Table S6). In addition, all the genomes of the *Pseudomonas* reference strains and isolates analysed contained *dddD* (Table 1). Finally, *Vibrio* sp. GY15 produced MeSH from DMSP and could use the latter as sole carbon source (Table 1). To our knowledge, no *Vibrio* spp. have been shown to contain *dmdA*. Thus, it will be interesting to further investigate the DMSP catabolic mechanisms in the *Gammaproteobacteria* that cleave and/or demethylate DMSP but lack the known *ddd* and *dmdA* genes in their genomes. Given the low RA of *Alteromonadales*, *Pseudomonadales* and *Vibrionales* bacteria (<1.3% in 16S and <1.1% in MG data; Fig. 3, Table S4) in the ^13^C-heavy fractions (13C_H) after incubation with ^13^C-DMSP, they are unlikely to be as important catabolisers of DMSP for carbon assimilation as the *Oceanospirillales* in the coastal samples studied here, but they may be in other environments.

**Fig. 6:**
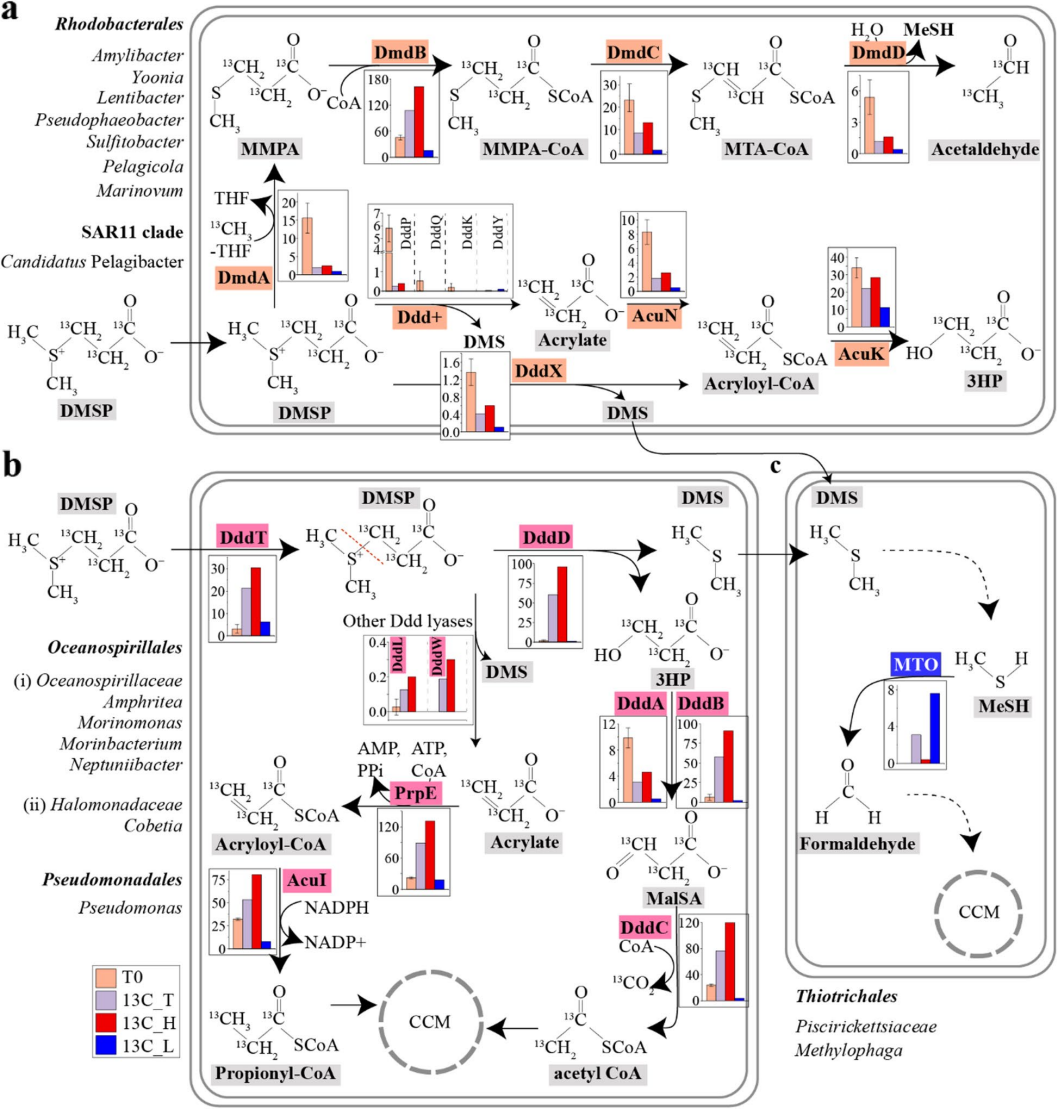
DMSP degradation pathways in microorganisms from coastal seawater samples. **a** Although *Rhodobacterales* (*Roseobacter* group) and SAR11 dominated the bacterial community of the natural (T0) seawater samples and their DMSP demethylation and cleavage genes (*dmdA* and *dddP* mainly) were relatively abundant in the T0 metagenomes (orange bars), the majority of the *Roseobacter* isolates were not able to grow on DMSP as sole carbon source. Thus, *Rhodobacterales* and SAR11 are predicted to use DMSP predominantly as a source of reduced sulfur and/or signalling in this coastal seawater. **b ***Oceanospirillales* were the major bacteria degrading DMSP for carbon requirements in the seawater incubations with ^13^C-DMSP. *dddD* from *Oceanospirillales* was the most abundant DMSP lyase gene in heavy fractions from samples incubated with ^13^C-DMSP (13C_H; red bars), although other DMSP lyase genes from *Roseobacters*, i.e. *dddL* and *dddX* were also present. Genes involved in the downstream catabolism of 3HP (*dddBC* in *Oceanospirillaceae* and *dddAC* in the *Halomonadaceae*) and acrylate (*prpE* and *acuI*) were also enriched in the 13C_H metagenomes compared to those from T0 samples. **c** DNA-SIP experiments showed that *Methylophaga*, a genus of the *Piscirickettsiaceae* family, and its gene encoding methanethiol oxidase (MTO) were highly abundant in the metagenomes from the ^13^C-light fraction (13C_L; blue bars), indicating that these bacteria were the major degraders of the DMS generated from the lysis of DMSP by *Oceanospirillales*. Bar charts represent the relative abundance of key genes involved in DMSP catabolism in metagenomes from natural (T0) seawater samples, ^13^C-DMSP incubations (13C_T) and heavy (labelled, 13C_H) and light (unlabelled, 13C_L) fractions from incubations with ^13^C- DMSP. T0 data show the average of three biological replicates, whereas replicates from ^13^C-heavy and ^13^C-light fractions were pooled prior to metagenomics analysis (see the “Methods” section). MMPA, methylmercaptopropionate; MTA-CoA, methylthioacryloyl-CoA; 3HP, 3-hydroxypropionate; MalSA, malonate semi-aldehyde; MeSH, methanethiol; CCM, central carbon metabolism

The *dddD* genes in the genomes and MAGs from *Oceanospirillaceae* bacteria (*Amphritea*, *Marinobacterium*, *Marinomonas* and *Neptuniibacter*) and *Pseudomonadales* (*Pseudomonas*) were always linked to ancillary genes involved in downstream DMSP catabolism (Figs. 5b and 6), showing similar gene synteny (*dddBCDRT*) to *dddD* from *Marinomonas* sp. MWYL1, which assimilates carbon from DMSP for growth [22]. This was also the case for *Halomonadaceae* bacteria with DddD, including *Halomonas* sp. HTNK1 and *Cobetia* sp. MC13-5 but with *dddA* replacing *dddB* [31]. Such linkage of DMSP lyase genes to their ancillary metabolic and transport genes, and their coordinated gene expression, is likely important in allowing bacteria harbouring them to utilise DMSP as carbon source.

### The role of other bacterial groups in DMS and MeSH cycling

The DNA-SIP strategy used in this study potentially allowed the identification of microorganisms assimilating the ^12^C-DMS component of ^13^C-DMSP, which should be preferentially represented in the ^13^C-light fractions (13C_L) from the incubations with ^13^C-labelled DMSP.

Sequencing data revealed that the RA of *Thiotrichales* from the ^13^C-DMSP incubations (13C_T) increased both in the 16S (from 0.5 ± 0.3% to 17.8 ± 8.6%) and MG (from 0.4 ± 0.1% to 15.6%) analyses compared to the T0 samples (Fig. 3a, Table S4). Furthermore, sequences from this order were >58-fold more abundant in the ^13^C-light (13C_L) than in the ^13^C-heavy (13C_H) fractions (Fig. 3a, Table S4). Such differences were not observed between light (12C_L) and heavy (12C_H) fractions of control samples incubated with ^12^C-DMSP (Fig. S8a). Analysis of 16S data showed that these *Thiotrichales* bacteria comprised *Methylophaga* and other unclassified genera from the *Piscirickettsiaceae* family which, based on the higher phylogenetic resolution of the MG analysis, may also be *Methylophaga* spp. (Fig. 3b). Similarly, the RA of gammaproteobacterial *Vibrio* and *Glaciecola* strains were respectively 5- and 9-fold more abundant after the incubations with ^13^C-DMSP (13C_T) than in the natural (T0) seawater samples and were mainly present in the ^13^C-light fraction (137- and 15-fold higher RA than in the ^13^C-heavy fraction; Fig. 3b, Table S2). These data imply that these *Thiotrichales* and *Gammaproteobacteria* can use the ^12^C-DMS generated from the lysis of the ^13^C-DMSP by *Oceanospirillales* bacteria with DddD for carbon assimilation. This is further supported by previous studies that have shown that several *Methylophaga*, *Vibrio* and *Glaciecola* strains catabolise DMS [60–63]. Indeed, the marine methylotroph *M. thiooxydans*, which was isolated from a DMS enrichment experiment and can use this compound as sole carbon and energy source for growth [64], is considered a model microorganism to study DMS degradation.

To investigate the underlying genetic mechanisms of DMS cycling, the MG data were interrogated with ratified genes involved in DMS metabolism (Fig. 1). Of these, *tmm*, whose product can generate dimethyl sulfoxide (DMSO) from DMS [65], was the most abundant DMS cycling gene in the T0 seawater samples (9.9 ± 1.7% RA) followed by *DMSOR* (5.3 ± 3.4% RA; Fig. S11a and Table S1), which encodes a DMSO reductase enzyme that catalyses the reverse reaction ([64], Fig. 1). Other genes involved in DMS metabolism such as *mddA* [66], *ddhA* [67] and *dmoA* [68] were present in <0.7% of the bacteria from the natural T0 samples (Fig. S11a and Table S1), suggesting that they have less important roles in this coastal seawater. *mtoX*, encoding MeSH oxidase [69], was the only known gene involved in DMS metabolism that was enriched in the incubations with ^13^C-DMSP (13C_T; 3.1% RA) compared to T0 samples (0.003 ± 0.006% RA), with the majority of sequences being from the ^13^C-light fractions (19-fold higher RA in 13C_L than 13C_H DNA; Table S1). As expected, all the *mtoX* sequences retrieved from the ^13^C-light fractions were closely related to DMS-catabolising *Methylophaga* spp (Fig. S11b). In addition, a *Piscirickettsiaceae* MAG (MAG 21) containing an *mtoX* sequence (Table S5) was more abundant in the ^13^C-DMSP samples (13C_T; RA 1.5%) compared to the natural (T0) seawater (RA 0.01 ± 0.004%) and was 34-fold more enriched in the ^13^C-light (13C_L) than in the ^13^C-heavy fraction (13C_H; Table S5), further supporting the notion that members of this family are key bacteria cycling DMS in this coastal seawater. These data are also consistent with previous work showing that *mtoX*, whose transcription and protein expression is upregulated by growth with DMS in *Methylophaga thiooxydans*, is the only known reporter gene for carbon assimilation from DMS in the *Thiotrichales* [70]. In *M*. *thiooxydans* and likely other microorganisms that use DMS as carbon source, DMS is proposed to be initially demethylated to MeSH [67]. However, the *dmoA* gene, whose product converts DMS into MeSH, was not enriched in the ^13^C-DMSP incubations (13C_T) compared to T0 samples nor was it present in the *Piscirickettsiaceae* MAG or many of the *Methylophaga* available genomes (Table S1 and Table S5). Therefore, it is possible that the initial generation of MeSH from DMS in *Methylophaga* might be catalysed by a novel enzyme and that it rapidly enters central metabolism via a reaction mediated by MTO (MeSH oxidase) [68].

## Conclusions

This study represents the first attempt to identify, in tandem, distinct microorganisms degrading DMSP and/or DMS for carbon assimilation using DNA-SIP with ^13^C-labelled DMSP.

Given that *Rhodobacterales* and to a lesser extent SAR11 with *dmdA* and *dddP* genes were highly abundant in the coastal seawater studied here, and that representative strains of these bacteria can grow on DMSP as sole carbon source [28, 71], one would predict that they would play a major role in degrading DMSP for their carbon demands. However, DNA-SIP experiments showed that the less abundant *Oceanospirillales* bacteria in the natural (T0) samples were likely the key degraders of DMSP for carbon assimilation via DddD-mediated DMSP lysis. *Oceanospirillales* bacteria, e.g. the novel and dominant DMSP degraders in the DNA-SIP experiments, *Amphritea* and *Marinomonas*, were predicted to cleave DMSP into DMS and 3HP via their DddD enzyme, with the 3HP being assimilated and the DMS released for the benefit of a different suite of microorganisms (Fig. 6). Sequencing analysis also revealed that *Rhodobacterales* and SAR11 and their *dmdA* and *dddP* genes, though abundant in most surface waters, were scarcely present after incubation with ^13^C-DMSP, suggesting that these important bacteria likely utilise DMSP demethylation and DddP-mediated DMSP lysis either for their reduced sulfur requirements [56], to protect against oxidative stress [8] or to generate signalling molecules [12, 72]. These conclusions from SIP experiments with ^13^C-labelled DMSP were supported by culture-dependent work since (i) most *Oceanospirillales* isolates, e.g. *Amphritea*, yielded DMS from DMSP; (ii) these bacteria could use DMSP as a carbon source and in nearly all cases possessed DddD; and (iii) most *Rhodobacterales* isolates contained *dmdA* and could cleave DMSP but were not able to use it as a carbon source. However, it should be noted that our DNA-SIP experiments used artificially high (100 μM) levels of ^13^C-DMSP, which may have favoured the growth of, and carbon assimilation by, *Oceanospirillales* via DMSP cleavage rather than SAR11 and *Rhodobacterales* via DMSP lysis and/or demethylation. Indeed, marine bacteria are thought to favour DMSP cleavage over DMSP demethylation, upon exposure to high DMSP concentrations [21, 73]. However, note that a recent study showed that DMSP demethylation required μM DMSP levels, far higher than those for DMSP lysis (>35 nM), to induce transcription of these competing *dmd* and *ddd* catabolic genes [74].

This study also provides a new strategy to identify key microorganisms degrading DMS as well as DMSP for carbon assimilation in environmental samples. The use of ^13^C-DMSP where only the propionate was ^13^C-labelled and not the DMS moiety efficiently identified members of the *Piscirickettsiaceae* family, especially *Methylophaga* (well known for their ability to grow on DMS [70]), as important bacteria likely degrading the DMS generated from DMSP for carbon assimilation in these coastal seawater samples. Thus, it will be interesting to use the SIP strategy applied in this work on more varied marine environments to elucidate the variability in those distinct microorganisms using DMSP and/or DMS primarily for carbon requirements.

Although wide-ranging conclusions have been drawn from the experiments conducted here on one coastal site, it should be noted that there (i) may be considerable variation in the types of microorganisms using DMSP and/or DMS in distinct environments and with different levels of available DMSP and (ii) was no gene/protein expression work complementing the DNA-SIP experiments, and thus however plausible the conclusions made here are, further work is required to establish that, e.g. the *Amphritea dddD* genes are expressed in the presence of DMSP. Nevertheless, this study has deepened our understanding of microbial DMSP degradation, and it provides a note of caution that analysis of environmental ‘omics data for taxonomy and gene abundance alone can lead to significant misrepresentation of the importance and/or role of microbial groups in this process.

## Methods

### Sampling and quantification of DMSP and DMS in natural coastal seawater

North Sea surface seawater was collected from Great Yarmouth coast, UK, (52° 35′ 27.5928″ N; 1°44′ 17.9268″ E) on 27^th^ January 2018. To study the composition of the natural (T0) microbial community, 3 L of coastal seawater was filtered through 0.22-μm polycarbonate membrane filters (Millipore Corporation) using a vacuum pump. Filters were then stored at −80 °C for DNA extraction.

DMS concentrations in natural (T0) coastal seawater samples were quantified using a purge-and-trap gas chromatography (GC) system [75] using a flame photometric detector (Agilent 7890A GC fitted with a 7693 autosampler) and a HP-INNOWAX 30 m × 0.320 mm capillary column (Agilent Technologies J&W Scientific). DMSP content was quantified indirectly via alkaline lysis as previously described [76]. An eight-point calibration curve with DMS standards was made as in [76]. The detection limit for DMS in the headspace was 0.8 pmol.

### DNA-stable isotope probing experiments

#### Synthesis of ^13^C-DMSP

^13^C-DMSP was synthesised in house from acrylic acid-^13^C_3_ and DMS (Sigma-Aldrich) as described in [31].

#### DNA-SIP experiments with ^13^C-labelled DMSP

750 mL of coastal seawater were incubated in 2 L air-tight bottles containing 100 μM of either ^12^C- (control) or ^13^C-labelled DMSP (Fig. 2a). To ensure the recovery of enough ^13^C-labelled DNA for 16S and MG sequencing, six incubations with ^12^C- and six with ^13^C-DMSP were set up. All samples were incubated at 22 °C for 96 h (Fig. 2b) on a 12-h light (4000 lx) and 12-h dark cycle. After 96 h, when 77 μmol DMSP L^−1^ (231 μmol C L^−1^) were assimilated, the six biological replicates from ^12^C- and ^13^C-DMSP incubations were combined in pairs, respectively, and filtered through 0.22-μm polycarbonate membrane filters, resulting in triplicate samples used for subsequent DNA extraction.

#### Quantification of DMSP catabolism

DMSP, DMS and MeSH concentrations during SIP incubations with ^12^C- and ^13^C-DMSP were monitored by GC using the instrument and column cited above. To measure DMS and MeSH concentrations, 50 μl of headspace from ^12^C- and ^13^C-DMSP incubations were injected in the GC. To measure DMSP content, 1 mL seawater aliquots were taken from each replicate at selected timepoints and subsequently sparged with nitrogen to remove gaseous compounds. DMSP concentration was then measured via the addition of 10 M NaOH to 200 μL of the seawater samples in 2 mL gas-tight vials as described in [52]. Subsequent liberation of DMS was quantified by GC as above. An eight-point calibration curve of DMS and MeSH standards was used as in [52]. The detection limits for DMS and MeSH were 0.15 and 4 nmol, respectively.

#### Separation of labelled and unlabelled DNA

DNA from filters of natural (T0) seawater samples and incubations with ^12^C- and ^13^C-DMSP was extracted as in [77]. Four micrograms of DNA from ^12^C- and ^13^C-DMSP incubations was separated into heavy (^13^C-labelled) and light (^12^C-unlabelled) DNA by isopycnic ultracentrifugation as previously described [78]. DNA in each fraction was quantified using a Qubit dsDNA HS Assay kit (ThermoFisher Scientific) following the manufacturer’s instructions. The density of each fraction was determined by refractometry using a Reichert AR200 refractometer (Reichert Analytical Instruments). Heavy and light DNA fractions from each sample were identified by plotting DNA abundance vs refractive index (as a proxy for density; Fig. 2c) and used for subsequent downstream analysis.

### 16S rRNA gene amplicon sequencing

To investigate the microbial diversity in samples from DNA-SIP experiments, we used DNA from three biological replicates from unenriched (T0) samples and labelled (heavy; H) and unlabelled (light; L) fractions from ^12^C- and ^13^C-DMSP incubations. 16S rRNA genes were amplified with primers 515F and 806R [79, 80]. Triplicate PCR reactions for each sample were pooled before purification of PCR amplicons. Pyrosequencing was performed on Illumina MiSeq PE300 platform at Majorbio Bio-Pharm Technology Co. Ltd., (Shanghai, China), obtaining an average of 54936 quality-filtered reads per sample with an average length of 273 bp (Table S7). Sequences were analysed using Qiime [81], chimeras were excluded and OTUs assigned based on 97% similarity level. Taxonomic assignment was made using the SILVA database (Release 123 [82];) with 80% similarity threshold. Statistically significant differences in the RA of microbial groups from different samples were analysed using Student’s *t*-test. The total microbial community from samples incubated with ^12^C- (12C_T) and ^13^C-DMSP (13C_T) prior to fractionation was reconstructed in silico, by adding the contributions from both light and heavy DNA, in proportion to the relative amount of DNA in the fractions.

### Denaturing gel gradient electrophoresis (DGGE)

Bacterial 16S rRNA genes from DNA fractions from SIP incubations with ^12^C- and ^13^C-DMSP were amplified using primers 314F-GC and 518R [83]. Denaturing gel gradient electrophoresis (DGGE) was performed to visualise 16S rRNA gene profiles of the bacterial communities from ^12^C- and ^13^C-DMSP incubations following the protocol described by Green *et al*. [84].

### Metagenomic analysis of DNA-SIP samples

Three biological replicates from natural (T0) seawater samples were subjected to metagenomic sequencing. As biological replicates from ^13^C-heavy (13C_H) and ^13^C-light (13C_L) fractions from incubations with ^13^C-labelled DMSP showed highly similar 16S rRNA gene profiles in the DGGE analysis (Fig. S7), they were combined in equal proportions prior to metagenomic sequencing. Libraries from all samples were prepared by BGI (Shenzhen, China) for metagenomic sequencing without any amplification step. Shotgun sequencing was performed on an Illumina HiSeq X-Ten platform, with 2 × 150 bp paired-end reads. Metagenomic reads were quality-filtered and trimmed using SOAPnuke [85], obtaining a range of 13.2 to 14.1 Gb of high quality data per sample, with Q30 of each sample >90% (Table S8). Filtered reads were initially assembled by IDBA_UD [86] with different k-mer values for each sample. SOAP*denovo*2 [87] was used to map reads from each sample to the assembled data, then combined with the N50 and mapping rates to choose the optimum k-mer and corresponding assembly results. Contigs shorter than 300 bp were excluded. Gene prediction was performed with MetaGeneMark [88]. Redundant sequences were removed using CD-Hit [89] at 95% identity and 90% coverage. Gene taxonomic annotation was determined by BLASTp (E ≤ 1e-5) against NCBI-nr databases using MEGAN software [90]. The RA of each gene was calculated as the percentage of its sequence coverage to the total sequence coverage (see Eq. 1). The sequence coverage was determined by the length of mapped reads relative to the reference sequence length.

1 $${a}_i=\frac{b_i}{\sum_j{b}_j}=\frac{\frac{X_i}{L_i}}{\sum_j\frac{X_j}{L_j}}$$

where *a*_*i*_ is the relative abundance of gene “*i*”; *b*_*i*_, the copy number of gene “*i*”; *j*, the total number of genes; *X*_*i*_, the total reads number aligned to gene “*i*”; and *L*_*i*_, the length of gene “*i*”.

The sum of the RA of genes taxonomically assigned to a particular taxon was used to report the RA of that microbial group in metagenomic samples.

Profile Hidden Markov Model (HMM)-based searches for proteins of interest in metagenome datasets were performed using HMMER tools (v.3.1, http://hmmer.janelia.org/). Sequences of interest included DMSP synthesising enzymes (DSYB, TpMMT, DsyB and MmtN), DMSP lyases (DddD, DddL, DddP, DddQ, DddW, DddY, DddK, DddX and Alma1) and ancillary enzymes (DddA, DddB, DddC, DddT, AcuN, AcuK, PrpE and AcuI), DMSP-demethylase (DmdA, DmdB, DmdC and DmdD), DMS monooxgenase (DmoA), DMS dehydrogenase (DdhA), Trimethylamine monooxygenase (Tmm), Dimethyl sulfoxide reductase (DMSOR), MeSH S-methyltransferase (MddA) and methanethiol oxidase (MTO). Protein sequences ratified as functional in previous studies (Table S9) were used as training sequences to create the HMM profiles. HMM searches were performed against unique hits from seawater metagenomes using a cut-off value of *E* ≤ 1e−30 for DMSP cycling proteins and *E* ≤ 1e−5 for most DMS cycling proteins [3, 91] (Table S1). Each potential sequence of interest retrieved from the analysis of seawater metagenomes was manually curated by BLASTp against the database of reference sequences (Table S9) and discarded if they had <40% amino acid identity to the corresponding ratified proteins. Given the large size of known DddD polypeptides [22, 31], retrieved sequences <800 amino acids were excluded. Resultant sequences were used to construct a phylogenetic tree using MEGA v5.0 [92] with the Poisson model substitution model. Tree topology was checked using 100 bootstrap replicates. Only sequences whose top hits belonged to the same protein family and clustered with ratified sequences detailed in Table S9 were counted.

Finally, to determine the RA of bacteria and eukaryotes containing genes of interest, the number of unique hits of bacterial genes in seawater metagenomes was normalised to *recA* (a single copy marker gene possessed by the vast majority of bacteria), whereas unique hits of algal genes were normalised to ACTB (a phylogenetic marker gene for eukaryotes, encoding β-Actin protein) [3]. Hits of *recA* and ACTB were determined by HMM searches (E ≤ 1e−5) of sequences from a database obtained from RDP’s FunGene [93] for *recA* and from Uniprotkb/swiss-prot for ATCB [94]. The RA of bacteria containing genes of interest in the total microbial community from incubations with ^13^C-labelled DMSP prior fractionation (13C_T) was calculated as above.

Co-assembly of metagenomes from T0 samples and ^13^C-heavy and ^13^C-light fractions from incubations with ^13^C-DMSP was performed to reconstruct metagenome-assembled genomes (MAGs) using MetaBAT2 [95]. Completeness and contamination of MAGs was assessed using CheckM [96], and only MAGs with <5% contamination were considered for further analysis. The phylogeny of MAGs was determined using PhyloPhlAn [97], followed by average nucleotide identity (ANI) calculations to further confirm taxonomy. The RA of each MAG in seawater metagenomes was calculated by the formula “MAG coverage / genome equivalent”. The MAG coverage (copy number) of MAGs in each metagenome was evaluated using Anvi’o [98], by adding the coverage of each nucleotide in a MAG and dividing it by the MAG length. The genome equivalent of metagenomic samples (the total number of genomes per metagenome sample) was estimated by MicrobeCensus [99].

### Isolation and characterisation of DMSP-degrading bacteria

Seawater from SIP incubations with ^12^C- and ^13^C-DMSP was serially diluted and plated onto marine broth agar, marine basal medium (MBM) supplemented with 10 mM mixed carbon source (glucose, succinate, sucrose, pyruvate and glycerol at 2 mM each) and MBM supplemented with 2 mM DMSP as sole carbon source [46]. After 48 h of incubation at 30 °C, sixty-six colonies with distinct morphologies were isolated and purified. To test the ability of the isolates to degrade DMSP, 300 μl of cultures grown on MBM with 10 mM mixed carbon source was transferred to 2-mL serum vials and supplemented with 0.5 mM DMSP. After 2-h incubation at 30 °C, DMS and MeSH liberated from DMSP was measured by GC. Media-only vials were set up as abiotic controls. Cellular protein content was estimated by Bradford assays (BioRad). Rates of DMS production are expressed as nmol·mg protein^−1^·h^−1^.

To determine if isolates could use DMSP as sole carbon source for growth, they were grown in MBM with mixed carbon source, pelleted and washed twice with MBM medium without any carbon source. Cultures were then inoculated 1% (v/v) in triplicate into fresh MBM medium containing no carbon source, 10 mM mixed carbon source or 2 mM DMSP. Cultures were incubated at 30 °C for 10 days and growth was estimated by measuring cell density at OD_600_ with a spectrophotometer. The statistical differences between the negative control (no carbon source) and cultures supplemented with DMSP or mixed carbon addition were determined by Student’s *t*-test (P < 0.05 signifying the ability to grow on DMSP).

For identification, genomic DNA from DMSP-degrading strains was extracted using a Wizard genomic DNA purification kit (Promega) and 16S rRNA genes were amplified using primers 27F/1492R [100]. Purified PCR products were sequenced by Eurofins Genomics (Munich, Germany) and isolates were taxonomically identified using the Ezbiocloud website (http://www.ezbiocloud.net/identify). Representative strains from each genera/species were selected for further bioinformatics analysis to predict their molecular mechanisms to cleave DMSP. Publicly available genomes of their most closely related reference strains were screened for the presence of *dmd/ddd* homologous genes. Homologue sequences to ratified proteins involved in DMSP cycling (Table S9) were identified using local BLASTp, with thresholds set as E ≤ 1e−30, ≥50% amino acid identity and ≥70% coverage.

### Genomic sequencing of potential DddD-containing strains

The genomes of *Pseudomonas* sp. D13-4, *Marinobacter* sp. GY8 and four representative *Oceanospirillales* bacteria, *Cobetia* sp. MC13-5, *Amphritea* sp. GY6, *Marinobacterium* sp. D13-1 and *Marinomonas* sp. MB12-11, were sequenced using the PacBio RS II and Illumina HiSeq 4000 platforms at the Beijing Genomics Institute (GHI, Shenzhen, China). SMRT cells Zero-Mode Waveguide arrays of sequencing were used by the PacBio platform to generate the subreads set. Subreads <1 kb were removed. The Pbdagcon programme (https://github.com/PacificBiosciences/pbdagcon) was used for self-correction. Assembly of the combined short and long reads from Illumina and PacBio platform was done using Unicycler [101] to obtain full-length genomes.

Quality of assembled genomes was analysed with CheckM [96], resulting in ≥99.1% completeness and <1.3% contamination. Genes were predicted using Glimmer 3.02 [102]. Identification of genes encoding homologous proteins to ratified DMSP cycling enzymes was performed using BLASTp, as described above.

## Supplementary Information

**Additional file 1: Figure S1**. Microbial taxonomic profiles of seawater samples at the domain, phylum and class levels. **Figure S2**. Potential eukaryotic sources of DMSP in the natural (T0) coastal seawater. **Figure S3**. Potential prokaryotic sources of DMSP in the natural (T0) coastal seawater. **Figure S4**. Maximum likelihood phylogenetic tree of DmdA proteins. **Figure S5**. Maximum likelihood phylogenetic tree of DddP proteins. **Figure S6**. Maximum likelihood phylogenetic trees of DddQ, DddL, DddK, DddY, DddW and DddX proteins. **Figure S7**. 16S rRNA gene profiles of seawater samples enriched with DMSP analysed by DGGE. **Figure S8**. Bacterial community profiles of coastal seawater samples analysed by 16S rRNA gene sequencing. **Figure S9**. Relative abundance and taxonomy of ancillary genes from the DMSP demethylation pathway in coastal seawater samples. **Figure S10**. Relative abundance and taxonomic affiliation of ancillary genes from the DMSP cleavage pathway in coastal seawater metagenomes. **Figure S11**. Relative abundance and taxonomic affiliation of DMS cycling genes in coastal seawater samples. **Table S1**. Relative abundance of genes encoding proteins involved in the cycling of DMSP, DMS and related compounds in metagenomes from seawater samples. **Table S2**. Relative abundance (RA) of main bacterial genera from seawater samples analysed by 16S rRNA gene amplicon (16S) and metagenomics (MG) sequencing. **Table S3**. Dominant bacterial genera in T0 seawater samples analysed by 16S rRNA gene amplicon sequencing. **Table S4**. Relative abundance (RA) of main bacterial orders from seawater samples analysed by 16S rRNA gene amplicon (16S) and metagenomics (MG) sequencing. **Table S5**. Metagenome-assembled genomes (MAGs) with homologous sequences to genes involved in DMSP cycling reconstructed from metagenomes from seawater samples. **Table S6**. Characteristics of bacterial strains with DMSP-degrading activity isolated from seawater incubations with DMSP. **Table S7**. 16S rRNA gene amplicon sequencing results for coastal seawater samples. **Table S8**. Statistics for metagenomic sequencing and assemblies. **Table S9**. Accession numbers of previously ratified enzymes involved in the cycling of DMSP and related compounds.

## Data Availability

16S rRNA gene amplicon sequencing and metagenomic data generated in this study were deposited to the sequence read archives (SRA) under Bioproject PRJNA685000. The accession numbers for genomes from bacterial strains isolated in this work and MAGs reconstructed from seawater metagenomes are listed in Table 1 and Table S5, respectively.
