# Supplementary material for: Oceanospirillales containing the DMSP lyase DddD are key utilisers of carbon from DMSP in coastal seawater
Source: Microbiome. 2022 Jul 27;10:110. doi: 10.1186/s40168-022-01304-0 (PMC9327192; doi:10.1186/s40168-022-01304-0)
Supplement: Supplementary file 2 — Additional file 1: Figure S1. Microbial taxonomic profiles of seawater samples at the domain, phylum and class levels. Figure S2. Potential eukaryotic sources of DMSP in the natural (T0) coastal seawater. Figure S3. Potential prokaryotic sources of DMSP in the natural (T0) coastal seawater. Figure S4. Maximum likelihood phylogenetic tree of DmdA proteins. Figure S5. Maximum likelihood phylogenetic tree of DddP proteins. Figure S6. Maximum likelihood phylogenetic trees of DddQ, DddL, DddK, DddY, DddW and DddX proteins. Figure S7. 16S rRNA gene profiles of seawater samples enriched with DMSP analysed by DGGE. Figure S8. Bacterial community profiles of coastal seawater samples analysed by 16S rRNA gene sequencing. Figure S9. Relative abundance and taxonomy of ancillary genes from the DMSP demethylation pathway in coastal seawater samples. Figure S10. Relative abundance and taxonomic affiliation of ancillary genes from the DMSP cleavage pathway in coastal seawater metagenomes. Figure S11. Relative abundance and taxonomic affiliation of DMS cycling genes in coastal seawater samples. Table S1. Relative abundance of genes encoding proteins involved in the cycling of DMSP, DMS and related compounds in metagenomes from seawater samples. Table S2. Relative abundance (RA) of main bacterial genera from seawater samples analysed by 16S rRNA gene amplicon (16S) and metagenomics (MG) sequencing. Table S3. Dominant bacterial genera in T0 seawater samples analysed by 16S rRNA gene amplicon sequencing. Table S4. Relative abundance (RA) of main bacterial orders from seawater samples analysed by 16S rRNA gene amplicon (16S) and metagenomics (MG) sequencing. Table S5. Metagenome-assembled genomes (MAGs) with homologous sequences to genes involved in DMSP cycling reconstructed from metagenomes from seawater samples. Table S6. Characteristics of bacterial strains with DMSP-degrading activity isolated from seawater incubations with DMSP. Table S7. 16S rRNA gene amplicon sequencing results [file 40168_2022_1304_MOESM1_ESM.docx]

**Additional File 1**

***Oceanospirillales* containing the DMSP lyase DddD are key utilisers of carbon from DMSP in coastal seawater**

Jingli Liu^1,2^, Chun-xu Xue^1^, Jinyan Wang^1,2^, Andrew T. Crombie^3^, Ornella Carrión^1^, Andrew W. B. Johnston^2^, J. Colin Murrell^3^, Ji Liu^1,2^, Yanfen Zheng^1,2^, Xiao-Hua Zhang^1,4*^, Jonathan D. Todd^2*^

^1^ College of Marine Life Sciences, and Frontiers Science Center for Deep Ocean Multispheres and Earth System, Ocean University of China, Qingdao, China.

^2^ School of Biological Sciences, University of East Anglia, Norwich Research Park, Norwich, UK.

^3^ School of Environmental Sciences, University of East Anglia, Norwich Research Park, Norwich, UK.

^4^ Laboratory for Marine Ecology and Environmental Science, Qingdao National Laboratory for Marine Science and Technology, Qingdao, China.

***Correspondence:**

Dr. Jonathan D. Todd, jonathan.todd@uea.ac.uk.

Dr. Xiao-Hua Zhang, [xhzhang@ouc.edu.cn](mailto:xhzhang@ouc.edu.cn).

**
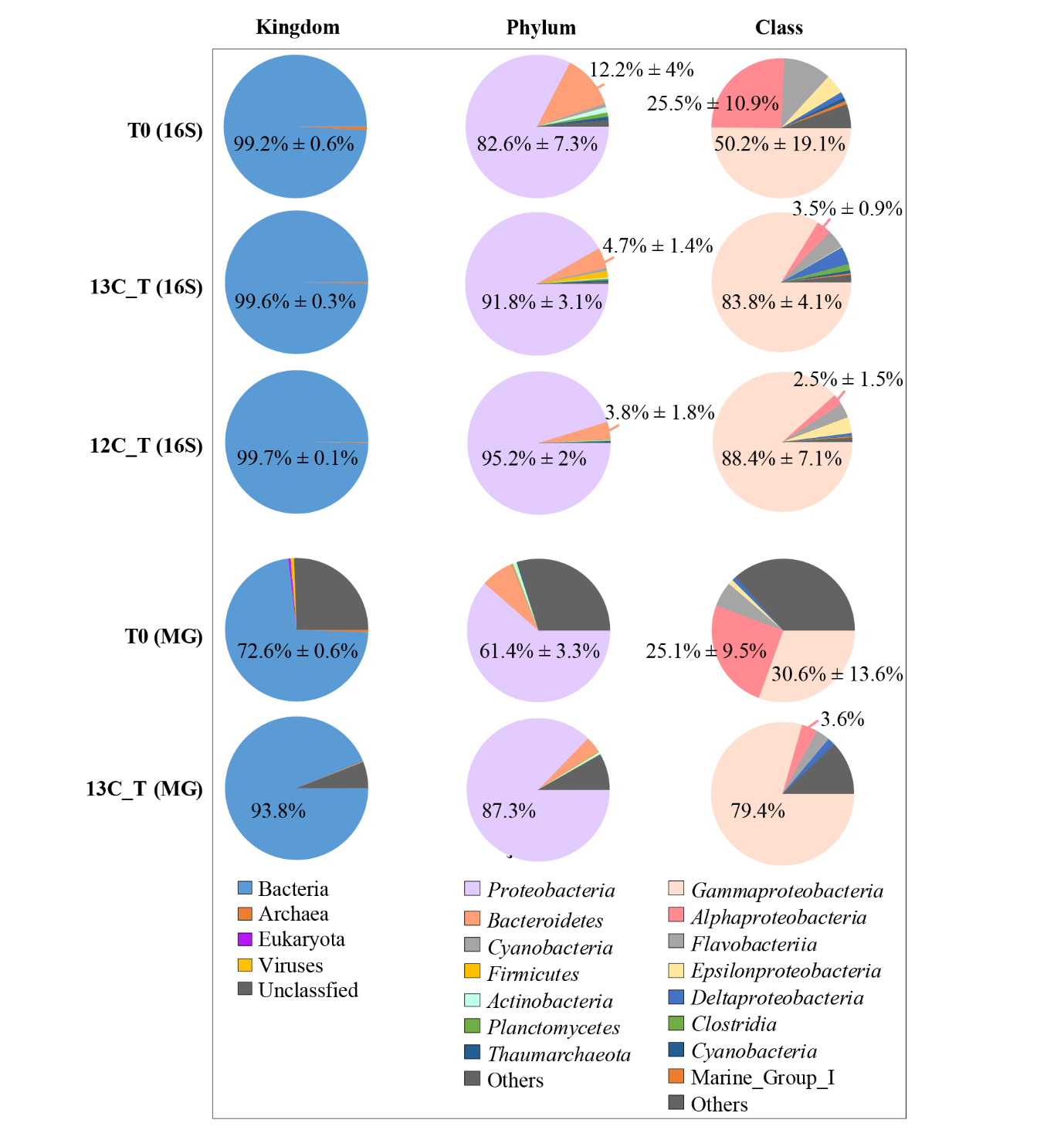
**

**Fig. S1 Microbial taxonomic profiles of seawater samples at the domain, phylum and class levels.** Microbial diversity of natural (T0) coastal seawater and from samples incubated with ^13^C-DMSP or ^12^C-DMSP (control) analysed by 16S rRNA gene amplicon (16S) and metagenomic (MG) sequencing. Pie charts represent the average of three biological replicates, except for 13C_T samples, for which biological replicates were combined before MG sequencing (see Methods).


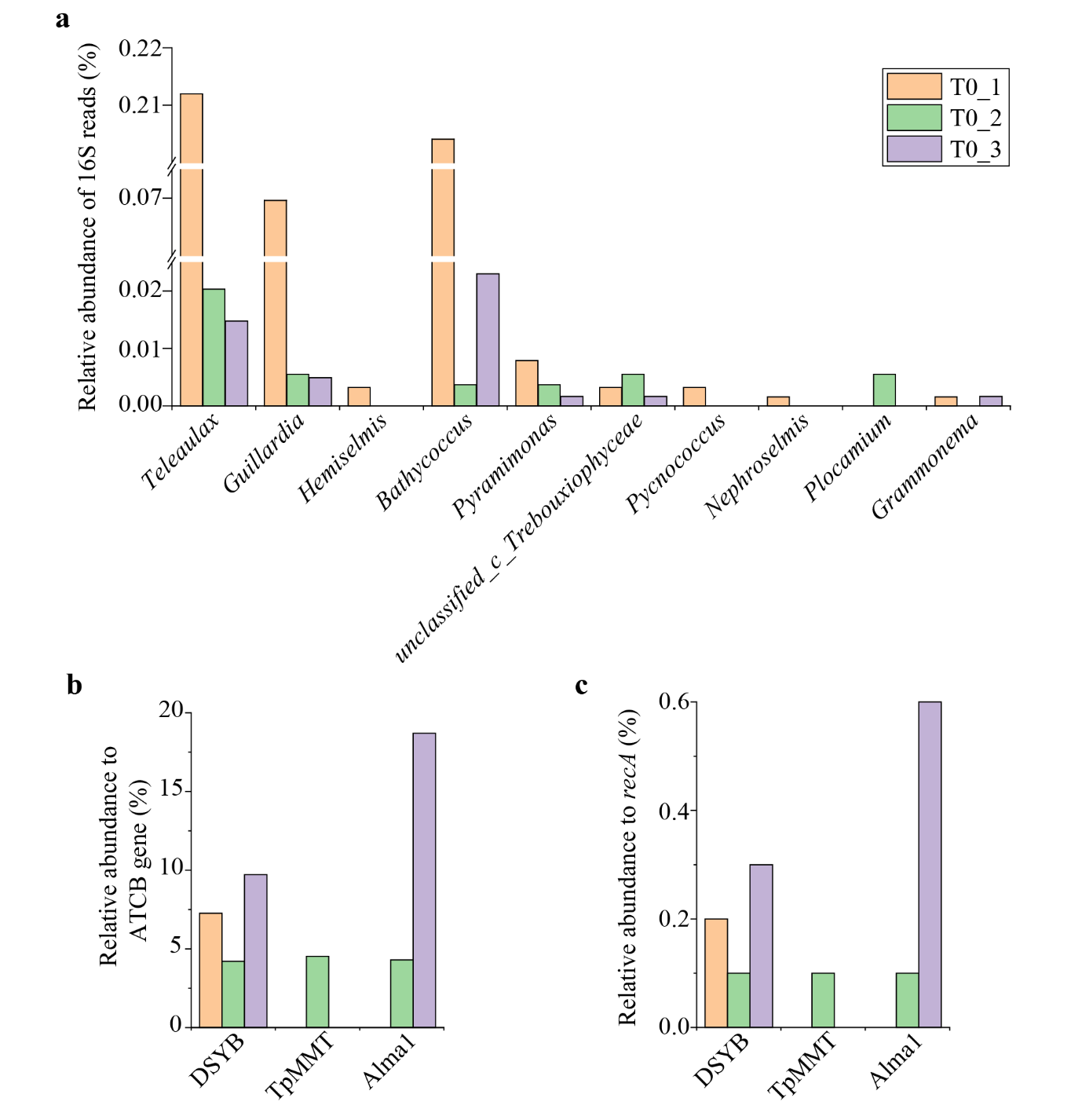


**Fig. S2 Potential eukaryotic sources of DMSP in the natural (T0) coastal seawater.** **(a)** The relative abundance (RA) of potential eukaryotic DMSP producers in natural (T0) seawater samples was calculated as percentage of their corresponding plastid sequences in the total microbial community analyzed by 16S rRNA amplicon sequencing. **(b)** RA of eukaryotic DMSP synthesis (*DSYB* and *TpMMT*) and degradation (*Alma1*) genes in T0 metagenomes. The number of unique hits of each gene was normalized to the number of eukaryotic ATCB sequences present in each T0 metagenome. **(c)** RA of eukaryotic DMSP synthesis genes normalized to bacterial *recA* sequences.


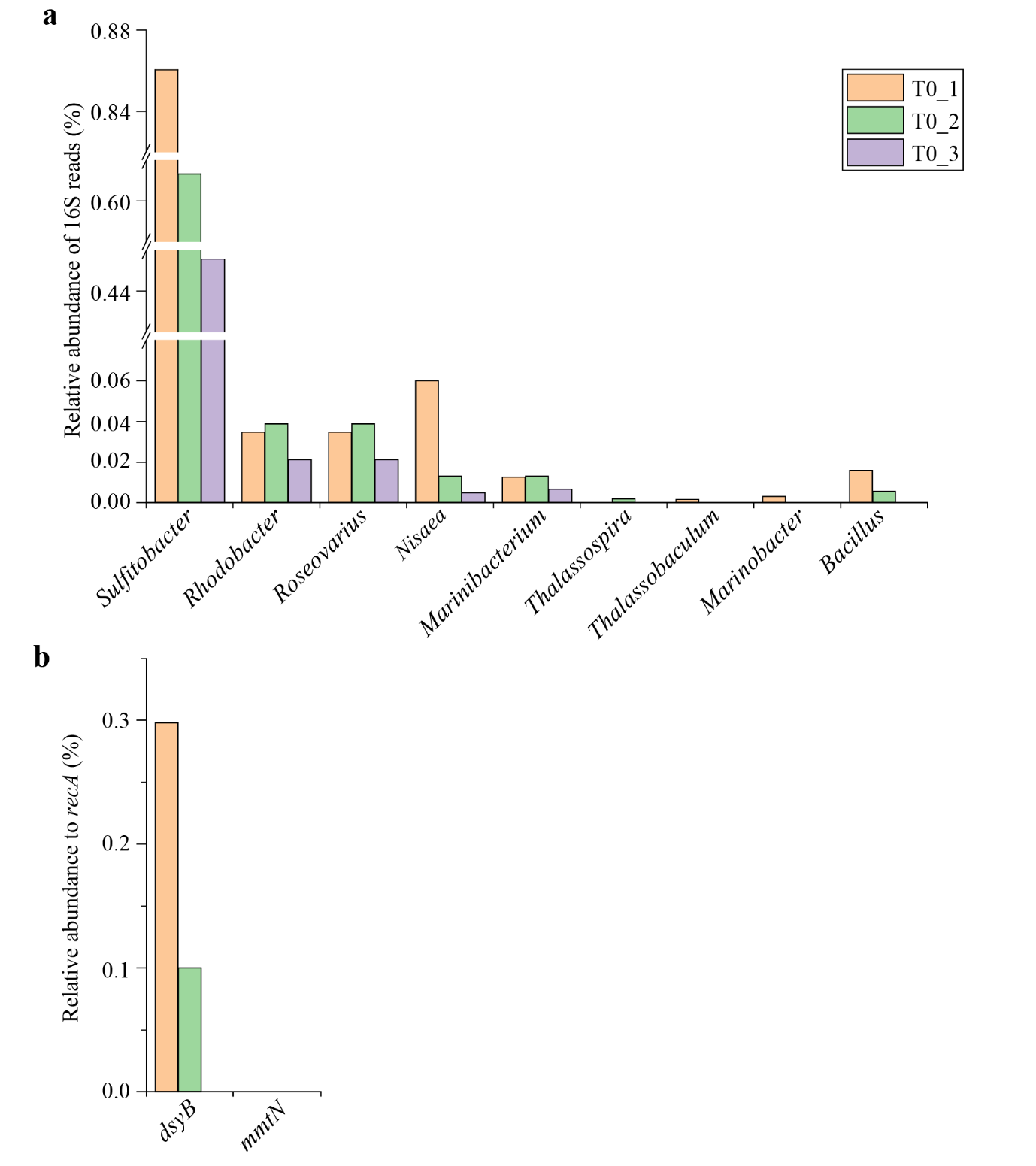


**Fig. S3 Potential prokaryotic sources of DMSP in the natural (T0) coastal seawater.** **(a)** Relative abundance (RA) of bacterial genera known to contain DMSP-producing strains and/or DMSP synthesis genes (*dsyB* and *mmtN*) in T0 seawater samples analysed by 16S rRNA gene amplicon sequencing. **(b)** RA of bacterial DMSP synthesis genes in T0 metagenomes. The number of unique hits of *dsyB* and *mmtN* genes was normalized to the number of *recA* sequences present in each T0 metagenome.


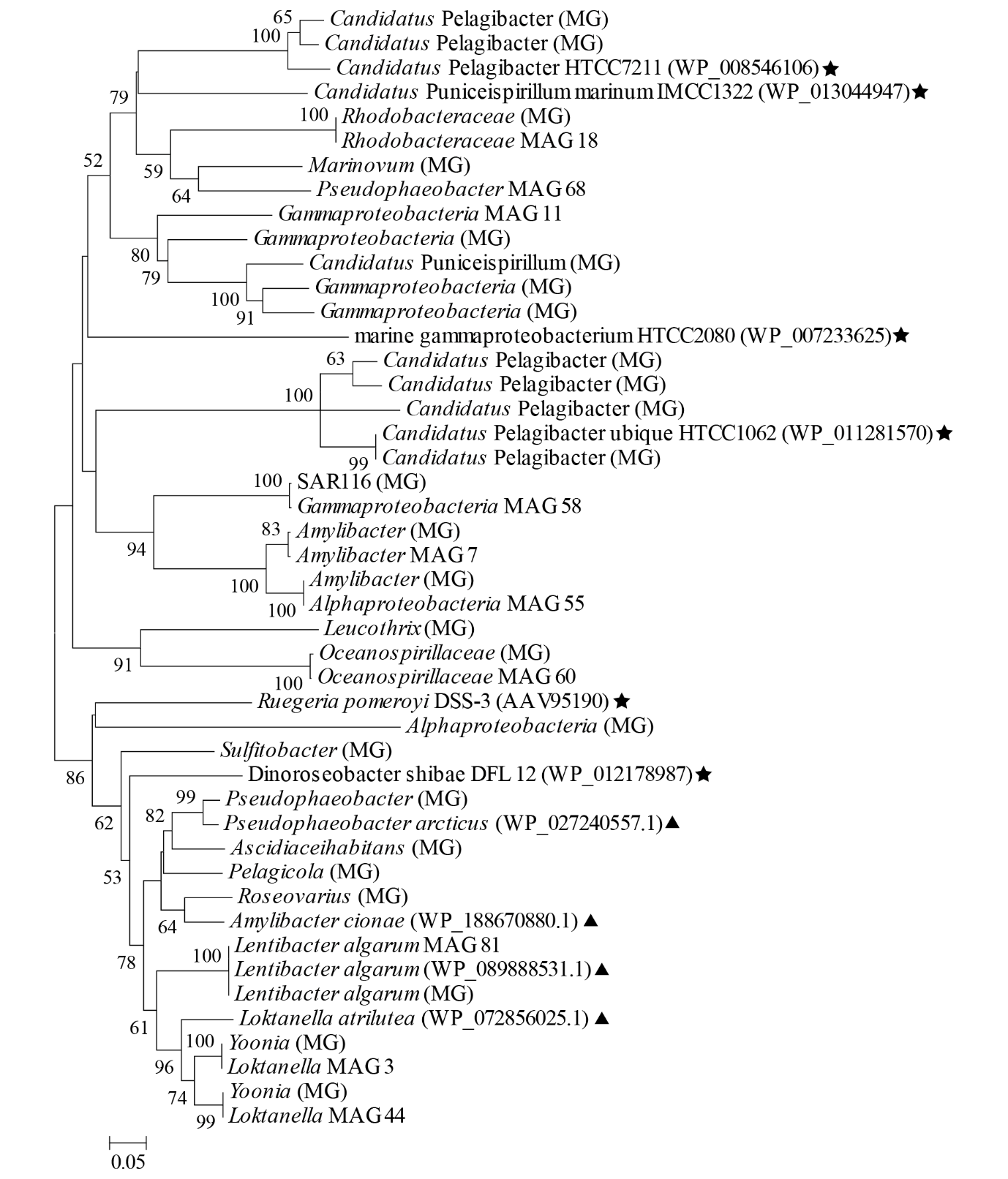


**Fig. S4** **Maximum likelihood phylogenetic tree of DmdA proteins.** A phylogenetic tree of DmdA proteins was constructed with sequences from previously ratified enzymes (★) together with those retrieved from seawater metagenomes (MG), metagenome-assembled genomes (MAG) and reference strains (▲) using MEGA v5.0 [1]. Bootstrap values ≥ 50% (based on 100 replicates) are shown. Scale bar indicates 5% estimated phylogenetic divergence.


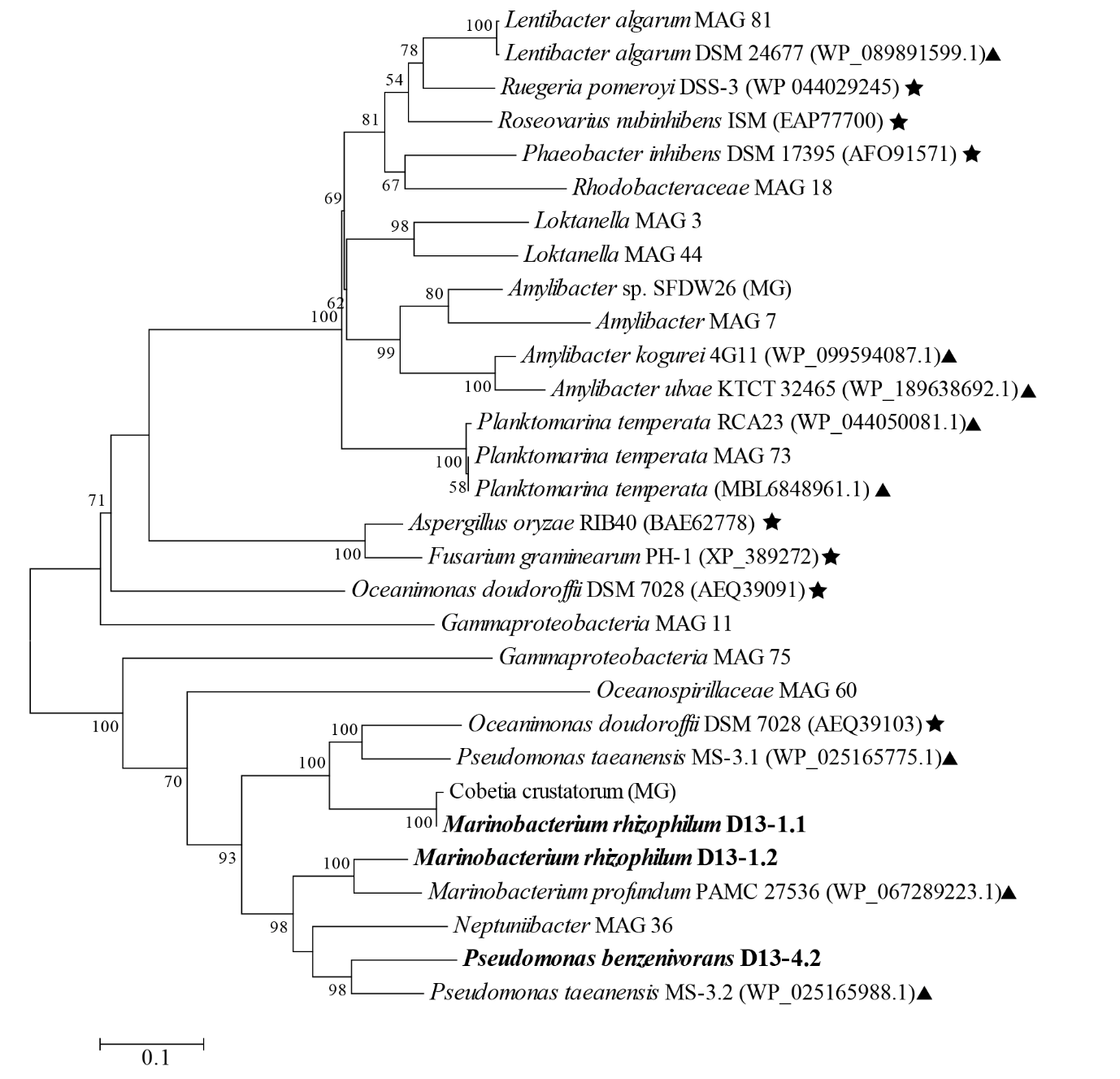


**Fig. S5** **Maximum likelihood phylogenetic tree of DddP proteins.** The tree shows proteins sequences from strains isolated in this study (in bold), and previously ratified proteins (★) together with those retrieved from metagenomes (MG), metagenome-assembled genomes (MAG) and reference strains (▲). Bootstrap values ≥ 50% (based on 100 replicates) are shown. Scale bar indicates 10% estimated phylogenetic divergence.


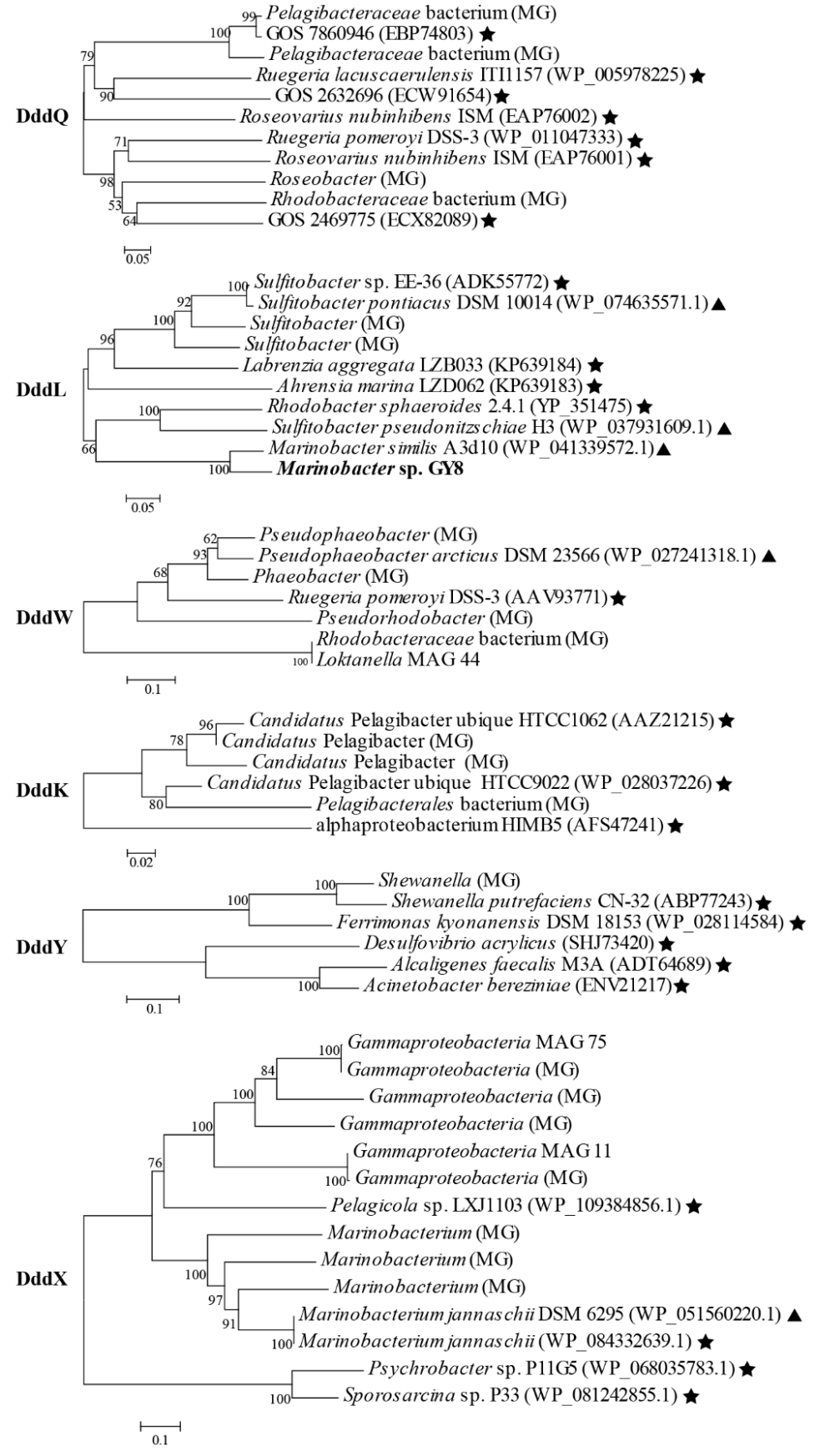


**Fig. S6 Maximum likelihood phylogenetic trees of DddQ, DddL, DddK, DddY, DddW and DddX proteins.** Phylogenetic trees were constructed using sequences from a strain isolated in this study (in bold), previously ratified enzymes (★) and proteins retrieved from metagenomes (MG), metagenome-assembled genomes (MAG), and reference strains (▲). Bootstrap values ≥ 50% (based on 100 replicates) are shown. Scale bars indicate estimated phylogenetic divergence.


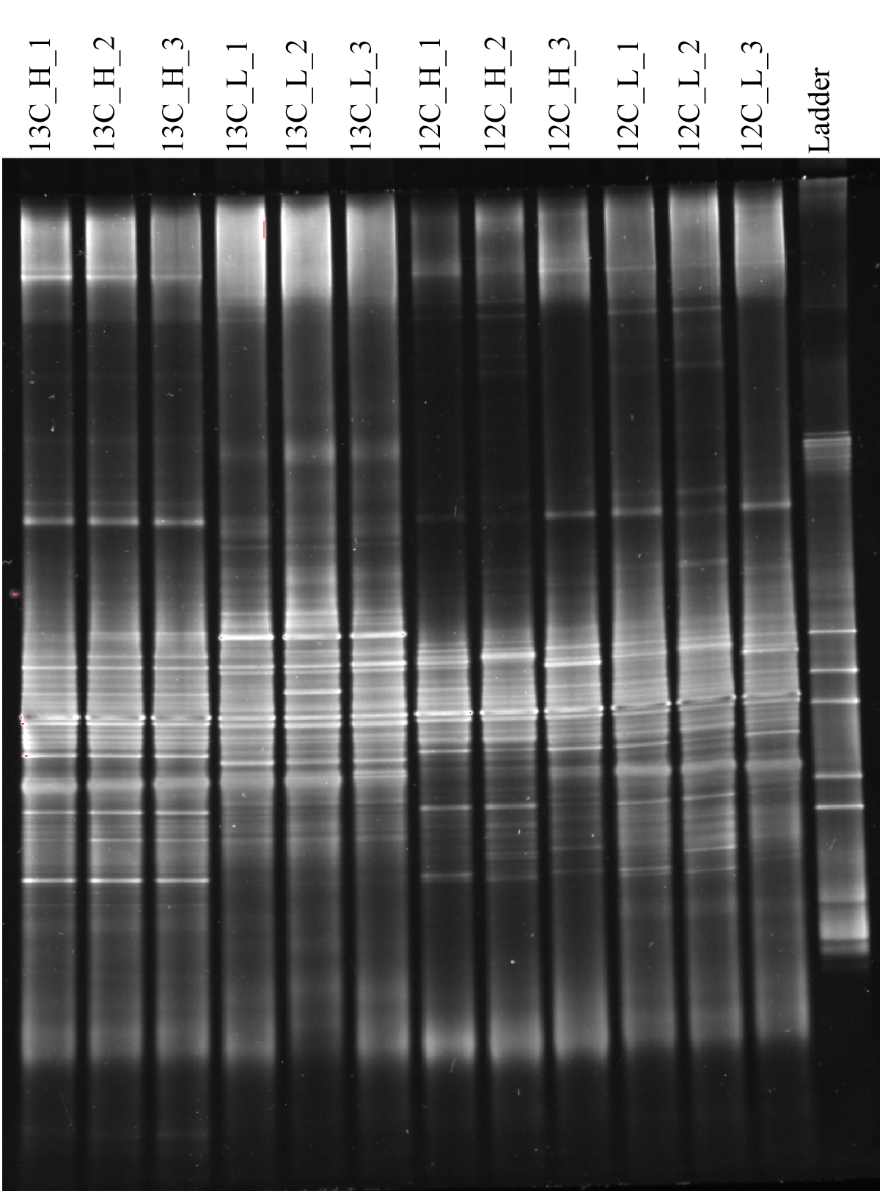


**Fig. S7** **16S rRNA gene profiles of seawater samples enriched with DMSP analysed by DGGE.** 13C_H: heavy fractions from samples incubated with ^13^C-DMSP; 13C_L: light fractions from ^13^C-DMSP incubations; 12C_H: heavy fractions from samples incubated with ^12^C-DMSP; 12C_L: light fractions from ^12^C-DMSP incubations.

**
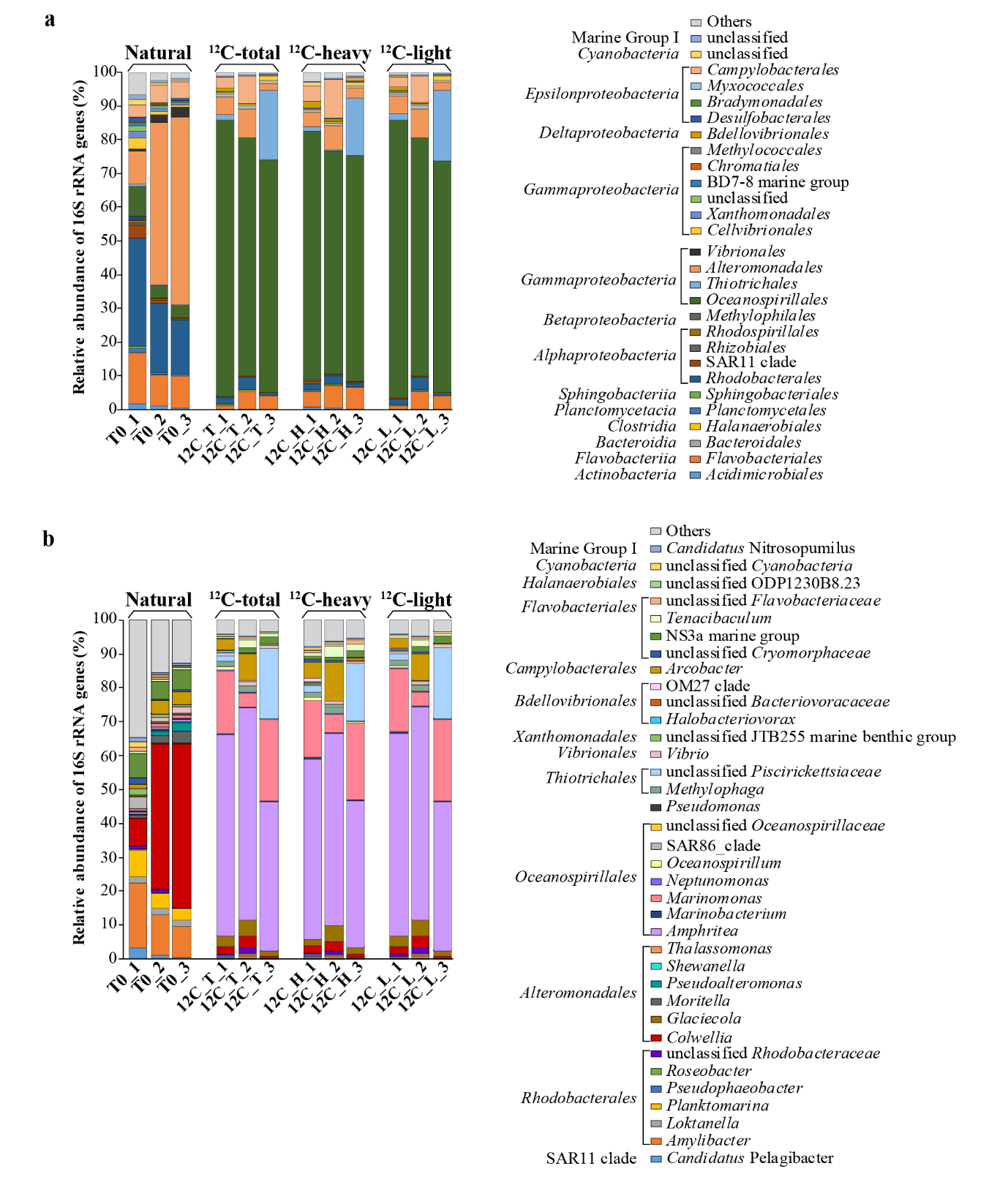
**

**Fig. S8 Bacterial community profiles of coastal seawater samples analysed by 16S rRNA gene sequencing.** T0: natural seawater samples; 12C_T: total microbial community from samples incubated with ^12^C-DMSP; 12C_H: heavy fractions from ^12^C-DMSP incubations; 12C_L: light fractions from ^12^C-DMSP incubations. Genera with <0.5% relative abundance in one of the conditions are grouped in “Others”.

**
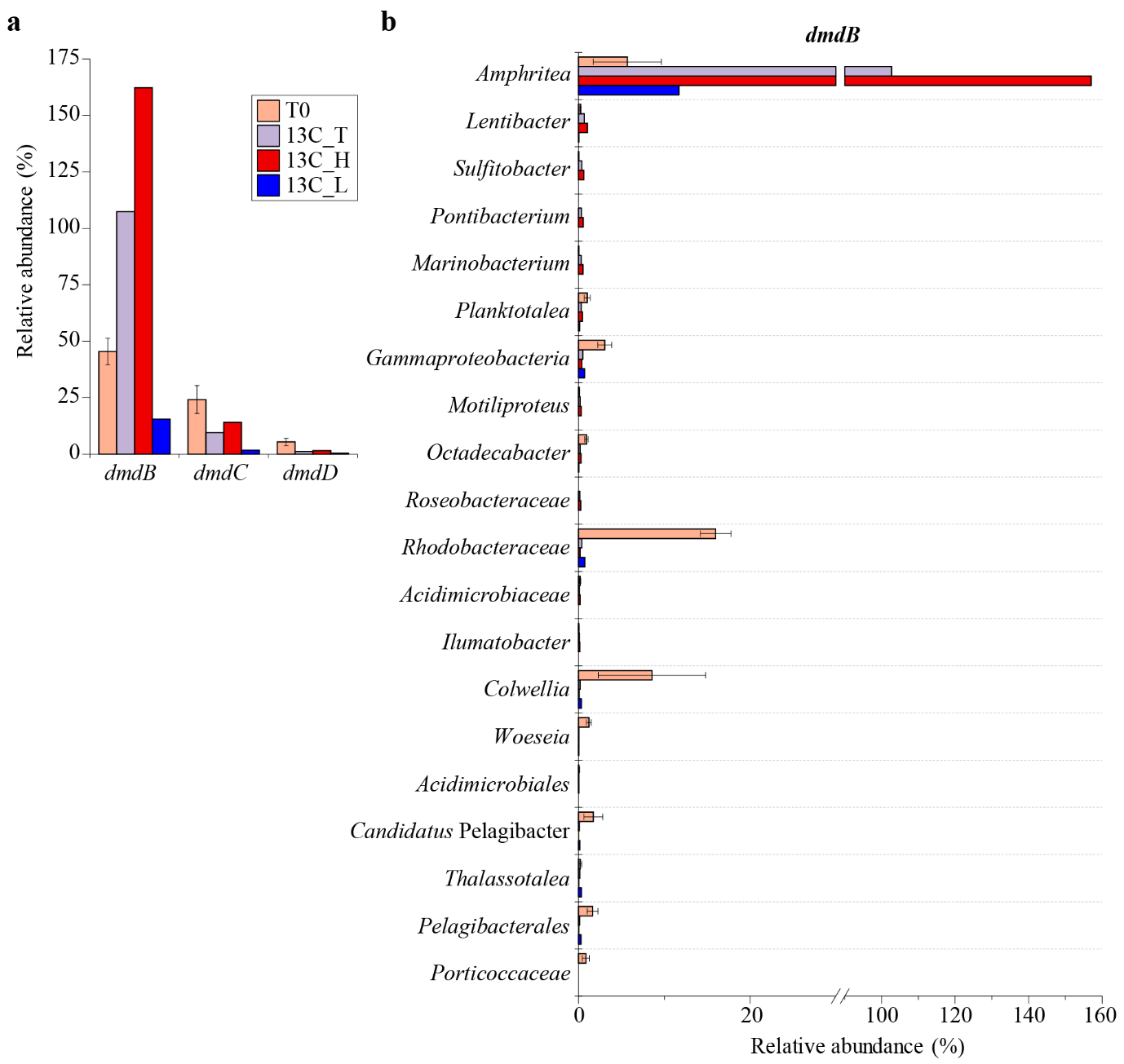
**

**Fig. S9 Relative abundance and taxonomy of ancillary genes from the DMSP demethylation pathway in coastal seawater samples.** **(a)** Relative abundance (RA) of ancillary genes involved in DMSP demethylation pathway in seawater metagenomes **(b)** Taxonomic affiliation of *dmdB* sequences retrieved from seawater metagenomes. T0: metagenomes from natural samples; 13C_T: total microbial community from samples incubated with ^13^C-DMSP. 13C_H: metagenomes from ^13^C-heavy fractions; 13C_L: metagenomes from ^13^C-light fractions. T0 values represent the average of three biological replicates. Biological replicates from ^13^C-heavy and ^13^C-light fractions were respectively combined prior to metagenomic sequencing (see Methods).

**
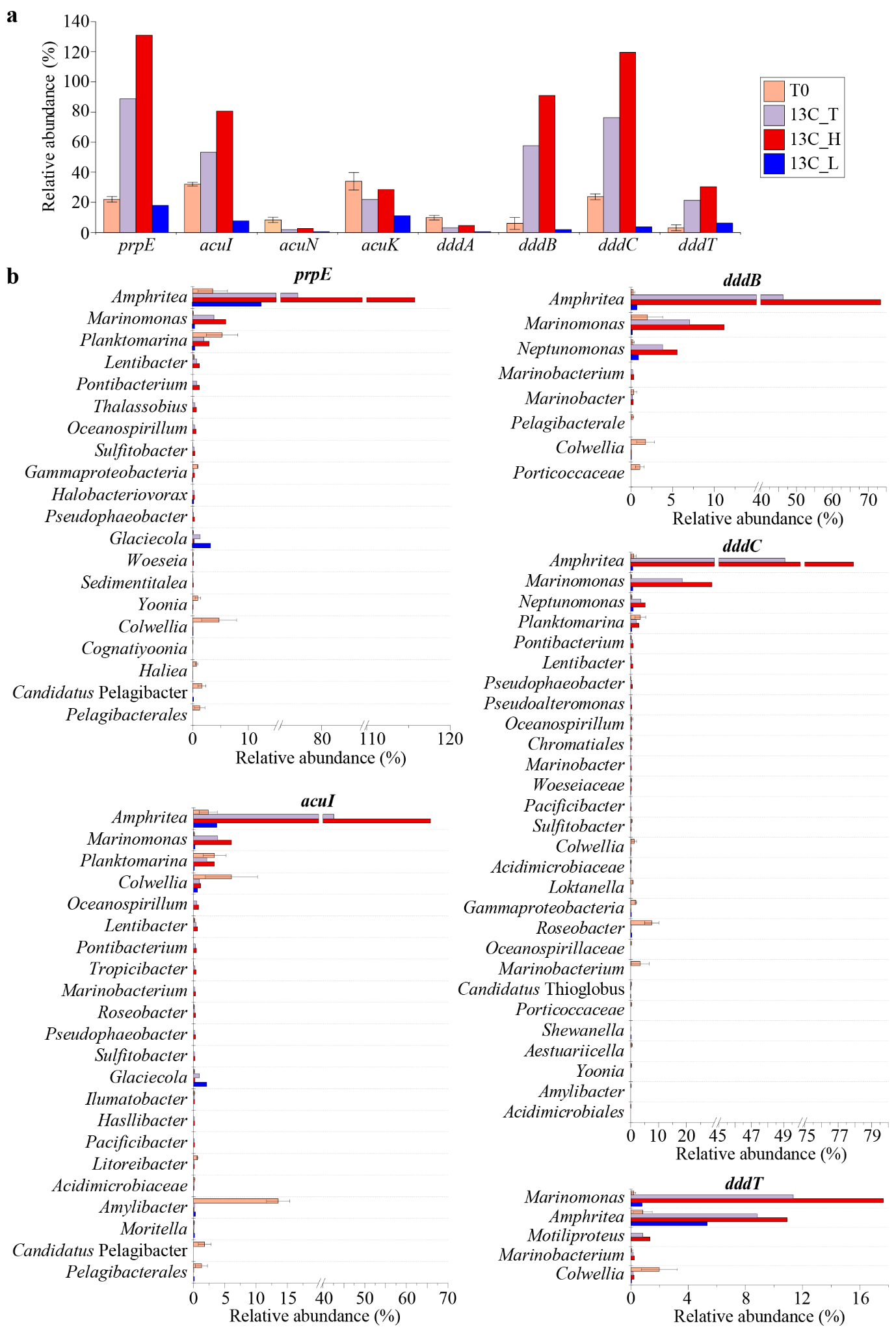
**

**Fig. S10 Relative abundance and taxonomic affiliation of ancillary genes from the DMSP cleavage pathway in coastal seawater metagenomes.** **(a)** Relative abundance (RA) of ancillary genes involved in the lysis of DMSP in seawater metagenomes. **(b)** Taxonomic affiliation of ancillary genes from the DMSP cleavage pathway in seawater metagenomes. T0: metagenomes from natural samples; 13C_T: total microbial community from samples incubated with ^13^C-DMSP. 13C_H: metagenomes from ^13^C-heavy fractions; 13C_L: metagenomes from ^13^C-light fractions. T0 values represent the average of three biological replicates. Biological replicates from ^13^C-heavy and ^13^C-light fractions were respectively combined prior to metagenomic sequencing (see Methods).


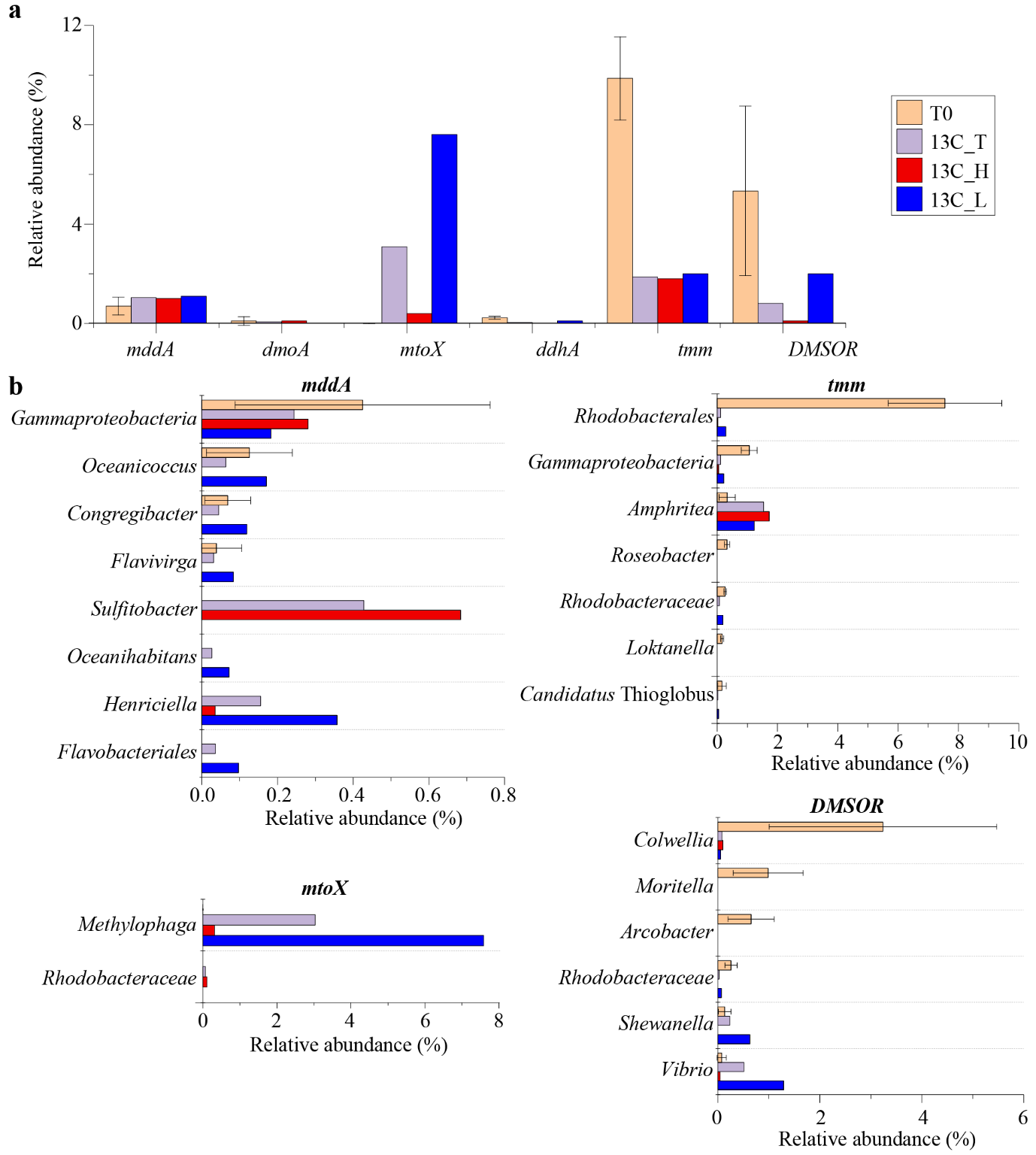


**Fig. S11 Relative abundance and taxonomic affiliation of DMS cycling genes in coastal seawater samples. (a)** Relative abundance (RA) of genes involved in DMS cycling in seawater metagenomes; **(b)** Taxonomic affiliation of DMS cycling genes retrieved from seawater metagenomes. T0: metagenomes from natural samples; 13C_T: total microbial community from samples incubated with ^13^C-DMSP. 13C_H: metagenomes from ^13^C-heavy fractions; 13C_L: metagenomes from ^13^C-light fractions. T0 values represent the average of three biological replicates. Biological replicates from ^13^C-heavy and ^13^C-light fractions were respectively combined prior to metagenomic sequencing (see Methods).

**Table S1.** Relative abundance of genes encoding proteins involved in the cycling of DMSP, DMS and related compounds in metagenomes from seawater samples.

| **Protein**  **product** | **Polypeptide class** | **Reaction catalyzed** | **T0_1** | **T0_2** | **T0_3** | **T0_ave** | **13C_T** | **13C_H** | **13C_L** |
| --- | --- | --- | --- | --- | --- | --- | --- | --- | --- |
| **DSYB** | SAM-dependent methyltransferase | MTHB methylation to DMSHB | 0.2 (7.3) | 0.1 (4.2) | 0.3 (9.7) | 0.2 (7.1) | ND | ND | ND |
| **TpMMT** | SAM-dependent methyltransferase | MTHB methylation to DMSHB | ND | 0.1 (4.5) | ND | 0.03 (1.5) | ND | ND | ND |
| **Alma1** | Aspartic acid/glutamic acid/ethyl urea racemase superfamily | DMSP cleavage to DMS and acrylate | ND | 0.1 (4.3) | 0.6 (18.7) | 0.2 (7.7) | ND | ND | ND |
| **DsyB** | SAM-dependent methyltransferase | MTHB methylation to DMSHB | 0.3 | 0.1 | ND | 0.1 | ND | ND | ND |
| **MmtN** | Met-methylating enzymes | Methionine methylation to SMM | ND | ND | ND | ND | ND | ND | ND |
| **DmdA** | Glycine cleavage system T family | DMSP demethylation to MMPA | 20.3 | 13.5 | 12.9 | 15.6 | 1.8 | 2.4 | 0.9 |
| **DmdB** | AMP combinase superfamily | Catalyze MMPA to MMPA-CoA | 41 | 43.1 | 52.1 | 45.4 | 107.4 | 162.2 | 15.5 |
| **DmdC** | Acyl CoA dehydrogenase superfamily | MMPA-CoA dehydrogenation to  MTA-CoA | 30.6 | 18.3 | 23.7 | 24.2 | 9.5 | 14.1 | 1.8 |
| **DmdD** | Allyl hydrase superfamily | MTA-CoA hydration to acetaldehyde | 7.3 | 4.3 | 4.6 | 5.4 | 1.2 | 1.6 | 0.4 |
| **DddP** | M24B metallopeptidase family | DMSP cleavage to DMS and acrylate | 5.1 | 5.3 | 7 | 5.8 | 0.3 | 0.4 | ND |
| **DddD** | CoA-transferase family III | DMSP cleavage to DMS and 3HP | 0.4 | 1.9 | 2.6 | 1.6 | 60.4 | 95.8 | 1.1 |
| **DddL** | Cupin superfamily | DMSP cleavage to DMS and acrylate | 0.08 | ND | ND | 0.03 | 0.1 | 0.2 | ND |
| **DddQ** | Cupin superfamily | DMSP cleavage to DMS and acrylate | 1.0 | 0.6 | ND | 0.5 | ND | ND | ND |
| **DddW** | Cupin superfamily | DMSP cleavage to DMS and acrylate | ND | ND | ND | ND | 0.2 | 0.3 | ND |
| **DddK** | Cupin superfamily | DMSP cleavage to DMS and acrylate | 0.4 | 0.2 | ND | 0.2 | ND | ND | ND |
| **DddY** | Cupin superfamily | DMSP cleavage to DMS and acrylate | ND | ND | ND | ND | 0.05 | ND | 0.1 |
| **DddX** | Acyl-CoA synthetase superfamily | DMSP cleavage to DMS and acryloyl-CoA | 1.1 | 1.7 | 1.3 | 1.4 | 0.4 | 0.6 | 0.1 |
| **DddA** | GMC oxidoreductase family | 3HP oxidation to MalSA | 10.8 | 8.1 | 10.7 | 9.9 | 3.1 | 4.6 | 0.5 |
| **DddB** | Iron-containing alcohol dehydrogenases-like | 3HP oxidation to MalSA | 1.8 | 6.6 | 9.6 | 6 | 57.6 | 90.9 | 1.9 |
| **DddC** | Methylmalonate-semialdehyde dehydrogenase | MalSA transformation to acetyl CoA | 24.6 | 21.5 | 25.1 | 23.7 | 76.3 | 119.6 | 3.7 |
| **DddT** | BCCT family transporter | DMSP transport | 0.9 | 4 | 4.4 | 3.1 | 21.3 | 30.3 | 6.2 |
| **AcuN** | CoA transferase | Acrylate transformation to 3HP | 7 | 7.7 | 10.3 | 8.3 | 1.8 | 2.6 | 0.5 |
| **AcuK** | Enoyl-CoA hydratase family | Acrylate transformation to 3HP | 40.4 | 29.1 | 32.4 | 34 | 21.9 | 28.4 | 11.1 |
| **PrpE** | Propionate-CoA ligase | Acrylate transformation to acryloyl-CoA | 20.7 | 21.1 | 24.1 | 22 | 88.7 | 131 | 17.9 |
| **AcuI** | Acryloyl-CoA reductase | Acryloyl-CoA reduction to propionyl-CoA | 32.4 | 30.7 | 33.1 | 32.1 | 53.3 | 80.5 | 7.7 |
| **MddA** | Methanethiol S-methyltransferase | Catalyse MeSH to DMS | 1.1 | 0.4 | 0.6 | 0.7 | 1.0 | 1.0 | 1.1 |
| **DmoA** | Dimethylsulfide monooxgenase | DMS transformation to MeSH | 0.3 | ND | ND | 0.1 | 0.08 | 0.1 | ND |
| **MTO** | Methanethiol oxidase | MeSH oxidation to formaldehyde | ND | 0.01 | ND | 0.003 | 3.1 | 0.4 | 7.6 |
| **DdhA** | Dimethylsulfide dehydrogenase | DMS oxidation to DMSO | 0.3 | 0.2 | 0.2 | 0.2 | 0.04 | ND | 0.1 |
| **Tmm** | Trimethylamine monooxygenase | DMS oxidation to DMSO | 11.8 | 8.8 | 9.0 | 9.9 | 1.9 | 1.8 | 2 |
| **DMSOR** | Dimethyl sulfoxide reductase | DMSO reduction to DMS | 1.4 | 7.1 | 7.5 | 5.3 | 0.8 | 0.1 | 2 |

The number of unique hits of genes encoding proteins of interest were normalised to the number of *recA* sequences retrieved from each metagenome. The relative abundance of key eukaryotic DMSP synthesis (*DSYB* and *TpMMT*) and degradation (*Alma1*) genes normalised to *ACTB* is indicated in brackets. HMM searches to retrieve homologous sequences to ratified enzymes of interest were done using a cut-off value of E ≤ 1e-30, except for MddA, MTO, DdhA, Tmm (E ≤ 1e-5) and DMSOR (E ≤ 1e-94). T0_1, T0_2, T0_3, biological replicates from natural (T0) samples; T0_ave, average of biological T0 replicates; 13C_T, total microbial community from seawater incubations with ^13^C-DMSP; 13C_H: heavy fractions from samples incubated with ^13^C-DMSP; 13C_L: light fractions from ^13^C-DMSP incubations. Biological replicates from ^13^C-heavy and ^13^C-light fractions were respectively combined prior to metagenomic sequencing (see Methods). MTHB, 4-methylthio-2-hydroxybutyrate; DMSHB, 4-dimethylsulfonio-2-hydroxybutyrate; SMM, S-methylmethionine. MMPA, methylmercaptopropionate; 3HP, 3‑hydroxypropionate; MalSA, malonate semi-aldehyde; DMS, dimethyl sulfide; MeSH, methanethiol; DMSO, dimethylsulfoxide. ND, not detected.

**Table S2.** Relative abundance (RA) of main bacterial genera from seawater samples analysed by 16S rRNA gene amplicon (16S) and metagenomics (MG) sequencing.

|  |  | T0 | | 13C_H | | 13C_L | | 13C_T | |
| --- | --- | --- | --- | --- | --- | --- | --- | --- | --- |
| Genus | Order | 16S | MG | 16S | MG | 16S | MG | 16S | MG |
| *Colwellia* | *Alteromonadales* | 33.2 ± 21.9 | 7.1 ± 4.7 | 0.7 ± 0.05 ^b^ | 0.2 | 5.3 ± 1.4 | 1.2 | 2.4 ± 0.3 | 0.6 |
| *Glacieola* | *Alteromonadales* | 0.2 ± 0.04 | 0.2 ± 0.05 | 0.3 ± 0.1 ^b^ | 0.1 | 4.4 ± 1.6 | 3.3 | 1.8 ± 0.5 ^c^ | 1.3 |
| *Amylibacter* | *Rhodobacterales* | 13.4 ± 5.1 | ND | 0.1 ± 0.007 ^a^ | ND | 0.3 ± 0.1 | ND | 0.2 ± 0.02 ^c^ | ND |
| *Planktomarina* | *Rhodobacterales* | 5.3 ± 2.4 | 1.5 ± 1.0 | 1.0 ± 0.6 | 0.5 | 0.3 ± 0.1 | 0.1 | 0.7 ± 0.3 | 0.3 |
| *Pseudophaeobacter* | *Rhodobacterales* | 0.2 ± 0.1 | 0.02 ± 0.003 | 1.0 ± 0.4 | 0.04 | 0.01 ± 0.004 | 0.0001 | 0.6 ± 0.2 | 0.03 |
| *Candidatus* Pelagibacter | SAR11 clade | 1.4 ± 1.5 | 1.6 ± 1.4 | 0.01 ± 0.01 | 0.005 | 0.6 ± 0.5 | 0.1 | 0.2 ± 0.2 | 0.04 |
| *Amphritea* | *Oceanospirillales* | 0.7 ± 0.1 | 2.8 ± 1.7 | 52.0 ± 4.3 ^a,b^ | 55.9 | 8.6 ± 4.3 | 7.7 | 36.0 ± 6.6 ^c^ | 37.9 |
| *Marinomonas* | *Oceanospirillales* | 0.7 ± 0.1 | 0.4 ± 0.2 | 25.2 ± 0.5 ^a,b^ | 9.1 | 0.5 ± 0.2 | 0.8 | 16.0 ± 1.1 ^c^ | 6.0 |
| *Marinobacterium* | *Oceanospirillales* | 0.2 ± 0.1 | 0.2 ± 0.1 | 0.6 ± 0.1 ^a,b^ | 1.3 | 0.02 ± 0.01 | 0.2 | 0.4 ± 0.1 ^c^ | 0.9 |
| *Oceanospirillum* | *Oceanospirillales* | 0.01 ± 0.004 | 0.03 ± 0.02 | 1.8 ± 0.1 ^a,b^ | 0.5 | 0.03 ± 0.02 | 0.04 | 1.1 ± 0.1 ^c^ | 0.3 |
| *Halobacteriovorax* | *Bdellovibrionales* | 0.004 ± 0.004 | 0.04 ± 0.01 | 1.0 ± 0.3 ^a,b^ | 0.3 | 0.1 ± 0.05 | 1.0 | 0.7 ± 0.2 ^c^ | 0.6 |
| Unclassified *Bacteriovoracaceae* | *Bdellovibrionales* | 0.1 ± 0.02 | ND | 1.1 ± 0.3 ^a^ | ND | 0.7 ± 0.2 | ND | 1.0 ± 0.1 ^c^ | ND |
| *Vibrio* | *Vibrionales* | 1.1 ± 0.8 | 0.5 ± 0.3 | 0.1 ± 0.1 ^b^ | 0.4 | 13.7 ± 4.7 | 4.2 | 5.0 ± 1.0 ^c^ | 1.9 |
| Unclassified *Piscirickettsiaceae* | *Thiotrichales* | 0.003 ± 0 | ND | 0.5 ± 0.3 ^b^ | ND | 44.5 ± 17.4 | ND | 17.3 ± 8.5 ^c^ | ND |
| *Methylophaga* | *Thiotrichales* | 0.001 ± 0.002 | 0.1 ± 0.03 | 0.01 ± 0.003 ^a,b^ | 0.6 | 1.3 ± 0.4 | 39.3 | 0.5 ± 0.2 ^c^ | 15.1 |

^a^ Statistically significant differences (*P* <0.05) between RAs in 13C_H fractions and T0 samples.

^b^ Statistically significant differences (*P* <0.05) between RAs in 13C_H fractions and 13C_L samples.

^c^ Statistically significant differences (*P* <0.05) between RAs in 13C_T and T0 samples.

ND, not detected.

**Table S3** Dominant bacterial genera in T0 seawater samples analysed by 16S rRNA gene amplicon sequencing.

| **Genus** | **Relative abundance in T0 samples** | **Taxonomy** | **Reference strains (RS)** | **Homologous sequences to ratified DMSP and DMS cycling genes identified in RS** |
| --- | --- | --- | --- | --- |
| *Colwellia* | 33.2 ± 21.8% | *Gammaproteobacteria*; *Alteromonadales* | *Colwellia aestuarii* CGMCC1.6965 | *acuI*, *dmdB*, *dmdC* |
|  |  |  | *Colwellia agarivorans* QM50 | *prpE*, *dmdC* |
|  |  |  | *Colwellia beringensis* NB0971-1 | *dddC*, *acuI*, *dmdB*, *dmdC* |
|  |  |  | *Colwellia chukchiensis* CGMCC 1.9127 | *dmdB*, *dmdC* |
|  |  |  | *Colwellia demingiae* ACAM 459 | *dddD*, *dddB*, *dddC*, *prpE*, *acuI (2)*, *acuN*, *acuK*, *dmdB*, *dmdC* |
|  |  |  | *Colwellia echini* A3 | *acuI*, *dmdC* |
|  |  |  | *Colwellia hornerae* ACAM607 | *acuI*, *dmdB*, *dmdC* |
|  |  |  | *Colwellia marinimaniae* MTCD1 | *dmdC*, *DMSOR* |
|  |  |  | *Colwellia mytili* KCTC 52417 | *dmdB*, *dmdC* |
|  |  |  | *Colwellia piezophila* ATCC BAA-637 | *dddB*, *dddC*, *prpE*, *acuI*, *acuN*, *acuK*, *dmdC* |
|  |  |  | *Colwellia polaris* MCCC 1C0005 | *acuI*, *dmdB*, *dmdC* |
|  |  |  | *Colwellia ponticola* OISW-25 | *prpE*, *acuI*, *dmdB*, *dmdC*, *ddhA* |
|  |  |  | *Colwellia psychrerythraea* 34H | *dddB*, *dddC*, *prpE*, *acuI*, *acuN*, *acuK*, *dmdB*, *dmdC* |
| *Amylibacter* | 13.4 ± 5.1% | *Alphaproteobacteria*; *Rhodobacterales* | *Amylibacter cionae* CGMCC 1.15880 | *prpE*, *acuI*, *acuN*, *acuK dmdA*, *dmdB*, *dmdC*, *dmdD*, *mddA* |
|  |  |  | *Amylibacter kogurei* 4G11 | *dddP*, *dddA*, *prpE*, *acuI*, a*cuK*, *dmdB*, *dmdC* |
|  |  |  | *Amylibacter ulvae* KTCT 32465 | *dddP*, *dddA*, *prpE*, *acuI*, *acuK*, *dmdB*, *dmdC* |
| *Planktomarina* | 5.3 ± 2.1% | *Alphaproteobacteria*; *Rhodobacterales* | *Planktomarina temperata* | *dddP*, *dddC*, *prpE*, *dmdC* |
|  |  |  | *Planktomarina temperata* RCA23 | *dddP*, *dddA*, *dddC*, *acuI*, *prpE*, *acuK*, *dmdB*, *dmdC* |
| *Arcobacter* | 3.0 ± 1.5% | *Epsilonproteobacteria*; *Campylobacterales* | *Arcobacter aquimarinus* W63 | ND |
|  |  |  | *Arcobacter cloacae* LMG 26153 | ND |
|  |  |  | *Arcobacter defluvii* LMG 25694 | ND |
|  |  |  | *Arcobacter ellisii* LMG 26155 | ND |
|  |  |  | *Arcobacter nitrofigilis* DSM 7299 | *dddY*, *DMSOR* |
|  |  |  | *Arcobacter peruensis* PSE-93 | ND |
|  |  |  | *Arcobacter suis* CECT 7833 | ND |
|  |  |  | *Arcobacter venerupis* LMG 26156 | ND |
| *Moritella* | 2.2 ± 1.6% | *Gammaproteobacteria*; *Alteromonadales* | *Moritella dasanensis* ArB 0140 | *dddC*, *acuI* |
|  |  |  | *Moritella marina* ATCC 15381 | *acuI* |
|  |  |  | *Moritella viscosa* | *acuI* |
|  |  |  | *Moritella yayanosii* DB21MT 5 | *acuI* |
| *Loktanella* | 1.8 ± 0.8% | *Alphaproteobacteria*; *Rhodobacterales* | *Loktanella atrilutea* DSM 29326 | *dddA*, *dddC*, *prpE*, *acuI*, *acuK*, *dmdA*, *dmdB*, *dmdC* |
|  |  |  | *Loktanella fryxellensis* DSM 16213 | *dddA*, *dddC*, *prpE*, *acuI*, *acuK*, *dmdB*, *dmdC* |
|  |  |  | *Loktanella ponticola* DSM 101064 | *dddA*, *dddC*, *prpE*, *acuI*, *acuK*, *dmdB*, *dmdC* |
| *Sulfurimonas* | 1.3 ± 1.0% | *Epsilonproteobacteria*; *Campylobacterales* | *Sulfurimonas autotrophica* DSM 16294 | ND |
|  |  |  | *Sulfurimonas crateris* SN118 | ND |
|  |  |  | *Sulfurimonas denitrificans* DSM 1251 | ND |
|  |  |  | *Sulfurimonas gotlandica* GD1 | ND |
| *Pseudoalteromonas* | 1.3 ± 1.2% | *Gammaproteobacteria*; *Alteromonadales;* | *Pseudoalteromonas agarivorans* Hap2018 | *prpE*, *acuI* |
|  |  |  | *Pseudoalteromonas atlantica* NBRC 103033 | *prpE*, *acuI* |
|  |  |  | *Pseudoalteromonas aurantia* 208 | ND |
|  |  |  | *Pseudoalteromonas carrageenovora* KCTC 22325 | *prpE*, *acuI* |
|  |  |  | *Pseudoalteromonas denitrificans* DSM 6059 | *dmdC*, *mtoX* |
|  |  |  | *Pseudoalteromonas flavipulchra* LMG 20361 | *prpE* |
|  |  |  | *Pseudoalteromonas haloplanktis* ATCC 14393 | *acuI* |
|  |  |  | *Pseudoalteromonas lipolytica* CSB02KR | *prpE*, *acuI* |
|  |  |  | *Pseudoalteromonas luteoviolacea* H2 | ND |
|  |  |  | *Pseudoalteromonas paragorgicola* KMM 3548 | *prpE*, *acuI* |
|  |  |  | *Pseudoalteromonas peptidolytica* DSM 14001 | *prpE*, *acuI* |
| *Vibrio* | 1.1 ± 0.8% | *Gammaproteobacteria*; *Cellvibrionales* | *Vibrio campbellii* DS40M4 | *acuI* |
|  |  |  | *Vibrio cholerae* MS6 | *prpE*, *acuI* |
|  |  |  | *Vibrio crassostreae* 16SF1_51 | *prpE*, *acuI*, *DMSOR* |
|  |  |  | *Vibrio cyclitrophicus* ECSME14105 | *prpE*, *acuI*, *DMSOR* |
|  |  |  | *Vibrio harveyi* ATCC 33843 | *prpE*, *acuI*, *DMSOR* |
|  |  |  | *Vibrio mimicus* MB451 | *prpE*, *acuI* |
|  |  |  | *Vibrio nigripulchritudo* Pon4 | *dddC*, *prpE*, *acuI*, *acuK* |
|  |  |  | *Vibrio parahaemolyticus* VPD14 | *prpE*, *acuI*, *DMSOR* |
|  |  |  | *Vibrio splendidus* ZS_90 | *prpE*, *acuI*, *DMSOR* |
|  |  |  | *Vibrio tasmaniensis* LMG 20012 | *prpE*, *acuI* |
| *Marinomonas* | 0.7 ± 0.1% | *Gammaproteobacteria*; *Oceanospirillales* | *Marinomonas algicola* SM1966 | *dddP*, *dddD*, *dddB*, *dddC*, *dmdC* |
|  |  |  | *Marinomonas arctica* BSI20414 | *dddC*, *acuI*, *dmdC* |
|  |  |  | *Marinomonas colpomeniae* SM2066 | *dddC*, *acuI*, *tmm* |
|  |  |  | *Marinomonas foliarum* CECT 7731 | *dddC*, *acuI*, *dmdC* |
|  |  |  | *Marinomonas mediterranea* MMB-1 | *dddD*, *dddB*, *dddC*, *acuI*, *dmdC* |
|  |  |  | *Marinomonas polaris* DSM 16579 | *dddC*, *acuI*, *dmdB*, *dmdC* |
|  |  |  | *Marinomonas posidonica* IVIA-Po-181 | *dddD*, *dddB*, *dddC*, *acuI*, *acuN*, *acuK*, *dmdC* |
|  |  |  | *Marinomonas ushuaiensis* DSM 15871 | *dddP*, *acuI*, *dmdC* |
| *Amphritea* | 0.7 ± 0.1% | *Gammaproteobacteria*; *Oceanospirillales* | *Amphritea atlantica* DSM 18887 | *dddD*, *dddB*, *dddC*, *dddT*, *prpE* (2), *acuI*, *acuK*, *dmdB*, *dmdC*, *tmm* |
|  |  |  | *Amphritea balenae* JAMM1525 | *dddC*, *prpE* (2), *acuI*, *acuK*, *dmdB*, *dmdC* |
|  |  |  | *Amphritea balenae* JCM 14781 | *dddC*, *prpE* (2), *acuK*, *dmdB*, *dmdC* |
|  |  |  | *Amphritea japonica* ATCC BAA-1530 | *dddC*, *prpE* (2), *acuI*, *acuK*,*dmdB*, *dmdC* |
|  |  |  | *Amphritea opalescens* ANRC-JH13 | *dddC*, *dddT*, *prpE* (2), *acuI (2)*, *dmdB*, *dmdC*, *tmm* |
|  |  |  | *Amphritea* sp. RP18W | *dddC*, *prpE* (2), *acuI*, *acuK*, *dmdB*, *dmdC*, *tmm* |
|  |  |  | *Amphritea* sp. ZJ14W | *dddC*, *prpE* (2), *acuI*, *acuK*, *dmdB*, *dmdC*, *tmm* |
|  |  |  | *Amphritea spongicola* JCM16668 | *dddP*, *dddA*, *dddC*, *dddT*, *prpE* (2), *acuI*, *acuK*, *dmdB*, *dmdC* |
| *Tenacibaculum* | 0.6 ± 0.1% | *Flavobacteriia*; *Flavobacteriales* | *Tenacibaculum adriaticum* DSM 18961 | ND |
|  |  |  | *Tenacibaculum aiptasiae* a4 | ND |
|  |  |  | *Tenacibaculum discolor* DSM 18842 | ND |
|  |  |  | *Tenacibaculum finnmarkense* TNO010 | ND |
| *Lentibacter* | 0.6 ± 0.2% | *Alphaproteobacteria*; *Rhodobacterales* | *Lentibacter algarum* DSM 24677 | *dddP*, *prpE*, *acuI*, *acuK*, *dddA*, *dddC*, *dmdA*, *dmdB*, *dmdC*, *tmm* |

Relative abundance values represent the average of three biological replicates. Representative strains of each genus with a publicly available genome were selected as reference strains (RS). Genomes of RS were screened for genes encoding homologous proteins to ratified DMSP and DMS cycling enzymes described in Table S9 by BLASTp using a threshold of E ≤ 1e-30, ≥50% amino acid identity and ≥70% coverage. Number of *prpE* and *dmdB* genes in genomes with multiple copies are indicated in brackets. ND, not detected.

**Table S4.** Relative abundance (RA) of main bacterial orders from seawater samples analysed by 16S rRNA gene amplicon (16S) and metagenomics (MG) sequencing.

|  |  | T0 | | 13C_H | | 13C_L | | 13C_T | |
| --- | --- | --- | --- | --- | --- | --- | --- | --- | --- |
| Order | Class | 16S | MG | 16S | MG | 16S | MG | 16S | MG |
| *Alteromonadales* | *Gammaproteobacteria* | 37.8 ± 24.5% | 16.1 ± 11.6% | 1.3 ± 0.2% | 1.1% | 12.5 ± 3.8% | 8.3% | 5.3 ± 0.9% | 3.8% |
| *Rhodobacterales* | *Alphaproteobacteria* | 22.8 ± 8.3% | 19.6 ± 6.6% | 4.4 ± 1.9% ^b^ | 4.1% | 0.8 ± 0.2% | 0.9% | 3.0 ± 1.0% | 2.9% |
| SAR11 clade | *Alphaproteobacteria* | 1.9% ± 1.8% | 1.9 ± 1.6 | 0.009 ± 0.02% | 0.01% | 0.7 ± 0.7% | 0.1% | 0.3 ± 0.3% | 0.1% |
| *Oceanospirillales* | *Gammaproteobacteria* | 5.3 ± 3.0% | 4.3 ± 2.5% | 81.2 ± 3.5% ^a^ | 70.2% | 9.9 ± 4.8% | 9.7% | 54.8 ± 7.7% ^c^ | 47.6% |
| *Bdellovibrionales* | *Deltaproteobacteria* | 0.2 ± 0.1% | 0.05 ± 0.02% | 5.0 ± 1.2% ^a,b^ | 0.3% | 1.1 ± 0.1% | 1.0% | 3.6 ± 0.8% ^c^ | 0.6% |
| *Vibrionales* | *Gammaproteobacteria* | 1.9 ± 1.2% | 0.9 ± 0.5% | 0.1 ± 0.1% ^b^ | 0.6% | 14.1 ± 4.8% | 5.1% | 5.1 ± 1.0% ^c^ | 2.3% |
| *Thiotrichales* | *Gammaproteobacteria* | 0.5 ± 0.3% | 0.4 ± 0.1% | 0.6 ± 0.3% ^b^ | 0.7% | 45.9 ± 17.5% | 40.5% | 17.8 ± 8.6% | 15.6% |
| *Pseudomonadales* | *Gammaproteobacteria* | 0.01 ± 0.01% | 0.3 ± 0.05% | 0.01 ± 0.003% | 0.4% | 0.001 ± 0.002% | 0.3% | 0.004 ± 0.002% | 0.3% |

^a^ Statistically significant differences (*P* <0.05) between RAs in 13C_H fractions and T0 samples.

^b^ Statistically significant differences (*P* <0.05) between RAs in 13C_H fractions and 13C_L samples.

^c^ Statistically significant differences (*P* <0.05) between RAs in 13C_T and T0 samples.

**Table S5** Metagenome-assembled genomes (MAGs) with homologous sequences to genes involved in DMSP cycling reconstructed from metagenomes from seawater samples.

| **MAGs** | **Completeness (%)^a^** | **Contamination (%)^a^** | **Size (Mbp)** | **Homologous genes** | **Relative abundance (%)** | | | | | | | **Taxonomy** | **Accession number** |
| --- | --- | --- | --- | --- | --- | --- | --- | --- | --- | --- | --- | --- | --- |
|  |  |  |  |  | **T0_1** | **T0_2** | **T0_3** | **T0_ave** | **13C_T** | **13C_H** | **13C_L** |  |  |
| 3 | 96.7 | 0.5 | 3.1 | *dddP*, *prpE*, *acuI*, *acuK*, *dddC*, *dmdA*, *dmdB*, *dmdC*, *tmm* | 0.3 | 0.121 | 0.125 | 0.179 | 0.007 | 0.011 | 0.002 | *Rhodobacterales*; *Loktanella* | JAIBDS000000000 |
| 7 | 76.4 | 3.5 | 1.8 | *dddP*, *dddC*, *dmdA*, *dmdB* (2), *tmm* | 0.4 | 0.2 | 0.198 | 0.3 | 0.071 | 0.01 | 0.171 | *Rhodobacterales*; *Amylibacter* | JAIBDB000000000 |
| 10 | 77.8 | 4.1 | 3 | *acuK* | 0.001 | 0.001 | 0.001 | 0.001 | 0.117 | 0.105 | 0.139 | *Bacteriovoracales*; *Bacteriovorax* | JAIBDC000000000 |
| 11 | 73.9 | 3 | 2.8 | *dddP*, *dmdA*, *prpE*, *dmdB* (2) | 0.17 | 0.087 | 0.061 | 0.106 | 0.054 | 0.009 | 0.129 | *Gammaproteobacteria* | JAIBDK000000000 |
| 12 | 94.5 | 0.6 | 4 | *prpE*, *acuI*, *dmdB* | 0.124 | 0.05 | 0.031 | 0.068 | 0.018 | 0.001 | 0.046 | *Desulfobacterales*; *Desulfofustis* | JAIBDJ000000000 |
| 13 | 56.8 | 1 | 1.4 | *dsyB*, *dddA*, *acuK*, *dmdB* | 0.086 | 0.04 | 0.036 | 0.054 | 0.011 | 0.003 | 0.023 | *Betaproteobacteria* | JAIBDE000000000 |
| 17 | 53.4 | 4.3 | 2.8 | *dmdB*, *dmdC* | 0.033 | 0.18 | 0.3 | 0.157 | 0.107 | 0.019 | 0.3 | *Alteromonadales*; *Colwellia* | JAIBDG000000000 |
| 18 | 72.5 | 2.4 | 2 | *dddP*, *dddC*, *prpE*, *dmdA*, *dmdC* | 0.3 | 0.094 | 0.087 | 0.151 | 0.016 | 0.003 | 0.039 | *Rhodobacterales*; *Rhodobacteraceae* | JAIBEE000000000 |
| 21 | 58.6 | 0 | 1.6 | *mtoX* | 0.005 | 0.012 | 0.013 | 0.01 | 1.5 | 0.116 | 4 | *Thiotrichales*; *Piscirickettsiaceae* | JAIBDZ000000000 |
| 23 | 74.6 | 3.2 | 2.3 | *dddD*, *dddB*, *dddC*, *prpE*, *acuI*, *dmdB* (3), *dmdC* | 0.3 | 0.7 | 1.2 | 0.7 | 35.3 | 53.6 | 4.6 | *Oceanospirillales*; *Amphritea* | JAIBCZ000000000 |
| 26 | 59.1 | 3.5 | 4.1 | *acuI*, *acuK*, *dmdC*, *mtoX, DMSOR* | 0.004 | 0.026 | 0.038 | 0.023 | 0.056 | 0.008 | 0.137 | *Alteromonadales*; *Shewanella* | JAIBEH000000000 |
| 29 | 71.7 | 1.1 | 2.2 | *dddC*, *prpE*, *acuK dmdB*, *dmdC* | 0.15 | 0.047 | 0.036 | 0.078 | 0.001 | 0.002 | 0.001 | *Rhodobacterales* | JAIBEG000000000 |
| 30 | 61.4 | 3.2 | 2 | *dddB*, *dddC*, *dddT*, *dmdB* | 0.003 | 0.006 | 0.007 | 0.005 | 0.4 | 0.6 | 0.023 | *Oceanospirillales*; *Oceanospirillum* | JAIBDX000000000 |
| 32 | 86.5 | 4.9 | 3 | *mtoX* | 0.5 | 0.166 | 0.156 | 0.3 | 0.159 | 0.2 | 0.074 | *Gammaproteobacteria* | JAIBDL000000000 |
| 35 | 77.7 | 1.4 | 1.9 | *dmdC* | 0.081 | 0.038 | 0.04 | 0.053 | 0.023 | 0.015 | 0.038 | *Gammaproteobacteria* | JAIBDM000000000 |
| 36 | 59.1 | 0 | 3 | *dddP*, *dddD*, *acuI*, *dddB*, *dddC*, *dmdB*, *dmdC*, *tmm* | 0.01 | 0.013 | 0.019 | 0.014 | 0.152 | 0.2 | 0.012 | *Oceanospirillales*; *Neptuniibacter* | JAIBDV000000000 |
| 41 | 50.7 | 1.7 | 1.3 | *dmdB* (2), *dmdD* | 0.3 | 0.118 | 0.119 | 0.165 | 0.043 | 0.048 | 0.035 | *Cellvibrionales*; *Porticoccaceae* | JAIBEB000000000 |
| 43 | 84.5 | 2.7 | 3.3 | *acuK* | 0.001 | 0.002 | 0.002 | 0.002 | 0.108 | 0.043 | 0.2 | *Bacteriovoracales*; *Halobacteriovorax* | JAIBDQ000000000 |
| 44 | 94.6 | 0.2 | 3 | *dddP*, *dddW*, *prpE*, *acuI*, *acuK*, *dddC*, *dmdA*, *dmdB* (3), *dmdC* | 0.7 | 0.4 | 0.4 | 0.5 | 0.071 | 0.095 | 0.032 | *Rhodobacterales*; *Loktanella* | JAIBDT000000000 |
| 45 | 68.7 | 0.5 | 1.6 | *dmdB*, *acuK* | 0.111 | 0.039 | 0.023 | 0.058 | 0.022 | 0.016 | 0.031 | *Rhizobiales*; *Methyloceanibacter* | JAIBDU000000000 |
| 55 | 55.2 | 0.5 | 1.1 | *dmdA*, *dmdB* (2), *tmm* | 7.6 | 3.8 | 3.8 | 5.1 | 0.4 | 0.164 | 0.7 | *Alphaproteobacteria* | JAIBCY000000000 |
| 58 | 75.1 | 2.5 | 2.5 | *dmdA*, *dmdB, dmdC*, *mtoX* | 0.2 | 0.089 | 0.061 | 0.126 | 0.028 | 0.006 | 0.065 | *Gammaproteobacteria* | JAIBDN000000000 |
| 60 | 94.5 | 1.3 | 2.1 | *dddP*, *dddC*, *prpE*, *acuI*, *dmdA*, *dmdC* | 0.192 | 0.148 | 0.165 | 0.168 | 0.003 | 0.003 | 0.003 | *Oceanospirillales*; *Oceanospirillaceae* | JAIBDW000000000 |
| 61 | 59.5 | 2.6 | 3.2 | *dmdB* (2), *dmdC* | 0.078 | 1 | 1.3 | 0.8 | 0.041 | 0.015 | 0.084 | *Alteromonadales*; *Colwellia* | JAIBDH000000000 |
| 63 | 95.6 | 0 | 2.6 | *acuK* | 0.2 | 0.086 | 0.107 | 0.132 | 0.006 | 0.004 | 0.01 | *Bacteroidetes* | JAIBDD000000000 |
| 65 | 66.4 | 0.9 | 7.1 | *dmdC* | 0.001 | 0.001 | 0.001 | 0.001 | 0.114 | 0.18 | 0.002 | *Deltaproteobacteria* | JAIBDI000000000 |
| 68 | 62.6 | 0 | 3.5 | *dddA*, *dddC*, *prpE*, *acuI*, *acuN*, *dmdA*, *dmdB* (3), *dmdC*, *dmdD* | 0.027 | 0.019 | 0.018 | 0.022 | 0.179 | 0.3 | 0.004 | *Rhodobacterales*; *Pseudophaeobacter* | JAIBED000000000 |
| 72 | 72.2 | 3.4 | 2.4 | *dddC*, *prpE*, *acuK*, *dmdB* (2), *dmdC* | 0.2 | 0.118 | 0.123 | 0.156 | 0.007 | 0.008 | 0.005 | *Rhodobacterales*; *Rhodobacteraceae* | JAIBEF000000000 |
| 73 | 83.9 | 1.5 | 2.2 | *dddP*, *dmdC* | 5 | 1.7 | 1.7 | 2.8 | 1.4 | 2 | 0.3 | *Rhodobacterales*; *Planktomarina temperata* | JAIBEA000000000 |
| 75 | 70.7 | 0 | 2.8 | *dddP*, *prpE*, *acuI*, *acuK*, *dddC*, *dmdB, dmdC*, *tmm* | 0.3 | 0.4 | 0.3 | 0.3 | 0.013 | 0.013 | 0.013 | *Gammaproteobacteria* | JAIBDO000000000 |
| 77 | 90 | 3.2 | 3.5 | *prpE*, *dmdB*, *dmdC* | 0.4 | 0.151 | 0.101 | 0.2 | 0.146 | 0.2 | 0.038 | *Chromatiales* | JAIBDF000000000 |
| 80 | 60.5 | 1.7 | 5.8 | *dmdC* | 0.002 | 0.001 | 0.001 | 0.001 | 0.106 | 0.168 | 0.002 | *Proteobacteria* | JAIBEC000000000 |
| 81 | 95.7 | 2.2 | 2.7 | *dddP*, *prpE*, *acuK*, *dddA*, *dddC*, *dmdA*, *dmdB* (2), *dmdC* | 0.2 | 0.093 | 0.097 | 0.132 | 0.5 | 0.7 | 0.019 | *Rhodobacterales*; *Lentibacter algarum* | JAIBDR000000000 |
| 83 | 80.1 | 2.2 | 2.1 | *prpE*, *acuI*, *dmdC* | 0.06 | 0.066 | 0.091 | 0.072 | 1.2 | 0.158 | 3 | *Alteromonadales*; *Glaciecola* | JAIBDP000000000 |
| 85 | 51.7 | 0 | 1.3 | *dddT*, *acuI*, *dmdC* | 0.136 | 0.8 | 0.9 | 0.6 | 0.9 | 1.3 | 0.4 | *Oceanospirillales*; *Amphritea* | JAIBDA000000000 |

MAGs were reconstructed from seawater metagenomes using MetaBAT2 [2] and their completeness and contamination were assessed with CheckM [3]. Resultant MAGs were screened for the presence of genes encoding homologous proteins to the ratified DMSP cycling enzymes described in Table S9 using a threshold of E ≤ 1e-30, ≥50% amino acid identity and ≥70% coverage. Number of *prpE* and *dmdB* genes in genomes with multiple copies are indicated in brackets. T0_1, T0_2, T0_3, biological replicates from natural (T0) samples; T0_ave, average of biological T0 replicates; 13C_T, total microbial community from seawater incubations with ^13^C-DMSP; 13C_H: heavy fractions from samples incubated with ^13^C-DMSP; 13C_L: light fractions from ^13^C-DMSP incubations. Biological replicates from ^13^C-heavy and ^13^C-light fractions were respectively combined prior to metagenomic sequencing (see Methods).

**Table S6** Characteristics of bacterial strains with DMSP-degrading activity isolated from seawater incubations with DMSP.

| **Strains** | **Isolation medium** | **DMS or MeSH production** | **Top-hit taxon** | **Top-hit strain** | **16S rRNA gene identity (%)** | **Top-hit taxonomy** | **DMS production rate** | **Grow on DMSP** |
| --- | --- | --- | --- | --- | --- | --- | --- | --- |
| GY12 | MC | MeSH | *Litoreibacter albidus* | DSM 26922 | 98.1 | *Alphaproteobacteria;Rhodobacterales;Rhodobacteraceae* | ND | N |
| MB12-2 | MB | DMS | *Neptunicoccus sediminis* | CY02 | 99.1 | *Alphaproteobacteria;Rhodobacterales;Rhodobacteraceae* | 6 ± 2 | N |
| MB13-2 | MB | DMS | *Neptunicoccus sediminis* | CY03 | 98.9 | *Alphaproteobacteria;Rhodobacterales;Rhodobacteraceae* | 13 ± 0.5 | N |
| MB13-6 | MB | DMS | *Neptunicoccus sediminis* | CY04 | 99.5 | *Alphaproteobacteria;Rhodobacterales;Rhodobacteraceae* | 20 ± 3 | Y |
| GY7 | MC | DMS | *Pseudophaeobacter arcticus* | DSM 23566 | 98.9 | *Alphaproteobacteria;Rhodobacterales;Rhodobacteraceae* | 556 ± 260 | N |
| MB12-4 | MB | DMS | *Sulfitobacter pontiacus* | DSM 10014 | 98.4 | *Alphaproteobacteria;Rhodobacterales;Rhodobacteraceae* | 648 ± 153 | N |
| MB13-11 | MB | DMS | *Sulfitobacter pontiacus* | DSM 10014 | 97.7 | *Alphaproteobacteria;Rhodobacterales;Rhodobacteraceae* | 831 ± 62 | N |
| MB13-9 | MB | DMS | *Sulfitobacter pontiacus* | DSM 10014 | 97.7 | *Alphaproteobacteria;Rhodobacterales;Rhodobacteraceae* | 980 ± 74 | N |
| MC12-2 | MC | DMS | *Sulfitobacter pontiacus* | DSM 10014 | 97.3 | *Alphaproteobacteria;Rhodobacterales;Rhodobacteraceae* | 423 ± 22 | N |
| MC12-6 | MC | DMS | *Sulfitobacter pontiacus* | DSM 10014 | 98.2 | *Alphaproteobacteria;Rhodobacterales;Rhodobacteraceae* | 1411 ± 27 | Y |
| GY16 | MC | DMS | *Sulfitobacter pseudonitzschiae* | H3 | 97.4 | *Alphaproteobacteria;Rhodobacterales;Rhodobacteraceae* | 201 ± 40 | N |
| GY24 | MC | DMS | *Sulfitobacter pseudonitzschiae* | H3 | 99.6 | *Alphaproteobacteria;Rhodobacterales;Rhodobacteracea* | 133 ± 29 | N |
| D12-10 | MD | DMS | *Alteromonas stellipolaris* | LMG 21861 | 98.6 | *Gammaproteobacteria;Alteromonadales;Alteromonadaceae* | 60 ± 6 | Y |
| D12-11 | MD | DMS | *Alteromonas stellipolaris* | LMG 21861 | 98.8 | *Gammaproteobacteria;Alteromonadales;Alteromonadaceae* | 1054 ± 89 | Y |
| **GY8** | **MC** | **DMS** | ***Marinobacter sediminum*** | **R65** | **96.5** | ***Gammaproteobacteria;Alteromonadales;Marinobacter_f*** | **4560 ± 785** | **Y** |
| MC12-9 | MC | DMS | *Marinobacter similis* | A3d10 | 97.9 | *Gammaproteobacteria;Alteromonadales;Marinobacter_f* | 86 ± 3 | Y |
| GY4 | MC | DMS | *Marinobacter similis* | A3d10 | 98.5 | *Gammaproteobacteria;Alteromonadales;Marinobacter_f* | 706 ± 70 | Y |
| GY20 | MC | MeSH | *Pseudoalteromonas hodoensis* | H7 | 99 | *Gammaproteobacteria;Alteromonadales;Pseudoalteromonadaceae* | ND | N |
| D13-2 | MD | DMS | *Cobetia amphilecti* | KMM 1561 | 99.4 | *Gammaproteobacteria;Oceanospirillales;Halomonadaceae* | 1341 ± 101 | N |
| GY9 | MC | DMS | *Cobetia amphilecti* | KMM 1561 | 98.9 | *Gammaproteobacteria;Oceanospirillales;Halomonadaceae* | 106 ± 55 | N |
| **MC13-5** | **MC** | **DMS** | ***Cobetia litoralis*** | **KMM 3880** | **98.9** | ***Gammaproteobacteria;Oceanospirillales;Halomonadaceae*** | **1316 ± 94** | **Y** |
| **GY6** | **MC** | **DMS** | ***Amphritea atlantica*** | **DSM 18887** | **97.9** | ***Gammaproteobacteria;Oceanospirillales;Oceanospirillaceae*** | **1173 ± 208** | **Y** |
| **D13-1** | **MD** | **DMS** | ***Marinobacterium rhizophilum*** | **CL-YJ9** | **99.3** | ***Gammaproteobacteria;Oceanospirillales;Oceanospirillaceae*** | **1039 ± 21** | **Y** |
| MC12-12 | MC | DMS | *Marinobacterium rhizophilum* | CL-YJ9 | 98.3 | *Gammaproteobacteria;Oceanospirillales;Oceanospirillaceae* | 1084 ± 144 | Y |
| MC13-1 | MC | DMS | *Marinobacterium rhizophilum* | CL-YJ9 | 99.7 | *Gammaproteobacteria;Oceanospirillales;Oceanospirillaceae* | 3151 ± 72 | Y |
| MC13-2 | MC | DMS | *Marinobacterium rhizophilum* | CL-YJ9 | 98.7 | *Gammaproteobacteria;Oceanospirillales;Oceanospirillaceae* | 1090 ± 123 | Y |
| MC13-7 | MC | DMS | *Marinobacterium profundum* | PAMC 27536 | 94.3 | *Gammaproteobacteria;Oceanospirillales;Oceanospirillaceae* | 1132 ± 31 | Y |
| MC12-1 | MC | DMS | *Marinobacterium profundum* | PAMC 27536 | 99.4 | *Gammaproteobacteria;Oceanospirillales;Oceanospirillaceae* | 1357 ± 13 | Y |
| GY10 | MC | DMS | *Marinobacterium profundum* | PAMC 27536 | 99.8 | *Gammaproteobacteria;Oceanospirillales;Oceanospirillaceae* | 254 ± 13 | Y |
| GY14 | MC | DMS | *Marinobacterium profundum* | PAMC 27536 | 99.9 | *Gammaproteobacteria;Oceanospirillales;Oceanospirillaceae* | 275 ± 53 | Y |
| GY1 | MC | DMS | *Marinomonas atlantica* | Cmf 18.22 | 99.6 | *Gammaproteobacteria;Oceanospirillales;Oceanospirillaceae* | 60 ± 30 | Y |
| MB12-3 | MB | DMS | *Marinomonas foliarum* | IVIA-Po-155 | 99.2 | *Gammaproteobacteria;Oceanospirillales;Oceanospirillaceae* | 11 ± 0.4 | N |
| MC12-5 | MC | DMS | *Marinomonas foliarum* | IVIA-Po-155 | 99.2 | *Gammaproteobacteria;Oceanospirillales;Oceanospirillaceae* | 18 ± 1 | N |
| MC13-3 | MC | DMS | *Marinomonas foliarum* | IVIA-Po-155 | 99.4 | *Gammaproteobacteria;Oceanospirillales;Oceanospirillaceae* | 15 ± 3 | N |
| MC12-13 | MC | DMS | *Marinomonas foliarum* | IVIA-Po-155 | 99.7 | *Gammaproteobacteria;Oceanospirillales;Oceanospirillaceae* | 6 ± 0.2 | N |
| **MB12-11** | **MB** | **DMS** | ***Marinomonas rhizomae*** | **IVIA-Po-145** | **97.8** | ***Gammaproteobacteria;Oceanospirillales;Oceanospirillaceae*** | **500 ± 36** | **Y** |
| GY13 | MC | DMS | *Marinomonas sp.* | S3726 | 97.7 | *Gammaproteobacteria;Oceanospirillales;Oceanospirillaceae* | 116 ± 8 | N |
| **D13-4** | **MD** | **DMS** | ***Pseudomonas benzenivorans*** | **DSM 8628** | **97.8** | ***Gammaproteobacteria;Pseudomonadales;Pseudomonadaceae*** | **2462 ± 123** | **Y** |
| D13-15 | MD | DMS | *Pseudomonas benzenivorans* | DSM 8628 | 98.1 | *Gammaproteobacteria;Pseudomonadales;Pseudomonadaceae* | 1194 ± 2 | Y |
| GY21 | MC | DMS | *Pseudomonas benzenivorans* | DSM 8628 | 97.5 | *Gammaproteobacteria;Pseudomonadales;Pseudomonadaceae* | 1574 ± 185 | Y |
| MC12-18 | MC | DMS | *Pseudomonas benzenivorans* | DSM 8628 | 97.8 | *Gammaproteobacteria;Pseudomonadales;Pseudomonadaceae* | 2626 ± 45 | N |
| GY22 | MC | DMS | *Pseudomonas leptonychotis* | CCM 8849 | 98.6 | *Gammaproteobacteria;Pseudomonadales;Pseudomonadaceae* | 2350 ± 343 | Y |
| GY17 | MC | DMS | *Pseudomonas taeanensis* | MS-3 | 98.3 | *Gammaproteobacteria;**Pseudomonadales;Pseudomonadaceae* | 830 ± 186 | Y |
| GY15 | MC | MeSH | *Vibrio splendidus* | 10N.286.45 | 99.8 | *Gammaproteobacteria;**Vibrionales;Vibrionaceae* | ND | Y |

Bacterial strains were isolated by plating out serially diluted seawater incubations with DMSP onto marine both agar (MB), MBM with mixed carbon source (MC) or MBM with DMSP as sole carbon source (MD). DMS production rate from DMSP for each isolate is expressed as nmol DMS·mg protein^-1^·h^-1^. Growth on DMSP as sole carbon source was tested in liquid cultures with 2 mM substrate and determined by cell density at OD_600_ (see Methods). Y, good growth on DMSP (*P* < 0.05); N, no growth on DMSP (*P* > 0.05). Isolates whose genomes were sequenced in this study are indicated in bold (accession numbers shown in Table 1).

**Table S7** 16S rRNA gene amplicon sequencing results for coastal seawater samples.

| **Sample** | **Number of quality-filtered reads** | **Base pairs of quality-filtered reads** | **Number of sequences after rarefaction** |
| --- | --- | --- | --- |
| T0_1 | 63205 | 19774403 | 31951 |
| T0_2 | 54121 | 18081927 | 31951 |
| T0_3 | 60975 | 18733593 | 31951 |
| 13C_H_1 | 31951 | 9091529 | 31951 |
| 13C_H_2 | 36811 | 10652765 | 31951 |
| 13C_H_3 | 67541 | 19171963 | 31951 |
| 13C_L_1 | 52037 | 14943153 | 31951 |
| 13C_L_2 | 63545 | 18693432 | 31951 |
| 13C_L_3 | 64589 | 18918911 | 31951 |
| 12C_H_1 | 67333 | 19092959 | 31951 |
| 12C_H_2 | 57189 | 16138214 | 31951 |
| 12C_H_3 | 48924 | 13872534 | 31951 |
| 12C_L_1 | 46536 | 13478286 | 31951 |
| 12C_L_2 | 51471 | 14617463 | 31951 |
| 12C_L_3 | 61123 | 17378013 | 31951 |

DNA extracted from natural (T0) samples, and heavy (H) and light (L) fractions from ^12^C- (12C) and ^13^C-DMSP (13C) incubations was subjected to 16S rRNA gene amplicon sequencing, obtaining an average of 54936 quality-filtered reads per sample with an average length of 273 bp.

**Table S8** Statistics for metagenomic sequencing and assemblies.

| **Sample** | **Quality-filtered data (Gb)** | **Q30 (%)** | **Number of contigs** | **Assembly length (bp)** | **Longest contig (bp)** | **N50 (bp)** | **Mapping rate (%)** |
| --- | --- | --- | --- | --- | --- | --- | --- |
| T0_1 | 13.99 | 90.98 | 963924 | 693850734 | 195659 | 744 | 28.01 |
| T0_2 | 14.05 | 91.40 | 961591 | 612660704 | 120571 | 625 | 24.32 |
| T0_3 | 13.18 | 91.62 | 846370 | 554456321 | 107147 | 653 | 28.38 |
| 13C_H | 14.10 | 91.09 | 570927 | 473468621 | 244645 | 958 | 47.41 |
| 13C_L | 14.07 | 91.59 | 737334 | 574640650 | 575546 | 852 | 39.56 |

Metagenomic reads from natural (T0) seawater samples and ^13^C-heavy (13C_H) and ^13^C-light (13C_L) fractions from incubations with ^13^C-DMSP were quality-assessed and trimmed using SOAPnuke [4]. Quality-filtered reads were then assembled with IDBA_UD [5] and SOAP*denovo2* [6]. Three biological replicates of T0 samples were sequenced separately, whereas biological samples from 13C_H and 13C_L fractions were respectively combined before metagenomic sequencing (see Methods).

**Table S9** Accession numbers of previously ratified enzymes involved in the cycling of DMSP and related compounds.

| **Protein** | **Ratified strains** | **Accession number** | **Reference** |
| --- | --- | --- | --- |
| DSYB | *Prymnesium parvum* CCAP946/6 | NA | [7] |
|  | *Chrysochromulina tobin* CCMP291 | KOO32714 |  |
|  | *Lingulodinium polyedrum* CCMP1936 | NA |  |
|  | *Alexandrium tamarense* ATSP1-B | NA |  |
|  | *Acropora cervicornis* | NA |  |
|  | *Fragilariopsis cylindrus* CCMP1102 | OEU17621 |  |
|  | *Symbiodinium microadriaticum* CCMP2467 | OLQ07620 |  |
| TpMMT | *Thalassiosira pseudonana* CCMP1335 | Tp23128 | [8] |
| DsyB | *Labrenzia aggregata* IAM 12614 | EAV42226 | [9] |
|  | *Pseudooceanicola batsensis* HTCC2597 | EAQ04968 |  |
|  | *Pelagibaca bermudensis* HTCC2601 | EAU45958 |  |
|  | *Amorphus coralli* DSM 19760 | WP_018697905 |  |
| MmtN | *Thalassospira profundimaris* PB8B | OAZ15224 | [10] |
|  | *Novosphingobium* sp. MBES04 | GAM03459 |  |
|  | *Roseovarius indicus* B108 | KRS18724 |  |
|  | *Nocardiopsis chromatogenes* YIM 90109 | WP_017624909 |  |
|  | *Streptomyces mobaraensis* DSM 40847 | EME99407 |  |
| DmdA | *Ruegeria pomeroyi* DSS-3 | AAV95190 | [11] |
|  | *Candidatus* Pelagibacter ubique HTCC1062 | WP_011281570 |  |
|  | *Dinoroseobacter shibae* DFL 12 | WP_012178987 | [12] |
|  | *marine gammaproteobacterium* HTCC2080 | WP_007233625 |  |
|  | *Candidatus* Pelagibacter sp. HTCC7211 | WP_008546106 | [13] |
|  | *Candidatus* Puniceispirillum marinum IMCC1322 | WP_013044947 |  |
| DddD | *Marinomonas* sp. MWYL1 | ABR72937 | [14] |
|  | *Oceanimonas doudoroffii* | AEQ39135 | [15] |
|  | *Psychrobacter* sp. J466 | ACY02894 |  |
|  | *Halomonas* sp. HTNK1 | ACV84065 | [16] |
|  | *Sinorhizobium fredii* NGR234 | AAQ87407 | [14] |
|  | *Burkholderia ambifaria* AMMD | WP_011659284 |  |
|  | *Pseudomonas* sp. J465 | ACY01992 | [17] |
| DddL | *Sulfitobacter* sp. EE-36 | ADK55772 | [18] |
|  | *Rhodobacter sphaeroides* 2.4.1 | YP_351475 |  |
|  | *Labrenzia aggregata* LZB033 | KP639184 | [9] |
|  | *Ahrensia marina* LZD062 | KP639183 | [19] |
| DddP | *Roseovarius nubinhibens* ISM | EAP77700 | [20] |
|  | *Ruegeria pomeroyi* DSS-3 | WP_044029245 | [21] |
|  | *Phaeobacter inhibens* DSM 17395 | AFO91571 | [22] |
|  | *Oceanimonas doudoroffii* DSM 7028 | AEQ39091 | [15] |
|  | *Oceanimonas doudoroffii* DSM 7028 | AEQ39103 |  |
|  | *Aspergillus oryzae* RIB40 | BAE62778 | [20] |
|  | *Fusarium graminearum* PH-1 | XP_389272 |  |
| DddQ | *Ruegeria pomeroyi* DSS-3 | WP_011047333 | [21] |
|  | *Roseovarius nubinhibens* ISM | EAP76002 |  |
|  | *Roseovarius nubinhibens* ISM | EAP76001 |  |
|  | *Ruegeria lacuscaerulensis* ITI1157 | WP_005978225 | [23] |
|  | GOS_2632696 | ECW91654 | [21] |
|  | GOS_7860946 | EBP74803 |  |
|  | GOS_2469775 | ECX82089 |  |
| DddW | *Ruegeria pomeroyi* DSS-3 | AAV93771 | [24] |
| DddY | *Alcaligenes faecalis* M3A | ADT64689 | [25] |
|  | *Desulfovibrio acrylicus* | SHJ73420 | [26] |
|  | *Acinetobacter bereziniae* | ENV21217 | [27] |
|  | *Ferrimonas kyonanensis* DSM 18153 | WP_028114584 | [28] |
|  | *Shewanella putrefaciens* CN-32 | ABP77243 | [29] |
| DddK | *Candidatus* Pelagibacter ubique HTCC1062 | AAZ21215 | [30] |
|  | *Candidatus* Pelagibacter ubique HTCC9022 | WP_028037226 |  |
|  | alphaproteobacterium_HIMB5 | AFS47241.1 |  |
| DddX | *Marinobacterium jannaschii* | WP_084332639.1 | [31] |
|  | *Pelagicola* sp. LXJ1103 | WP_109384856.1 |  |
|  | *Psychrobacter* sp. P11G5 | WP_068035783.1 |  |
|  | *Sporosarcina* sp. P33 | WP_081242855.1 |  |
| Alma1 | *Emiliania huxleyi* CCMP1516 | XP_005784450 | [32] |
|  | *Emiliania huxleyi* CCMP1516 | XP_005763983 |  |
| MddA | *Mycobacterium tuberculosis* H37Rv | WP_003416945.1 | [33] |
|  | *Bradyrhizobium diazoefficiens* USDA 110 | WP_011084036.1 |  |
|  | *Bradyrhizobium diazoefficiens* USDA 110 | WP_011088485.1 |  |
|  | *Pseudomonas* sp. GM41 | WP_008148420.1 |  |
|  | *Pseudomonas deceptionensis* | WP_048359798.1 |  |
|  | *Sulfurovum* sp. NBC37-1 | WP_011980608.1 |  |
| DmoA | *Hyphomicrobium sulfonivorans* 6AK1_A | E9JFX9.1 | [34] |
| MtoX | *Hyphomicrobium* sp. | ATJ26742.1 | [35] |
|  | *Methylophaga thiooxydans* | WP_008290534.1 | [36] |
|  | *Ruegeria pomeroyi* | WP_011242048.1 |  |
| DdhA | *Rhodovulum sulfidophilum* | WP_075783016.1 | [37] |
| Tmm | *Methylocella silvestris* BL2 | ACK52489.1 | [38] |
|  | *Ruegeria pomeroyi* DSS-3 | AAV94838.1 |  |
|  | *Roseovarius* sp. 217 | EAQ26624.1 |  |
| DMSOR | *Rhodobacter capsulatus* 1DMR_A | See the sequence in Song et al., 2020 | [39] |
|  | *Escherichia coli* | WP_097479356.1 | [40] |
|  | *Rhodobacter sphaeroides* | AAB94874.1 | [41] |
| DmdB | *Ruegeria pomeroyi* | WP_011047771.1 | [42] |
|  | *Ruegeria pomeroyi* | WP_011046428.1 |  |
|  | Candidatus *Pelagibacter ubique* | WP_011281571.1 |  |
| DmdC | *Ruegeria pomeroyi* | WP_011049476.1 | [42] |
|  | *Burkholderia thailandensis* E264 | WP_009892931.1 |  |
|  | *Ruegeria pomeroyi* DSS-3 | WP_011048615.1 |  |
|  | *Pseudomonas aeruginosa* PAO1 | WP_003114720.1 |  |
|  | *Pseudomonas aeruginosa* PAO1 | WP_003114561.1 |  |
|  | *Burkholderia thailandensis* E264 | WP_009889880.1 |  |
|  | *Ruegeria lacuscaerulensis* ITI-1157 | EEX08676.1 |  |
| DmdD | *Ruegeria pomeroyi* | Q5LLW6.1 | [42, 43] |
| AcuN | *Halomonas* sp. HTNK1 | ACV84068.1 | [16, 29] |
|  | *Alcaligenes faecalis* | WP_123051137.1 |  |
| AcuK | *Halomonas* sp. HTNK1 | ACV84067.1 | [16, 29] |
|  | *Alcaligenes faecalis* | WP_205649097.1 |  |
| PrpE | *Ruegeria lacuscaerulensis* ITI-1157 | EEX10111.1 | [44, 45] |
|  | *Ruegeria pomeroyi* DSS-3 | AAV96175.1 |  |
| AcuI | *Ruegeria pomeroyi* DSS-3 | WP_011047645.1 | [44] |
|  | *Rhodobacter sphaeroides* | WP_158964488.1 |  |

Ratified proteins were used to confirm sequences obtained from metagenomes, metagenome-assembled genomes (MAGs) and genomes of bacterial strains as functional genes of interest.

**References for Additional File 1**

1. Tamura K, Peterson D, Peterson N, Stecher G, Nei M, Kumar S. MEGA5: molecular evolutionary genetics analysis using maximum likelihood, evolutionary distance, and maximum parsimony methods. Mol Biol Evol. 2011;28:2731–2739.

2. Kang DD, Froula J, Egan R, Wang Z. MetaBAT, an efficient tool for accurately reconstructing single genomes from complex microbial communities. PeerJ. 2015;3:e1165.

3. Parks DH, Imelfort M, Skennerton CT, Hugenholtz P, Tyson GW. CheckM: Assessing the quality of microbial genomes recovered from isolates, single cells, and metagenomes. Genome Res. 2015;25:1043–1055.

4. Chen Y, Chen Y, Shi C, Huang Z, Zhang Y, Li S, *et al*. SOAPnuke: A MapReduce acceleration-supported software for integrated quality control and preprocessing of high-throughput sequencing data. Gigascience. 2018;7:1–6.

5. Peng Y, Leung HCM, Yiu SM, Chin FYL. IDBA-UD: a *de novo* assembler for single-cell and metagenomic sequencing data with highly uneven depth. Bioinformatics. 2012;28:1420–1428.

6. Luo R, Liu B, Xie Y, Li Z, Huang W, Yuan J, *et al*. SOAPdenovo2: An empirically improved memory-efficient short-read de novo assembler. Gigascience. 2012;1:2047–217X.

7. Curson ARJ, Williams BT, Pinchbeck BJ, Sims LP, Martínez AB, Rivera PPL, *et al*. DSYB catalyses the key step of dimethylsulfoniopropionate biosynthesis in many phytoplankton. Nat Microbiol. 2018;3:430–439.

8. Kageyama H, Tanaka Y, Shibata A, Waditee-Sirisattha R, Takabe T. Dimethylsulfoniopropionate biosynthesis in a diatom *Thalassiosira pseudonana*: Identification of a gene encoding MTHB-methyltransferase. Arch Biochem Biophys. 2018;645:100–106.

9. Curson ARJ, Liu J, Bermejo Martínez A, Green RT, Chan Y, Carrión O, *et al.* Dimethylsulfoniopropionate biosynthesis in marine bacteria and identification of the key gene in this process. Nat Microbiol. 2017;2:1–9.

10. Williams BT, Cowles K, Bermejo Martínez A, Curson ARJ, Zheng Y, Liu J, *et al*. Bacteria are important dimethylsulfoniopropionate producers in coastal sediments. Nat Microbiol. 2019;4:1815–1825.

11. Howard EC, Henriksen JR, Buchan A, Reisch CR, Bürgmann H, Welsh R, *et al*. Bacterial taxa that limit sulfur flux from the ocean. Science. 2006;314:649–652.

12. Howard EC, Sun S, Biers EJ, Moran MA. Abundant and diverse bacteria involved in DMSP degradation in marine surface waters. Environ Microbiol. 2008;10:2397–2410.

13. Howard EC, Sun S, Reisch CR, del Valle DA, Bürgmann H, Kiene RP, *et al*. Changes in dimethylsulfoniopropionate demethylase gene assemblages in response to an induced phytoplankton bloom. Appl Environ Microbiol. 2011;77:524–31.

14. Todd JD, Rogers R, Li YG, Wexler M, Bond PL, Sun L, *et al*. Structural and regulatory genes required to make the gas dimethyl sulfide in bacteria. Science. 2007;315:666–669.

15. Curson ARJ, Fowler EK, Dickens S, Johnston AWB, Todd JD. Multiple DMSP lyases in the γ-proteobacterium *Oceanimonas doudoroffii*. Biogeochemistry. 2012;110:109–119.

16. Todd JD, Curson ARJ, Nikolaidou-Katsaraidou N, Brearley CA, Watmough NJ, Chan Y, *et al.* Molecular dissection of bacterial acrylate catabolism–unexpected links with dimethylsulfoniopropionate catabolism and dimethyl sulfide production. Environ Microbiol. 2010;12:327–343.

17. Curson ARJ, Sullivan MJ, Todd JD, Johnston AWB. Identification of genes for dimethyl sulfide production in bacteria in the gut of Atlantic Herring (*Clupea harengus*). ISME J. 2010;4:144–146.

18. Curson ARJ, Rogers R, Todd JD, Brearley CA, Johnston AWB. Molecular genetic analysis of a dimethylsulfoniopropionate lyase that liberates the climate-changing gas dimethylsulfide in several marine α-proteobacteria and *Rhodobacter sphaeroides*. Environ Microbiol. 2008;10:757–767.

19. Liu J, Liu J, Zhang S-H, Liang J, Lin H, Song D, *et al*. Novel insights into bacterial dimethylsulfoniopropionate catabolism in the East China Sea. Front Microbiol. 2018;9:3206.

20. Todd JD, Curson ARJ, Dupont CL, Nicholson P, Johnston AWB. The *dddP* gene, encoding a novel enzyme that converts dimethylsulfoniopropionate into dimethyl sulfide, is widespread in ocean metagenomes and marine bacteria and also occurs in some Ascomycete fungi. Environ Microbiol. 2009;11:1376–1385.

21. Todd JD, Curson ARJ, Kirkwood M, Sullivan MJ, Green RT, Johnston AWB. DddQ, a novel, cupin-containing, dimethylsulfoniopropionate lyase in marine roseobacters and in uncultured marine bacteria. Environ Microbiol. 2011;13:427–438.

22. Burkhardt I, Lauterbach L, Brock NL, Dickschat JS. Chemical differentiation of three DMSP lyases from the marine Roseobacter group. Org Biomol Chem. 2017;15:4432–4439.

23. Li C-Y, Wei T-D, Zhang S-H, Chen X-L, Gao X, Wang P, *et al*. Molecular insight into bacterial cleavage of oceanic dimethylsulfoniopropionate into dimethyl sulfide. Proc Natl Acad Sci. 2014;111:1026–31.

24. Todd JD, Kirkwood M, Newton-Payne S, Johnston AWB. DddW, a third DMSP lyase in a model Roseobacter marine bacterium, *Ruegeria pomeroyi* DSS-3. ISME J. 2012;6:223–226.

25. Curson ARJ, Sullivan MJ, Todd JD, Johnston AWB. DddY, a periplasmic dimethylsulfoniopropionate lyase found in taxonomically diverse species of Proteobacteria. ISME J. 2011;5:1191–1200.

26. van Der Maarel MJEC, Aukema W, Hansen TA. Purification and characterization of a dimethylsulfoniopropionate cleaving enzyme from *Desulfovibrio acrylicus*. FEMS Microbiol Lett. 1996;143:241–245.

27. Li CY, Zhang D, Chen XL, Wang P, Shi WL, Li PY, et al. Mechanistic insights into dimethylsulfoniopropionate lyase DddY, a new member of the cupin superfamily. J Mol Biol. 2017;429:3850–3862.

28. Lei L, Alcolombri U, Tawfik DS. DddY is a bacterial dimethylsulfoniopropionate lyase representing a new cupin enzyme superfamily with unknown primary function. bioRxiv. 2017;161257.

29. Curson ARJ, Todd JD, Sullivan MJ, Johnston AWB. Catabolism of dimethylsulphoniopropionate: microorganisms, enzymes and genes. Nat Rev Microbiol. 2011;9:849–859.

30. Sun J, Todd JD, Thrash JC, Qian Y, Qian MC, Temperton B, et al. The abundant marine bacterium Pelagibacter simultaneously catabolizes dimethylsulfoniopropionate to the gases dimethyl sulfide and methanethiol. Nat Microbiol. 2016;1:1–5.

31. Li CY, Wang XJ, Chen XL, Sheng Q, Zhang S, Wang P, et al. A novel ATP dependent dimethylsulfoniopropionate lyase in bacteria that releases dimethyl sulfide and acryloyl-CoA. Elife. 2021;10:e64045.

32. Alcolombri U, Ben-Dor S, Feldmesser E, Levin Y, Tawfik DS, Vardi A. Identification of the algal dimethyl sulfide-releasing enzyme: A missing link in the marine sulfur cycle. Science. 2015;348:1466–1469.

33. Carrión O, Curson ARJ, Kumaresan D, Fu Y, Lang AS, Mercadé E, et al. A novel pathway producing dimethylsulphide in bacteria is widespread in soil environments. Nat Commun. 2015;6:1–8.

34. Boden R, Borodina E, Wood AP, Kelly DP, Murrell JC, Schäfer H. Purification and characterization of dimethylsulfide monooxygenase from *Hyphomicrobium sulfonivorans*. J Bacteriol. 2011;193: 1250–1258.

35. Lee HH, Kim SJ, Shin HJ, Park JY, Yang JW. Purification and characterization of methyl mercaptan oxidase from *Thiobacillus thioparus* for mercaptan detection. Biotechnol Bioprocess Eng. 2002;7: 375–379.

36. Eyice Ö, Myronova N, Pol A, Carrión O, Todd JD, Smith TJ, et al. Bacterial SBP56 identified as a Cu-dependent methanethiol oxidase widely distributed in the biosphere. ISME J. 2018;12:145–160.

37. McDevitt CA, Hanson GR, Noble CJ, Cheesman MR, McEwan AG. Characterization of the redox centers in dimethyl sulfide dehydrogenase from *Rhodovulum sulfidophilum*. Biochemistry. 2002;41: 15234–15244.

38. Lidbury I, Kröber E, Zhang Z, Zhu Y, Murrell JC, Chen Y, et al. A mechanism for bacterial transformation of dimethylsulfide to dimethylsulfoxide: a missing link in the marine organic sulfur cycle. Environ Microbiol. 2016;18:2754–2766.

39. Satoh T, Kurihara FN. Purification and properties of dimethylsulfoxide reductase containing a molybdenum cofactor from a photodenitrifier, *Rhodopseudomonas sphaeroides f.s.* *denitrificans*. J Biochem. 1987;102:191–197.

40. Sambasivarao D, Weiner JH. Dimethyl sulfoxide reductase of *Escherichia coli*: An investigation of function and assembly by use of in vivo complementation. J Bacteriol. 1991;173:5935–5943.

41. McEwan AG, Ferguson SJ, Jackson JB. Purification and properties of dimethyl sulphoxide reductase from *Rhodobacter capsulatus*. A periplasmic molybdoenzyme. Biochem J. 1991;274:305.

42. Reisch CR, Stoudemayer MJ, Varaljay VA, Amster IJ, Moran MA, Whitman WB. Novel pathway for assimilation of dimethylsulphoniopropionate widespread in marine bacteria. Nature. 2011;473: 208–211.

43. Tan D, Crabb WM, Whitman WB, Tong L. Crystal structure of DmdD, a crotonase superfamily enzyme that catalyzes the hydration and hydrolysis of methylthioacryloyl-CoA. PLoS One. 2013;8:e63870.

44. Reisch CR, Crabb WM, Gifford SM, Teng Q, Stoudemayer MJ, Moran MA, et al. Metabolism of dimethylsulphoniopropionate by *Ruegeria pomeroyi* DSS‐3. Molecular Microbiology. 2013;89:774–791.

45. Wang P, Cao HY, Chen XL, Li CY, Li PY., Zhang XY, et al. Mechanistic insight into acrylate metabolism and detoxification in marine dimethylsulfoniopropionate‐catabolizing bacteria. Molecular microbiology. 2017;105:674–688.
